# Calendulaglycoside A showing potential activity against SARS-CoV-2 main protease: Molecular docking, molecular dynamics, and SAR studies

**DOI:** 10.1016/j.jtcme.2021.05.001

**Published:** 2021-05-17

**Authors:** Ahmed A. Zaki, Ahmed Ashour, Sameh S. Elhady, Khaled M. Darwish, Ahmed A. Al-Karmalawy

**Affiliations:** a Department of Pharmacognosy, Faculty of Pharmacy, Mansoura University, Mansoura, 35516, Egypt; b Department of Pharmacognosy, Faculty of Pharmacy, Horus University-Egypt, New Damietta, 34518, Egypt; c Department of Natural Products, Faculty of Pharmacy, King Abdulaziz University, Jeddah, 21589, Saudi Arabia; d Department of Medicinal Chemistry, Faculty of Pharmacy, Suez Canal University, Ismailia, 41522, Egypt; e Department of Pharmaceutical Medicinal Chemistry, Faculty of Pharmacy, Horus University-Egypt, New Damietta, 34518, Egypt

**Keywords:** COVID-19, C. officinalis L., Triterpenes, Computational studies, SAR

## Abstract

**Background and aim:**

The discovery of drugs capable of inhibiting SARS-CoV-2 is a priority for human beings due to the severity of the global health pandemic caused by COVID-19. To this end, natural products can provide therapeutic alternatives that could be employed as an effective safe treatment for COVID-19.

**Experimental procedure:**

Twelve compounds were isolated from the aerial parts of *C. officinalis* L. and investigated for their inhibitory activities against SARS-CoV-2 M^pro^ compared to its co-crystallized N3 inhibitor using molecular docking studies. Furthermore, a 100 ns MD simulation was performed for the most active two promising compounds, Calendulaglycoside A (SAP5) and Osteosaponin-I (SAP8).

**Results and conclusion:**

At first, molecular docking studies showed interesting binding scores as compared to the N3 inhibitor. Calendulaglycoside A (SAP5) achieved a superior binding than the co-crystallized inhibitor indicating promising affinity and intrinsic activity towards the M^pro^ of SARS-CoV-2 as well. Moreover, findings illustrated preferential stability for SAP5 within the M^pro^ pocket over that of N3 beyond the 40 ns MD simulation course. Structural preferentiality for triterpene-M^pro^ binding highlights the significant role of 17*β*-glucosyl and carboxylic 3*α*-galactosyl I moieties through high electrostatic interactions across the MD simulation trajectories. Furthermore, this study clarified a promising SAR responsible for the antiviral activity against the SARS-CoV-2 M^pro^ and the design of new drug candidates targeting it as well. The above findings could be promising for fast examining the previously isolated triterpenes both pre-clinically and clinically for the treatment of COVID-19.

## Highlights

• Calendulaglycoside A (5) achieved a superior binding than the co-crystallized inhibitor towards the M^pro^ of SARS-CoV-2.
• Structural preferentiality for triterpene-M^pro^ binding highlights the significant role of 17*β*-glucosyl and carboxylic 3*α*-galactosyl I moieties through high electrostatic interactions.
• This study clarified a promising SAR responsible for the antiviral activity against the SARS-CoV-2 M^pro^.

## Graphical abstract

**Figure:**
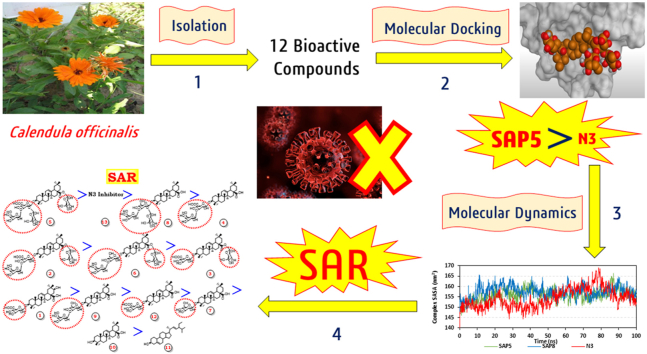

## List of abbreviations

**Abbreviation Meaning**
SARS-CoV-2: Severe Acute Respiratory Syndrome - Corona Virus - 2
COVID-19: 2019 novel coronavirus
M^pro^: Main protease
MD: Molecular dynamics
SAR: Structure-Activity relationships
PDB: Protein data bank
PME: Particle Mesh Ewald
RMSD: Root-mean-square deviation
RMSF: Root-mean-square fluctuation
SASA: Solvent accessible surface area
Rg: Radius of gyration
VMD: Visual Molecular Dynamics 1.9.3
MM/PBSA: Molecular Mechanics Poisson Boltzmann Surface Area

## 1 Introduction

The existence of the COVID-19 pandemic in the world demands the need to identify and characterize new drug candidates to address the health problem caused by the SARS-CoV-2.^1^^,^^2^ After SARS-CoV-2 infection, two overlapping polyproteins are produced (replicase 1a and 1 ab) which create many functional subunits for viral replication. This process requires two internally encoded proteases that cleave the aforementioned polyproteins at specific regions. The main protease or 3C-like protease (3CL^pro^) is one of these proteolytic enzymes that catalyze most events of viral maturation. Noteworthy, 3CL^pro^ is a crucial enzyme required for viral replication and so represents one of the most important drug targets towards SARS-CoV-2.^1^^,^^3^^,^^4^

Natural products from plant origin contributed largely to the drug discovery process. About 40% of FDA-approved drugs to date are either natural products or one of their derivatives. Also, traditional Chinese medicine and related herbs were used in China and reported to be successful in the management of COVID-19 patients. Moreover, many suggestions were made on phytochemicals of Indian herbs as anti-COVID-19 drugs as well.^5^, ^6^, ^7^, ^8^ Many natural compounds containing emodin as their active constituent have been reported to block the binding of SARS-CoV S protein to ACE2 receptor. Also, xanthoangelol G extracted from *Angelica keiskei* was found to inhibit a trypsin-like serine protease *in vitro*. Hesperetin, lignoid, terpenoid, tanshinone, and chalcone were reported to inhibit SARS-CoV 3CL^pro^ with promising IC_50_ values. Moreover, cryptotanshinone and tanshinone IIA were found to be excellent inhibitors of papain-like protease (PL^pro^) of both SARS-CoV and SARS-CoV-2. Furthermore, flavonoid compounds like kaempferol and kaempferol 3-O-α-l-arabinopyranoside were found to block the SARS-CoV viroporin 3a protein ion channel activity. Moreover, a molecular docking study discussed the proposed binding of rutin with M^pro^, RdRp, PL^pro^, and S-proteins of SARS-CoV-2 which its anti-SARS-CoV-2 activity was confirmed *in vitro* as well.^9^, ^10^, ^11^, ^12^

The genus (*Asteraceae*) includes approximately 25 herbaceous annual or perennial species, most common being *Calendula officinalis* L., *C. arvensis* L., *C. suffruticosa* Vahl., *C. stellata* Cav., *C. alata* Rech., *C. tripterocarpa* Rupr.^13^ The genus is native to the Mediterranean countries.^14^

C. officinalis L. (Pot marigold) has been used traditionally in the treatment of inflammations of internal organs, gastrointestinal ulcers, and dysmenorrhea and as a diuretic and diaphoretic in convulsions. It is also used for inflammations of the oral and pharyngeal mucosa, wounds, and burns.^15^ A tincture of the flowers suppressed the replication of herpes simplex, influenza Alpha 2 (A2), and influenza APR-8 (purified, and UV-inactivated) viruses *in vitro*.^16^

Medicinal properties of C. officinalis have been mentioned in the Ayurvedic and Unani systems of medicine indicating that leaves and flowers are antipyretic, anti-inflammatory, antiepileptic, and antimicrobial.^17^ In traditional and homeopathic medicine, C. officinalis has been used for poor eyesight, menstrual irregularities, varicose veins, hemorrhoids, and duodenal ulcers.^18^ In the middle ages, Calendula flowers were used for liver obstructions, snake bites and to strengthen the heart. It was used in the 18th century as a remedy for headaches, jaundice, and red eyes. The plant was employed in the civil war to treat wounds and as a remedy for measles, smallpox, and jaundice.^13^

Furthermore, sesquiterpene glycosides of *Calendula* were documented to inhibit the replication of the Herpes virus and rhinovirus.^19^^,^^20^ Also, *Calendula* extract exhibited antiviral activity against influenza and herpes simplex viruses.^21^

Nowadays, computational drug design methods such as molecular docking and molecular dynamics are very promising tools in the discovery of new drug candidates.^22^ Also, bioinformatics techniques opened new horizons to detect the crucial key amino acids at nearly identical physiological conditions, and so confirm greatly the results of computational methods. However, new drug candidates can be introduced depending on the chemical nature of the drug and its target receptor, saving effort and cost as well.^23^

Therefore, as an extension to our previously promising work,^24^, ^25^, ^26^, ^27^, ^28^, ^29^ and depending on the crucial role of COVID-19 main protease (M^pro^) for the viral activity and replication besides the aforementioned antiviral efficacy of *C. officinalis*, we have isolated eleven triterpenes and one sterol (Figure SI1, supplementary data) and evaluated their binding affinities against the M^pro^ of SARS-CoV-2 via molecular docking (PDB ID: 6LU7).^30^ Besides, molecular dynamics simulation was performed on the best-docked ligand-protease complexes to obtain a deep understanding of the obtained affinity in the explicit solvent model and to get more reliable results on the stability of the tested compounds within the active pocket of protease and consequently confirming the results of docking.

## 2 Experimental

### 2.1 General experimental procedure

Both 1D and 2D NMR experiments were measured on Varian 400 MHz spectrometer. The samples were dissolved in deuterated methanol (CD_3_OD). The sample peaks were adjusted with the reference to the solvent peak. The high-resolution mass spectra were measured on Agilent Technologies 6200 series mass spectrometer. Stationary phases for column chromatography (CC); flash silica gel of particle size 32-63μ (Dynamic adsorbents Inc.) and RP-18 (Polar bond, J. T. Baker) were used. Silica gel F_254_ aluminum sheet (Fluka) and Silica 60 RP-18 F_254_S aluminum sheet (Merck) were employed for performing TLC. The spots were noticed under UV-254 and 365 nm or after spraying with 1% vanillin/H_2_SO_4_ followed by heating for 2 min. All solvents used through the chromatographic process were of analytical grade (Fischer Chemicals).

### 2.2 Plant material

The plant *C. officinalis* L. was collected from the farm of Pharmacognosy Department, Faculty of Pharmacy in April 2014 and identified in Botany Department, Faculty of Sciences, Mansoura University, Mansoura 35516, Egypt. A plant sample under the code (No. 1866) was deposited in the Department of Pharmacognosy, Faculty of Pharmacy, Mansoura University.

### 2.3 Extraction and isolation

The air-dried and powdered aerial parts of *C. officinalis* L. (500 g) were extracted till exhaustion with methanol at room temperature. The collected solvent was evaporated to dryness using a rotary evaporator. The combined crude extract (total, 67 g) was suspended in 150 mL water and defatted with hexanes (0.5 L x 4) to yield the hex-fraction (7.8 gm) and 57 gm representing the remaining extract. The silica gel column of hex-fraction using the gradients of 5–25% ethyl acetate in petroleum ether resulted in the isolation of compound 11 (17 mg).

The weight of 57 gm remaining extract was fractionated over a VLC of RP-18 silica and eluted with a gradient of 10–100% MeOH/H_2_O to give 13 fractions (I - XIII). Fractions III and V were separately applied to silica gel column chromatography and eluted with CHCl_3_: MeOH: H_2_O (6.5: 3.5: 1) to yield 1 and 5, respectively. Fraction VIII was subjected to a repeated silica gel column and eluted with the mobile phase EtOAc: CHCl_3:_ MeOH: H_2_O (6: 4: 4: 1) to afford 3 and 4. The silica gel column of fraction VII and isocratic elution with CHCl_3_: MeOH: H_2_O (6.5: 3.5: 1) resulted in purification of 2. Fraction XI was subjected to silica gel CC and eluted with EtOAc: CHCl_3:_ MeOH: H_2_O (6: 4: 4: 1) then CHCl_3_: MeOH: H_2_O (6.5: 3.5: 1) to afford 6 and 7, respectively. Fraction XII was applied to the silica gel column and using the eluent CHCl_3_: MeOH: H_2_O (6.5: 3.5: 1) to afford 8, 9, and 10. The column chromatography (1.0 × 85 cm) of fraction XIII (270 mg) over normal phase silica gel using elution systems; EtOAc: CHCl_3:_ MeOH: H_2_O (6: 4: 4: 1) and CHCl_3_: MeOH: H_2_O (6.5: 3.5: 1) to afford compound 12 (11.7 mg). Compound 12 was further purified from impurities over Luna C_18_ column (150 × 4.6 mm, 5 μm particle size; Phenomenex, Inc.)- HPLC (Waters Alliance 2795), equipped with PDA detector. The mobile phase composed of acetonitrile HPLC grade containing 0.1% formic acid (A) and water HPLC grade containing 0.1% formic (B) in a gradient mode: A/B 20/80 for 5 min, A/B 35/65 for 15 min, A/B 35/65 for 15 min, A/B 45/55 for 25 min, and A/B 55/45 for the next 20 min at a rate of 1 mL/min to get 12 (4.1 mg, R_*t*_ 32.468).

### 2.4 *In silico* studies

The twelve isolated compounds were examined for their binding potentials towards SARS-CoV-2 main protease in comparison to its N3 inhibitor via molecular docking through MOE 2019 suite^31^ and molecular dynamics simulation using a GROMACS-2019 software package and CHARMM36 force field.^32^

#### 2.4.1 Docking study

##### 2.4.1.1 Isolated tested triterpenes preparation

The chemical structures of the isolated compounds were sketched in their 3D forms using MOE 2019 sketcher and then prepared for docking as the default procedure.^33^^,^^34^ A database containing our tested compounds together with the co-crystallized (N3) inhibitor was saved as an MDB file for docking against the SARS-CoV-2 main protease.

##### 2.4.1.2 The target SARS-CoV-2 main protease preparation

The protein data bank was used to obtain the crystal structure of the SARS-CoV-2 main protease (M^pro^). The selected enzyme was downloaded containing its co-crystallized (N3) inhibitor (PDB code 6LU7).^30^ It was protonated where hydrogen atoms were added with their 3D geometry, corrected for any found errors in the connection or type of different atoms, and then energy minimized at the end of the preparation steps. Moreover, Site Finder was applied to define and isolate the same binding pocket of the co-crystallized native inhibitor (N3) as dummy atoms for the docking step.^35^^,^^36^

##### 2.4.1.3 Docking of the isolated triterpenes to the viral main protease binding site

At first, a validation process to ensure the accuracy of our docking program was run, and the valid behavior of the co-crystallized (N3) inhibitor was confirmed by a low RMSD value of 1.23 Å as depicted in the supplementary data (Figure SI2).^37^^,^^38^ The following methodology was performed: the previously prepared active site was loaded, and the docking process was started as a general one. The docking site was adjusted as dummy atoms, the forcefield was selected to be Amber10:EHT, the rigid receptor was selected as refinement methodology and GBVI/WSA dG as its scoring methodology, the placement methodology was as triangle matcher and its scoring methodology was applied as London dG. The prepared MDB file containing the twelve prepared medicinal plant ligands (1- 12) together with the co-crystallized inhibitor (N3, 13) was loaded and then the general dock calculations were run automatically^39^ to select poses with the best scores, rmsd_refine values, and binding modes.

#### 2.4.2 Molecular dynamics simulation

The top-dock models of the most promising leads (Calendulaglycoside A (SAP5) and Osteosaponin-I (SAP8)), as well as N3 in complex with SARS-CoV-2 main protease, were chosen as starting coordinates for 100 ns all-atoms molecular dynamic (MD) simulation using a GROMACS-2019 software package (GNU, General Public License; http://www.gromacs.org) and CHARMM36 force field.^32^,^28^^,^^29^ Regarding the investigated ligands, the CHARMM force field parameters were automatically generated using the CHARMM General Force Field (CGenFF) program (ParamChem project; https://cgenff.umaryland.edu/).^40^ Each ligand-protein complex was then solvated within a cubic box of the transferable intermolecular potential with a three-points (TIP3P) water model (100 × 100 × 100 Å) allowing a minimum of 10 Å marginal distance between protein and each side of the 3D box.^41^ Under periodic boundary conditions implementation, the protein residues were assigned for their standard ionization states at physiological conditions (pH 7.0), and the whole complexes were neutralized via sufficient numbers of K‏^+^ and Cl^−^ ions added via Monte-Carlo ion-placing method.^42^ The total number of atoms, including water and ions, for each final system, was 89112, 85645, and 85577 atoms for N3, SAP5, and SAP8 systems, respectively. The MD simulation was conducted over three stages (minimization, equilibration, and production) and a 1000 kJ/mol.nm^2^ force constant was used along minimization and equilibration stages for restraining all heavy atoms and preserving original protein folding.^43^ The first stage involved initial optimization of each system geometry using 5000 iterations (5 ps) with the steepest descent algorithm. The subsequent step involved system equilibration for 125 ps under constant Number of particles, Volume, and Temperature (NVT) ensemble, then 125 ps under constant pressure (NPT ensemble) guided by the Berendsen temperature coupling method and Parrinello-Rahman barostat, respectively for regulating the temperature within 3D box.^44^^,^^45^

Finally, the MD simulations were run for 100 ns under NPT ensemble, where the isothermic and isobarometric conditions were maintained at 303.15 K temperature and 1 atm pressure using the Parrinello-Rahman barostat. Long-range electrostatic interactions were computed using Particle Mesh Ewald (PME) algorithm,^46^ while the implemented linear constraint LINCS method constrained all covalent bond lengths, including hydrogens, allowing an integration time step size of 2 fs and without any restriction.^47^ The non-bounded interactions, Coulomb and van der Waals interactions were truncated at 10 Å using the Verlet cut-off scheme.^48^

Computing comparative data, including root-mean-square deviation (RMSD), difference root-mean-square fluctuation (ΔRMSF), solvent accessible surface area (SASA), and radius of gyration (Rg), were performed through analyzing MD trajectories using the GROMACS built-in tools and. For better estimation of the protein flexibility, the ΔRMSF was estimated for each ligand-bound protein relative to the apo state of SARS-CoV-2 M^pro^ (PDB ID: 6y84; atomic resolution 1.39 Å), where ΔRMSF = apo RMSF – holo RMSF. The same above preparation, minimization, equilibration procedures as well as the 100 ns all-atom MD simulation production were applied to the M^pro^ apo state, except no ligand preparation included. To investigate the hydrogen bond interactions between the ligands and corresponding target, initially, the Visual Molecular Dynamics 1.9.3 (VMD) package (the University of Illinois at Urbana-Champaign, USA) “Hydrogen bonds” tool was used to explore the established ligand-protein hydrogen bond interactions and their relative frequencies.^49^ The cut-off values for hydrogen bond (Hydrogen bond-Donor … Acceptor; H-D … A) distance and angle were assigned at 3.0 Å and 20°, respectively, being optimum for hydrogen bonding strength. For the above-identified ligand/protein hydrogen bond pairs, the VMD's “Distance Calculation” tool to monitor the time evolution of the above-identified significant hydrogen bonds corresponding to specified ligand/protein atoms (H-D … A) over the whole simulation period. The Pymol graphical software ver. 2.0.6 (Schrödinger™, NY, USA) was utilized for figure generation of ligand-protein binding interaction analysis.^50^

Finally, the binding-free energy between the ligand and protein was estimated via the Molecular Mechanics Poisson Boltzmann Surface Area (MM/PBSA) calculation using the GROMACS “g_mmpbsa” module.^51^ Important MM/PBSA parameters for polar/solvation calculations were set as; dielectric constants of solute (2 pdie), solvent (80 pdie), and reference-vacuum (1 vdie) as well as solvent probe radius (1.4 Å). Regarding SASA apolar/non-polar solvation; solvent surface tension, SASA-solvent probe radius, and offset constant were set at 0.0226778 kJ/mol.Å^2^, 1.4 Å, and 3.84928 kJ/mol, respectively. Finally, parameters for continuum/integral based model (WCA-like) were set at solvent probe radius 1.25 Å, bulk solvent density (0.033428 Å^−3^), and the number of quadrature points per Å^2^ of 200. All MM/PBSA calculations were applied on selected representative frames (100 snapshots per investigated time interval) using the GROMACS “*gmx trjconv*“and “*gmx trjcat*“ command lines.

#### 2.4.3 Molecular properties, Lipinski rule, and ADME studies

Both the pharmacokinetic and pharmacodynamic properties which play an essential role in the optimization of lead molecules were studied for the tested isolated molecules using the Swiss ADME server. Prediction of ADME (Absorption, Distribution, Metabolism, and Elimination) using *in silico* techniques is a fast effective way as an alternate for experimental estimations. Bioavailability radar and Lipinski's rule of five were calculated for all the tested compounds to evaluate their oral bioavailability. Moreover, blood-brain barrier (BBB) permeability and different isoforms of cytochrome p450 interactions which play an important role in the drug elimination were evaluated as well.^52^

## 3 Results and discussions

### 3.1 Compounds isolated from *C. officinalis*

Using the compounds; Machaerinic acid 3-*O*-*β*-d-glucuronopyranoside (1),^53^ Calendulaglycoside C (2),^54^ Glycoside F (3),^55^ Calenduloside G (4),^56^ Calendulaglycoside A (5),^57^ Calenduloside B (6),^54^ Glucoside I (7),^58^ Osteosaponin-I (8),^59^ Arvensoside B (9), Oleanolic acid (10)^58^ and Stigmasterol (11), which are previously isolated by Zaki and Qiu^53^ as standard metabolites in the chromatographic re-investigation of total extract of *C. officinalis*, compound 12 was isolated. It was obtained as a white powder with the molecular formula of C_36_H_56_O_9_ consistent with ion peak at *m/z* [M+H]^+^ 633.4009 (cal.C_36_H_57_O_9_ 633.4003). The study of NMR data including C,^13^ H^1^, DEPT-135, HMQC, and HMBC of 12, and comparing with those published in the literature, revealed the triterpene structure of oleanolic acid. Additional six peaks are attributed to the glucuronic acid moiety. The structure of 12 was concluded to be Oleanolic acid 3-glucuronide, which is previously reported by Zhang et al., 2014 in *C. officinalis* flowers.^60^

### 3.2 Docking study

The SARS-CoV-2 main protease catalytic dyad is composed of Cys–His, and the binding site is located between subunits I and II.^61^ Molecular docking of the compounds (1-12) and N3 inhibitor 13 into M^pro^ active site was done. They got stabilized at the inhibitor binding site by diverse interactions (Table 1). The obtained molecular docking results were considered valid since redocking the crystalized ligand (N3) furnished great ligand superposition with root-mean standard deviation (RMSD) below 2 Å.^62^ The descending strength order based on the score values: (5) ˃ N3 inhibitor (13, redocked) ˃ (8) ˃ (4) ˃ (2) ˃ (6) ˃ (3) ˃ (1) ˃ (9) ˃ (12) ˃ (7) ˃ (10) ˃ (11). Surprisingly, SAP5 was more promising with a binding score of (−9.90 kcal/mol) compared to the N3 inhibitor (−9.37 kcal/mol).

**Table 1:** Binding scores, RMSD-refine values, and amino acid interactions of the tested triterpenes (1-12), and the redocked N3 inhibitor 13 into its binding site of SARS-CoV-2 main protease.

Tested compound	S^a^ Kcal/mole	RMSD_refine^b^	Amino acid interaction	Distance A֯
1	−7.60	1.74	Glu166/H-acceptor Gln189/H-donor	2.95 3.50
2	−8.89	1.95	Glu166/H-acceptor Glu166/H-acceptor Glu166/H-acceptor Asn142/H-acceptor	2.80 3.04 3.49 3.31
3	−8.02	1.82	Glu166/H-donor Glu166/H-acceptor	2.79 2.89
4	−9.07	2.18	Thr26/H-acceptor Glu166/H-acceptor	3.09 3.20
5	−9.90	1.89	Glu166/H-acceptor Leu167/H-acceptor Asn119/H-donor	2.96 2.99 3.29
6	−8.86	2.32	Glu166/H-acceptor Thr24/H-acceptor Thr26/H-acceptor Asn142/H-donor	2.95 3.17 3.29 3.42
7	−7.05	2.38	Thr26/H-acceptor Gly143/H-donor	3.06 3.11
8	−9.20	1.91	Gln189/H-acceptor Glu166/H-acceptor Asn142/H-acceptor Glu166/H-acceptor His163/H-donor	2.91 2.99 3.03 3.11 3.30
9	−7.58	2.09	Glu166/H-acceptor Glu166/H-acceptor	3.02 3.52
10	−6.51	2.06	Gln189/H-acceptor	2.61
11	−6.46	1.63	Glu166/H-acceptor	3.07
12	−7.45	1.98	Glu166/H-donor Gln189/H-acceptor Gln189/H-acceptor Met165/H-acceptor Met49/H-acceptor	2.95 3.04 3.39 3.80 4.32
13, N3	−9.37	1.90	Glu166/H-donor Gln189/H-acceptor Gly143/H-donor Asn142/H-donor	2.88 2.92 3.10 3.24

a S: the score of a compound inside the protein binding site applying the London dG scoring method.

b RMSD_Refine: the root-mean-squared-deviation after and before refinement between the predicted pose and the crystal structure, respectively.

Scores, RMSD_refine values, and diverse interactions with the amino acids of the M^pro^ pocket are described in Table 1. Also, their different 3D photos are represented in the supplementary data (Figure SI2).

Studying the obtained docking results of the tested triterpenes, it was obvious that many of them were very close in both binding scores and modes compared to the co-crystallized (N3) inhibitor especially 5, 8, 4, 2, and 6 compounds with binding scores of −9.90, −9.20, −9.07, −8.89, and −8.86 kcal/mol, respectively (Table 1). It is worth mentioning that the binding energy value of compound SAP5 (S = −9.90 kcal/mol) was higher than that of the redocked co-crystallized inhibitor (S = −9.37 kcal/mol) indicating a very promising binding affinity and an expected intrinsic activity as well. Moreover, the remaining tested compounds showed very close and promising binding scores to the redocked N3 inhibitor as well. The binding modes of the isolated compounds (1-12) and the redocked N3 (13) were described in detail in Table 1. Furthermore, the 2D and 3D binding interactions, surface and maps, and 3D protein pocket positioning for all the tested compounds are represented in the Supplementary data (Figures SI3 and SI4). However, the 3D binding interactions and the 3D pocket positioning of the best selected two isolated triterpene compounds (SAP5 and SAP8) compared to the redocked N3 inhibitor (13) are represented in Table 2.

**Table 2:** 3D pictures representing both the binding modes and the positioning inside the protein pocket of the SARS-CoV-2 main protease between the best-selected triterpenes (SAP5 and SAP8), and the N3 inhibitor (redocked, 13). H-bonds are represented by red dashed lines while H-pi bonds by black ones.

Compound	3D interaction	3D protein positioning
SAP5		
SAP8		
N3, 13		

### 3.3 Molecular dynamics simulation

Adopting MD simulation is considered an efficacious approach to explore the stability of the predicted ligand/protein complex, obtained from previous docking studies, as well as investigate their relative dynamic nature providing stability information of the predicted binding interactions with important residues.^63^ In these regards, both SAP5 and SAP8, in complex with SARS-CoV-2 M^pro^, were enrolled within 100 ns all-atom MD simulation runs.

#### 3.3.1 Stability profile investigation and structural drift

Using GROMACS “*gmx rmsd*” tool, stability of the ligand/protein complexes were explored in term of RMSD for both proteins and ligands over simulation frame. Typically, RMSD is a standard measurement for the structural distances between coordinates inferring the extent of deviation for a group of atoms relative to their reference structures.^64^ The RMSD values illustrate how much the conformations of these groups of atoms were changed providing a good indication for ligand and/or protein stability as well as validate the adopted MD simulation course.^65^

**Fig. 1:**
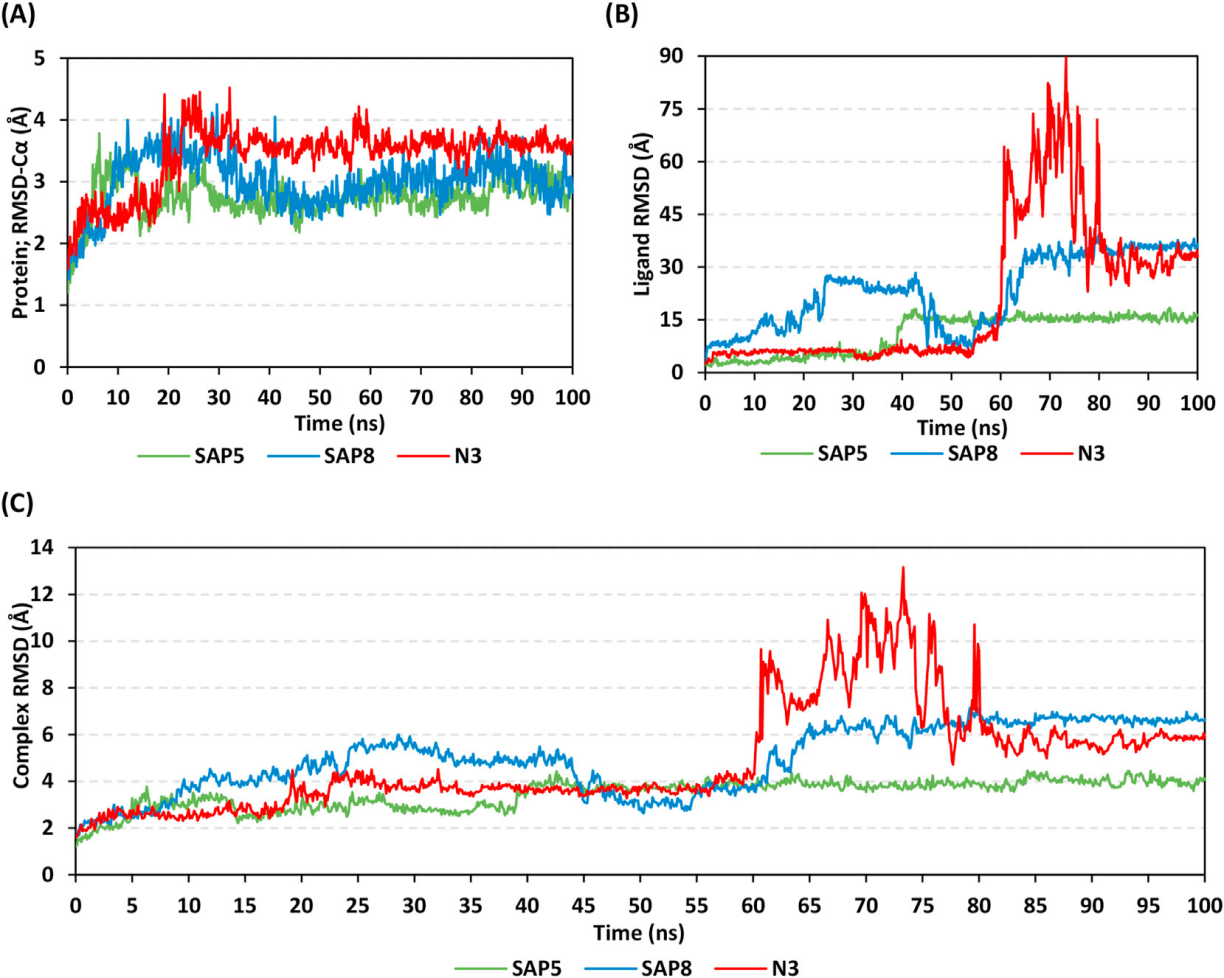
Time evolution RMSD trajectories of the three investigated ligand-protein complexes over 100 ns all-atom MD simulation. (A) protein RMSD relative to its backbone; (B) ligand backbone RMSD; (C) protein-ligand complex backbone RMSD, as a function of the MD simulation time (ns). Trajectories for SAP5/protein, SAP8/protein, and N3/protein systems are represented in green, blue, and red, respectively.

In the presented study, the three proteins under investigation were successfully converged within the 100 ns MD simulation window. The estimated C-alpha RMSD (RMSD-Cα) trajectories of SAP5-bound protein were of the lowest maxima showing minimal fluctuations as compared to those of SAP8 and N3 (Fig. 1A). The SAP5-bounded protein started from RMSD-Cα 1.21 Å where gradually increases until it was converged at around 14 ns with an RMSD-Cα value of 2.1 Å during simulation. After this, the protein adopted an equilibrium plateau showing minimal fluctuations around its average till the end of the MD run with a final RMSD-Cα value of 2.86 Å. This behavior is typical for MD simulation runs where the protein starts to relax following the removal of all the constraints till reaching its equilibration state where the RMSD-Cα trajectories level off indicating the stability of the protein till the end of the simulation. Regarding both SAP8 and N3, the proteins started at higher RMSD-Cα values (1.80 and 1.59 Å, respectively), while only N3-bound protein depicted late convergences following 30 ns from the start of the MD simulation runs. Following convergence, both proteins were dynamically equilibrated showing their RMSD-Cα trajectories being maintained around their respective minimal deviations around ∼2.60 and ∼3.50 Å, respectively. Notably, the SAP8-bound proteins showed comparable RMSD-Cα tones in respect to those of SAP5 where the final SAP8-bound protein reached its RMSD-Cα value of 2.83 Å at the end of MD simulation. However, the RMSD-Cα trajectories for N3-bound protein were significantly higher following convergence reaching a final RMSD of 3.67 Å at 100 ns.

The presented findings infer the superior stability of SAP5-bound protein, which was further correlated to its ligand's backbone RMSD trajectories. Well-behaved simulation was depicted for SAP5 where it rapidly reached its dynamic equilibration, following several initial nanoseconds, where the subsequent RMSD-Cα tones were maintained below 5 Å with depicted minimal fluctuations till 40 ns (Fig. 1B). Following 40 ns, SAP5 showed a significant RMSD-Cα increase up to where this was maintained till reaching the end of the MD simulation. The latter behavior suggested that SAP5 adopted a new conformation throughout the MD simulation course following a 40 ns run. On the contrarily, the SAP8 backbone RMSD showed a gradual increase till convergence at around 25 ns where RMSD levels off till reaching 43 ns where RMSD decreases then rises again reaching a final and final convergence around an average RMSD (35.67 Å) starting from 65 ns and till the end of MD simulation at 100 ns. These findings infer the adaptation of more than one conformation by SAP8 throughout the designated MD simulation suggesting ligand instability within the M^pro^ active site.

Concerning the N3 RMSDs, initial convergence was seen for a long time reaching 60 ns where after this, much higher fluctuations were depicted reaching up to a maximum RMSD of 90 Å indicates the ligand's diffusion far away from its initial binding pocket and probably towards the solvent side. Notably, N3 showed a relevant final dynamic equilibration with the following 80 ns of the simulation around a high mean RMSD value (29.32 Å), indicating the ligand convergence at a new final conformation. Investigating the backbone RMSD of each ligand-protein complex further ensures the superior stability of SAP5, as compared to SAP8 and N3, within its respective protein main pocket (Fig. 1C). Comparable complex RMSD tones were depicted relative to each corresponding ligand RMSD trajectories, indicating the great impact of the ligand RMSDs up on the complex stability and dynamic behavior across the MD simulation.

For further validation of MD simulation and ligand/protein stability, the principal component analysis (PCA) was performed to validate and monitor the MD simulation convergence. Throughout the PCA approach, the complexed protein's collective dynamic motion/behavior was evaluated across the equilibrium intervals of each ligand/protein MD simulation trajectories. This approach depends on constructing and diagonalizing covariance matrix from the protein's Cα atomic coordinates to capture strenuous atom motions using eigenvalues and eigenvectors.^64^, ^65^, ^66^^,^^63^, ^64^, ^65^^,^^62^, ^63^, ^64^^,^^62^, ^63^, ^64^^,^^62^, ^63^, ^64^^,^^62^, ^63^, ^64^ (Schreiner, Karch et al., 2012, David and Jacobs 2014, Arnittali, Rissanou et al., 2019)^63^, ^64^, ^65^ [59–61] Generally, the eigenvector of the covariance matrix correlates the overall atom's motion direction, while, eigenvalue represents the values of atom-wise contributions within the motion. GROMACS “*gmx covar*” command was used constructing and diagonalizing the covariance matric, whereas, “*gmx anaeig*” was for visualizing the most dominant modes (eigenvectors 1 and 2) besides calculating the trajectory coordinates/principal components overlap.

**Fig. 2:**
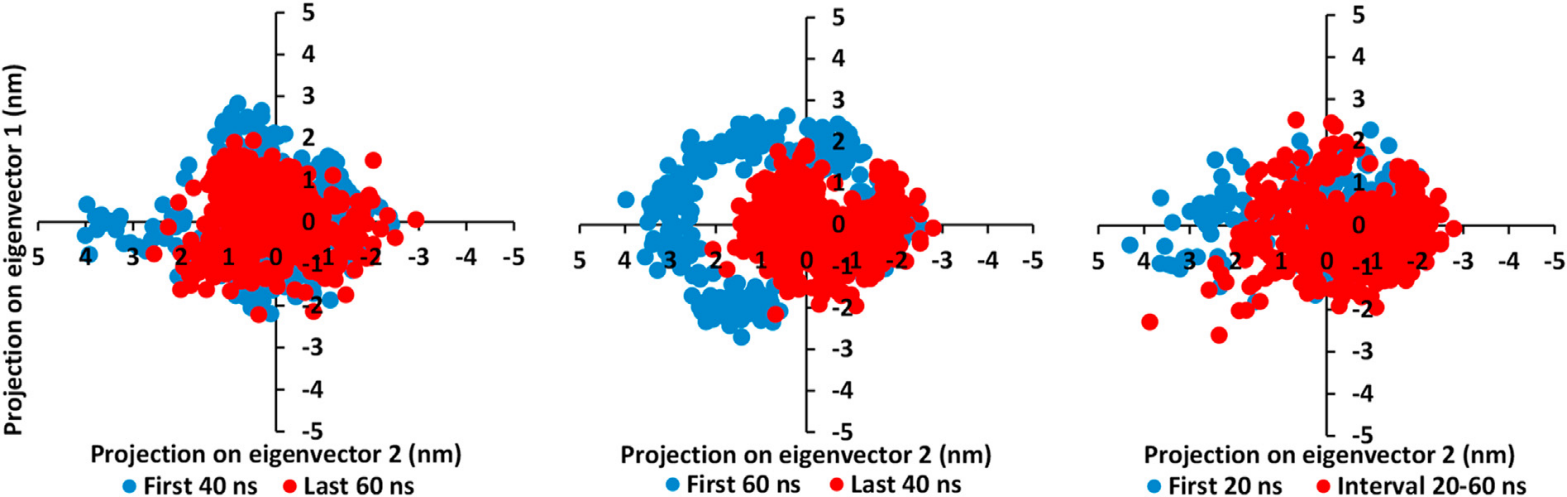
Projection of protein atoms in phase space along the first two dominant eigenvectors (eigenvector-1 and eigenvector-2). (A) SAP5/protein; (B) SAP8/protein; (C) N3/protein. The PCA calculations were conducted cross initial and last equilibrated intervals of MD simulation trajectories, having exhibiting differential expected structural stability and convergence.

With the corresponding eigenvalues indicating the dynamic behavior and degree of fluctuations, lower covariance matrix trace values confer with minimal escalation within the collective protein motion and so denoting MD simulation convergence.^67^, ^68^, ^69^ For SAP5-bound protein, the PCA was applied on the MD trajectories at the last 60 ns and comparing it with the initial MD simulation frames (first 40 ns). The latter approach would allow efficient monitoring as well as validating the MD simulation convergence since stable complex RMSD trajectories were obtained along the last 60 ns. Notably, lower covariance matrix average trace values were depicted at the last 60 ns for the investigated binder conferring successful protein convergence (Fig. 2A). This was obvious since the conformational space covered by the protein along the initial MD frames was wider. Covariance matrix trace values of 3.33 nm^2^ and 6.10 nm^2^ were assigned for the trajectories along the last 60 ns and initial frames, respectively. Concerning the other saponin binder, SAP8, higher differential covariance matrix trace values were obtained for the protein's atoms within the last 40 ns and initial time frame (4.43 nm^2^ and 7.62 nm^2^, respectively) (Fig. 2B). The latter dynamic behavior confers a higher comparative ligand/protein complex stability for SAP5 in comparison to that of SAP8. This came in good agreement with the significantly higher SAP8 RMSDs across the whole MD simulation (Fig. 1B and C).

The convergence of N3-bounded protein was also confirmed at the equilibration interval (25–60 ns) as compared to the initial frames (Fig. 2C). The depicted Covariance matrix trace values were slightly lower for the 20–60 ns equilibration frames relative to the initial ones (5.16 nm^2^ and 4.22 nm^2^ for initial and 20–60 ns frames, respectively). All the above findings ensure the stability of the bounded protein atoms at the last equilibrated intervals for the three investigated systems which in turn confer a validated convergence of the adopted MD simulation runs. Furthermore, the PCA approach highlights the suitability of the 100 ns MD simulation time course for investigating the stability of the saponin-based compounds at the target protein pocket.

#### 3.3.2 Conformational flexibility and root-mean-square fluctuation of target residues

For gaining more insights regarding the stability of the complex binding site, the ΔRMSF was estimated for each ligand-bound protein relative to the M^pro^ apo state along the whole MD simulation trajectories. The individual backbone RMSF of each protein was estimated using the GROMACS “*gmx rmsf”* command line. RMSF estimates the time evolution of the average deviation for each residue from its reference position within the minimized starting structures.^70^ Adopting ΔRMSF cut-off value of 0.3 Å was relevant for estimating the significant change within structural movements, where residues with >0.3 ΔRMSF values were considered of decreased mobility.^71^^,^^72^

**Fig. 3:**
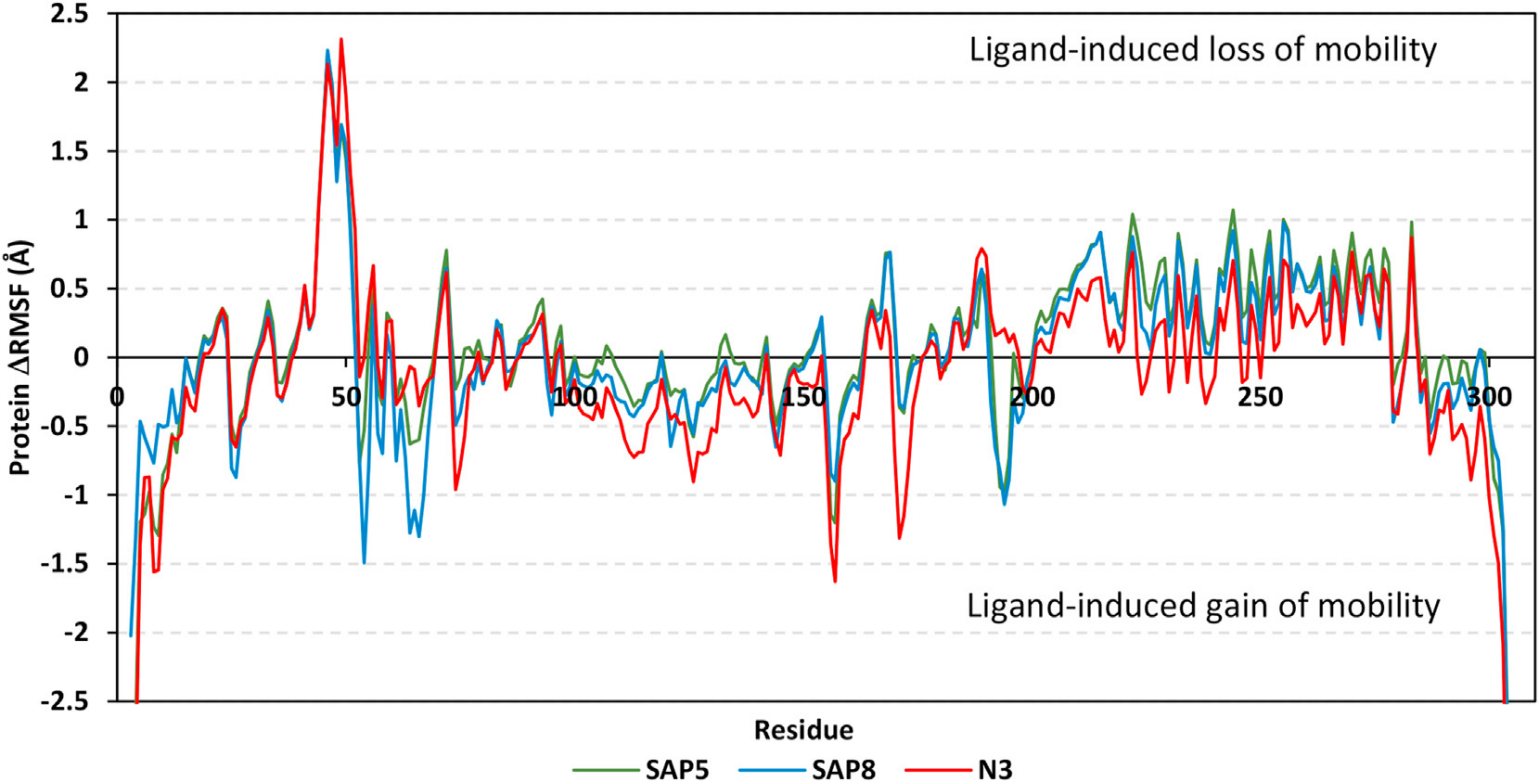
Analysis of ΔRMSF trajectories versus residue number of the three investigated ligand-protein complexes over 100 ns all-atom MD simulation. The ΔRMSF values, about protein backbone, were estimated considering independent MD simulation of SARS-CoV-2 main protease apo-state (PDB ID: 6y84) against the holo-states being complexed with either of the three investigated ligands and finally being represented as a function of residue number (residues 1-to-306). Trajectories for SAP5/protein, SAP8/protein, and N3/protein complexes are represented in green, blue, and red, respectively.

As a general observation, residues with three distinct region sequences, 41–52, 165–169/187-190, and 202–278, have shown a significant decrease within their backbone mobility up on complexing with the three ligands. It worth noting that residues within the sequence range 202–278 are more than 30 Å distance from the bounded inhibitors. The latter observation infers that the target binding site is capable of accommodating large inhibitors without impacting the site stability. Nevertheless, the ΔRMSF values within the latter distant region were slightly lower for the N3 bound protein as compared to those of the bound triterpenes suggesting lower residue stability. Regarding residues with the highest fluctuations, the terminal free residues are of the highest negative ΔRMSF values since they are most likely to fluctuate at the highest RMSFs in comparison to core residues which is a typical MD simulation behavior. However, another interesting residue range showed great mobility with Cys156 being the most fluctuating residues depicting ΔRMSF -1.14, −0.84, and −1.36 Å for SAP5, SAP8, and N3 complexed proteins, respectively. Another interesting observation is that residues with vicinity to the 41–52 and 187–190 residue ranges exhibited higher mobility and fluctuations at SAP8-bound protein compared to the other two ligands (Fig. 3). This may be rationalized for the poor ligand-protein interaction and ligand accommodation at the S1’ and S3 sub-pockets across the MD simulation run.

Extensive analysis of the obtained ΔRMSF values permits comparative analysis of the active site residue mobility across the three complexed proteins. Interestingly, only the catalytic His41, of the S1′ sub-pocket residues, exhibited decreased mobility across the three proteins, with the highest ΔRMSF value was for all N3-bound proteins (Table 3). However, the other S1′ sub-pocket including the Cys145, exhibited regained mobility despite the presence of the complexed ligands. For decreased mobility of S1 residues, only Glu166 showed limited fluctuations particularly for SAP5 triterpene, inferring the residues’ key role for ligand anchoring. Finally, several residues of the S2 and S3 sub-pockets, including Met49, Met165, Leu167, Asp187, Arg188, Gln189, and Thr190, depicted significant immobility/stability across the three proteins, yet higher values were mainly assigned for the N3-bounded protein. However, the S3 residue Leu167 was depicted of significant mobility within N3-bound protein rather than for the triterpenes (ΔRMSD = 0.07 Å versus 0.33 Å and 0.29 Å for SAP5 and SAP8, respectively). All the above ΔRMSF findings suggest the greater stability of the N3-bound complex followed by, or even comparable, the SAP5 triterpene complex and finally being minimal for the SAP8-protein complex. These came in great concordance with the obtained results of the above RMSD analysis.

**Table 3:** Estimated ΔRMSF^a^ parameter of ligands-bound proteins across the whole structure trajectories.

Sub-pocket	Residue	SAP5-protein	SAP8-protein	N3-protein
S1′	His41	0.465	0.484	0.525
	Gly143	−0.284	−0.44	−0.390
	Ser144	−0.493	−0.651	−0.585
	Cys145	−0.368	−0.451	−0.71
S1	Phe140	−0.203	−0.179	−0.392
	Leu141	−0.012	−0.268	−0.222
	Asn142	0.146	0.084	0.023
	His163	0.029	−0.063	−0.165
	Glu166	0.303	0.263	0.209
S2	Met49	1.685	1.691	2.312
	Tyr54	−0.528	−1.492	−0.003
	His164	0.289	0.24	0.206
	Asp187	0.290	0.261	0.408
	Arg188	0.217	0.526	0.716
S3	Met165	0.415	0.366	0.343
	Leu167	0.326	0.291	0.066
	Gln189	0.628	0.641	0.792
	Thr190	0.573	0.382	0.736
	Gln192	−0.483	−0.663	0.158

^a^ Relative difference root-mean-square fluctuation (ΔRMSF) was estimated for each ligand-bound protein relative to the apo state of SARS-CoV-2 main protease (PDB ID: 6y84). Only residues with significant mobility changes (ΔRMSF > 0.3 Å) are represented in bold and values are highlighted.

#### 3.3.3 Global stability analysis

Global stability of the protein ternary structures and ligands is mostly accounted to both the SASA and Rg using GROMACS “*gmx sasa*” and “*gmx gyrate*” scripts. Generally, SASA correlates for the molecular surface area being assessable to solvent molecules providing a quantitative measurement about the extent of protein/solvent interaction.^73^ Decreased SASA tones imply relative structural shrinkage for the protein/ligand under the impact of the solvent surface charges yielding more compact and stable conformations. Similarly, low Rg values imply sustained stability and compactness of the investigated molecules across the MD simulation windows. This parameter is defined as mass-weighted RMSD for a group of atoms relative to their common mass center. Thus, the structure stability, within a valid MD simulation, is correlated to Rg tones reaching a plateau around the average values.^74^

**Fig. 4:**
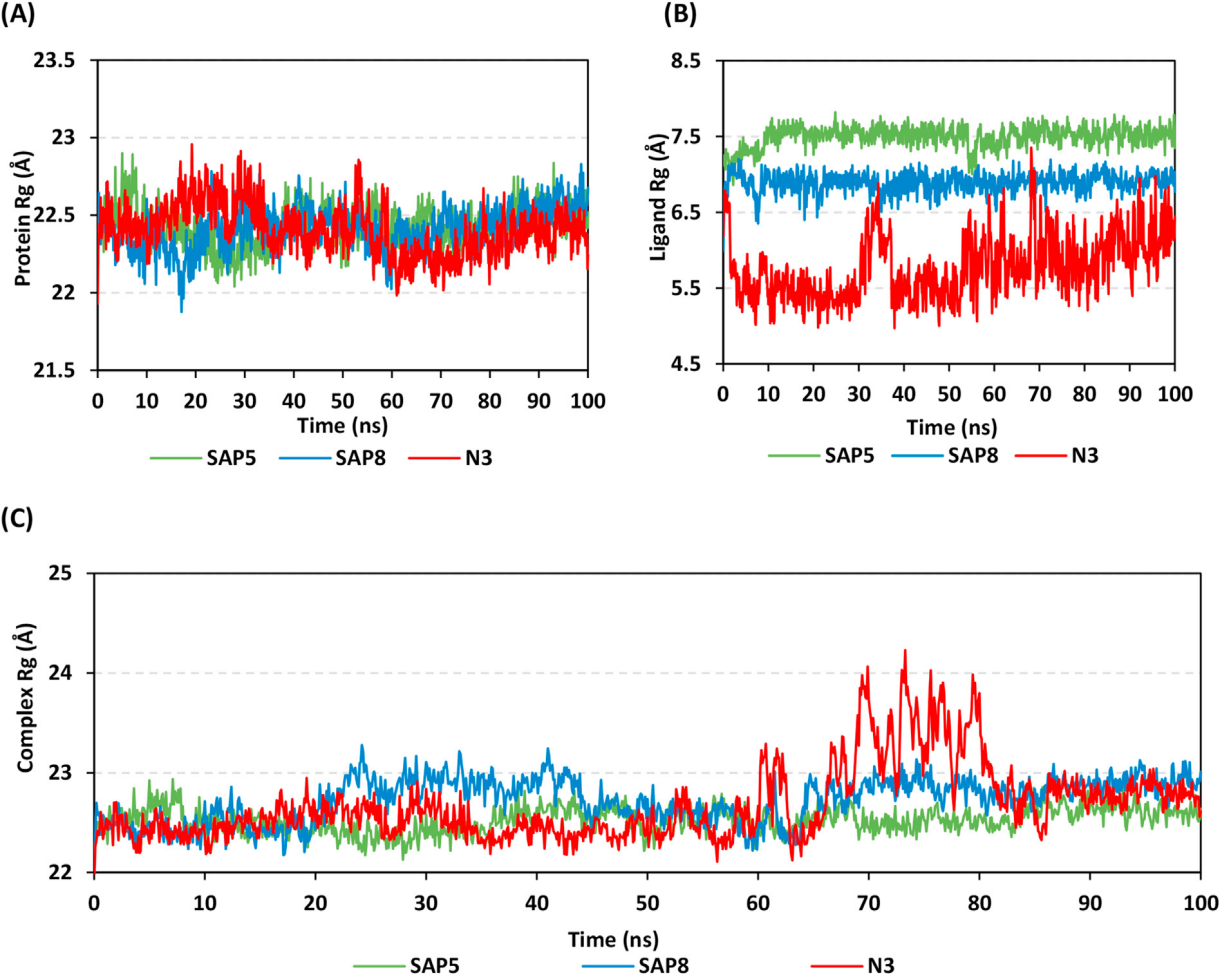
Time-evolution Rg trajectories of the three investigated ligand-protein complexes over 100 ns all-atom MD simulation. (A) protein Rg; (B) ligand Rg; (C) ligand-protein complex Rg, as a function of the MD simulation time (ns). Trajectories for SAP5/protein, SAP8/protein, and N3/protein complexes are represented in green, blue, and red, respectively.

In the presented study, findings from the Rg analysis appear to ensure the preferential stability of the SAP5-protein complex previously presented by the RMSD trajectory analysis. The protein Rg values were, to some extent, comparable among the investigated systems being fluctuated around close averages (Fig. 4A and Table 4). Nevertheless, the ligand Rg traces were significantly different where SAP5 exhibited the steadiest trajectories suggesting significant stability and compactness within the protein active pocket (Fig. 4B). The Profound Rg fluctuations were depicted for the other two ligands, particularly N3, suggesting poor accommodation within the protein pocket, particularly around the 70 ns MD simulation frames. The highest average Rg (7.50 Å) for SAP5 is just related to its largest molecular weight since Rg is directly dependent on the mass of the molecule. Finally, the presented Rg tones of the ligand-protein complexes were highly comparable to those presented with the RMSD analysis (Fig. 4C). Significant more complex compactness was assigned for the SAP5-protein complex, where steady Rg trajectories have been depicted across the 100 ns MD simulation window. This was obvious through the comparable maximum, minimum, and average values of complex Rg trajectories (22.97 Å, 22.13 Å, and 22.53 Å, respectively). Moreover, the complex Rg maximum value of the SAP5 system was significantly lower than those of the SAP8 and N3 systems the thing that further confirms preferential stability of the SAP5/protein complex.

**Table 4:** Estimated Rg parameter of top docked ligands and crystallized ligand complexed with SARS-CoV-2 main protease across all-atom MD simulation.

	SAP5-protein complex			SAP8-protein complex			N3-protein complex			
Reference atom group	Max. (Å)	Min. (Å)	Average (Å)	Max. (Å)	Min. (Å)	Average (Å)	Max. (Å)	Min. (Å)	Average (Å)	
Protein	22.90	22.04	22.43	22.83	21.88	22.42	23.00	21.93	22.40	
Ligand	7.82	6.86	7.50	7.20	5.99	6.89	7.35	4.97	5.77	
Complex	22.97	22.13	22.53	23.28	22.16	22.72	24.23	21.90	22.68	

**Fig. 5:**
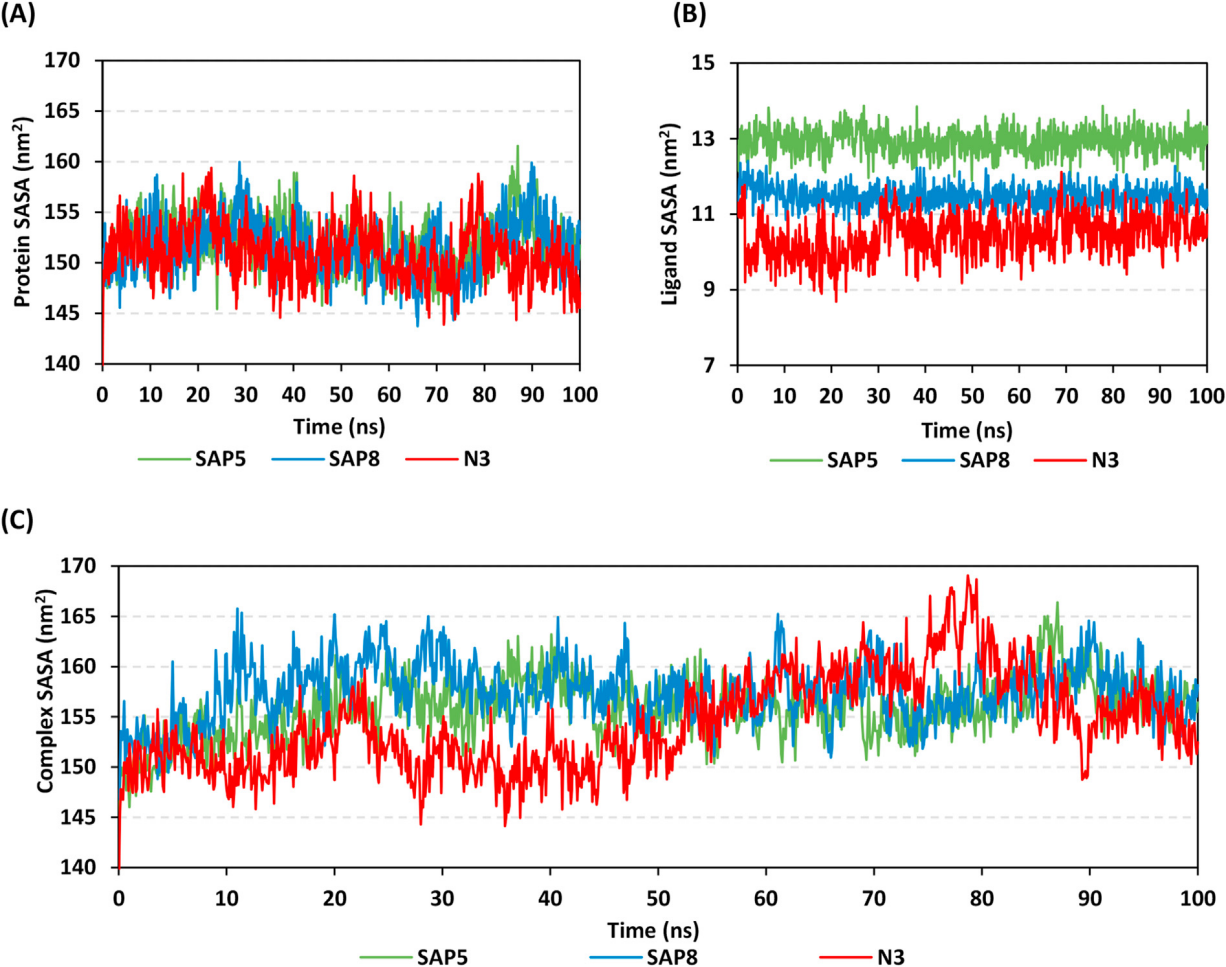
Time-evolution SASA trajectories of the three investigated ligand-protein complexes over 100 ns all-atom MD simulation. (A) protein SASA; (B) ligand SASA; (C) ligand-protein complex, as a function of the MD simulation time (ns). Trajectories for SAP5/protein, SAP8/protein, and N3/protein complexes are represented in green, blue, and red, respectively.

Moving towards the other global stability parameter, the traces of protein SASA values fluctuated around a constant average value 151.99, 151.51, and 150.93 nm^2^ for SAP5-, SAP8-, and N3-bounded proteins, respectively. The latter demonstrates the stability of solvent-exposed areas (both hydrophilic and hydrophobic) confirming the validity of the 100-ns simulation frame for obtaining the equilibrated systems. The three protein systems exhibit comparable behavior where around 55 ns a decrease in SASA tones was observed suggesting a slight disruption within important intra-protein hydrogen bond interactions (Fig. 5A). Concerning the ligand SASA trajectories, both ligand SAP5 and SAP8 showed higher SASA values with minimal fluctuations as compared to those of N3 (Fig. 5B). The latter findings can be correlated to the great polar architecture of the investigated triterpenes-based compounds as well as molecular size. Possessing higher molecular mass as having four sugar moieties, SAP5 became highly solvated, the thing that has been correlated to the ligand's high SASA values (12.93 nm^2^). The other triterpene, SAP8, showed lower average SASA trajectories, 11.46 nm^2^ since the ligand possesses a lower molecular mass having only three sugar moieties.

Investigating the impact of the ligand solvation on the ligand-protein complex solvation behavior, the SASA trajectories of the three investigated complexes were monitored (Fig. 5C). As predicted, the SASA tones of both triterpene-protein complexes were comparable, yet with a little bit lower average SASA values for the SAP5 binary complex (155.93 nm^2^ versus 157.47 nm^2^), particularly within the first 15 ns. Such dynamic behavior suggests preferential confinement of SAP5 ligand within the protein pocket. On the other hand, the N3-protein complex showed significantly lower SASA trajectories (average 151.00 nm^2^) till reaching 50 ns of simulation run suggesting better ligand-protein interactions since the latter is a solvent-substitution process. After this stage, the SASA tones gradually increased reaching max SASA values at which was followed by a subsequent decrease till reaching the end of the MD simulation run. The elevated SASA trajectories might confer the migration of N3 towards the solvent side within the simulation time frames 60-to-80 ns where the protein pocket became highly solvated and minimally compacted. Based on all furnished results from the RMSD, RMSF, SASA, and Rg analysis, differential dynamic behavior has been depicted for each investigated ligand across the designated MD simulation course. Therefore, investigating the time evolution of ligand-protein binding interactions as well as the differential complex conformation across selected trajectories would provide the appropriate bases for understanding the true ligand-protein complex stability.

#### 3.3.4 Binding interaction analysis

**Fig. 6:**
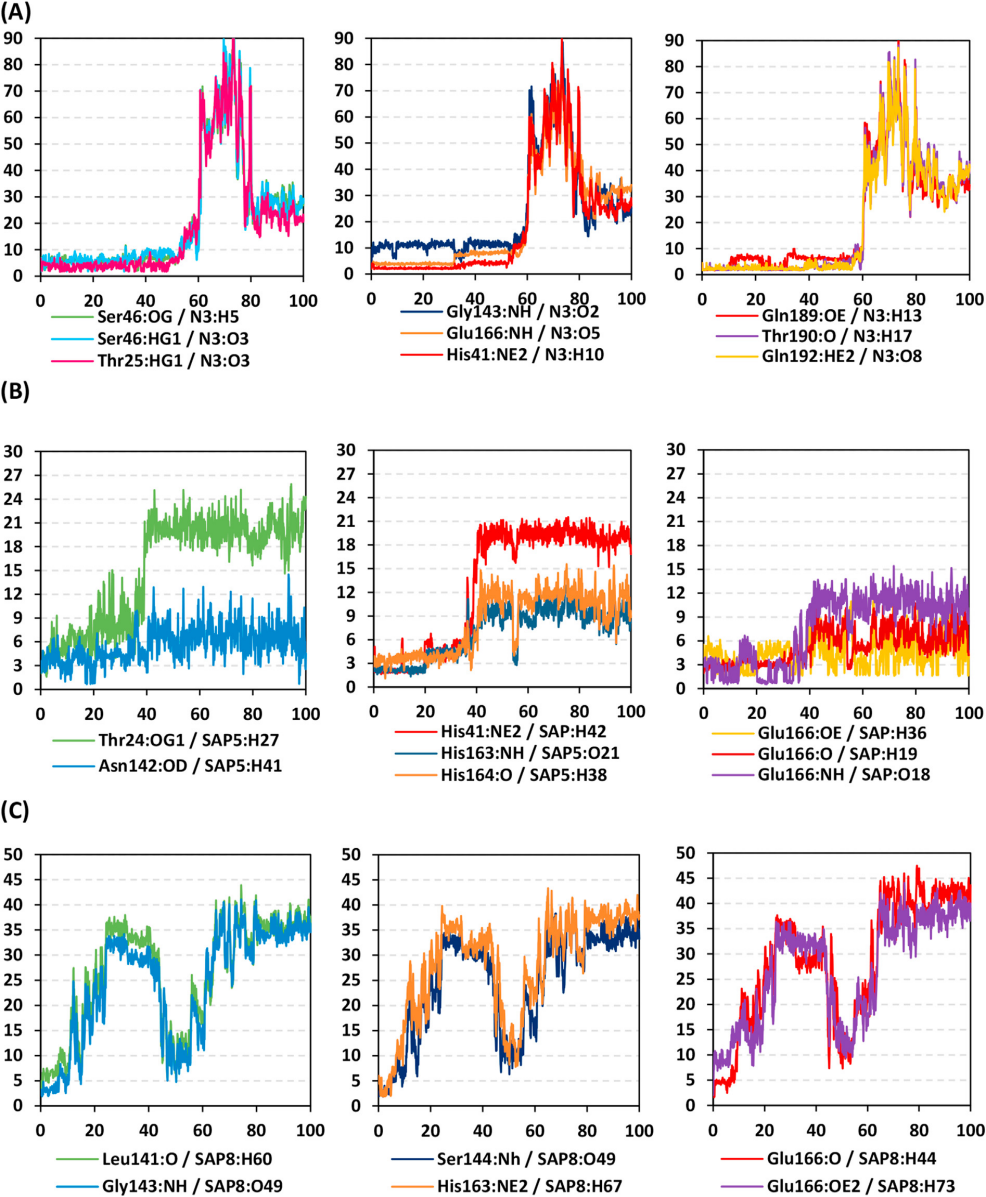
Time-evolution hydrogen donner-acceptor distances between the two final promising leads and control inhibitor with crucial protein pocket residues versus 100-ns MD simulation time. (A) N3 control inhibitor; (B) SAP5; (C) SAP8. The vertical and horizontal axes represent the hydrogen donner-acceptor distances (Å) and MD simulation time (ns), respectively.

The stability of the hydrogen bond network interactions between ligand and protein crucial residues were investigated across the MD simulation. The depicted time-dependent variations within the hydrogen donor-acceptor (HD-A) distances were illustrated through the distance trajectories in Fig. 6. Notably, the N3-protein complex showed great conformational and orientational shifts across the MD simulation window. Within the time frame from 0-to-60 ns, the N3-protein residues depicted maximum distances around 12 Å. Beyond this time frame, a rapid increase in HD-A paired distances was demonstrated with an average of ∼55–∼60 Å (Fig. 6A). The latter indicated that N3 was shifted far away from the protein pocket towards the solvent side the thing that was in great agreement with findings from the obtained RMSD, Rg, and SASA analysis. This straying away ligand has proceeded until the 80 ns of the MD simulation frame where almost consistent distances were depicted till the end of MD simulation at 100 ns. The latter behavior correlates to the returning of N3 for adopting new conformation and orientation with its respective protein. Therefore, the stability of the initial N3-protein binding mode is only confined within the first 60 ns of MD simulation where the loss of ligand-pocket accommodation was depicted as the simulation progress. Within this 60-ns time frame, relevant hydrogen bond interactions were depicted as crucial for stabilizing the N3 within the protein active pocket. Being within the optimum H-bonds distance range (2.3–3.0 Å), the hydrogen bonding pairs His41 sidechain NE2/H10 (38% occurrence), Thr190 mainchain O/H17 (23%), and Gln192 mainchain NH/O2 (40%) showed the steadiest trajectories, around 2.5–3.1 Å, throughout the time frame 0-to-60 ns. Notably, Glu166 mainchain NH/O5 (9%) HD-A pair showed steady trajectories only from 0 to 35 ns where the distance increases up to ∼9 Å till 60 ns. On the other hand, higher fluctuations with either discontinuous hydrogen bonds or long average distances (∼6–∼10 Å) was illustrated for the HD-A pairs by Ser46 (3%), Thr25 (4%), and Gly143 (1%) indicating their inferior role in N3-protein complex stability across 0–60 ns. For Gln189 hydrogen pair (12%) the interaction was interrupting across the 0–60 ns run. It worth mentioning that, maximum protein and ligand Rg values were assigned at around 53 ns which implies the loss of N3-protein complex compactness concerning the weak or even loss of ligand-protein hydrogen bond interaction.

Moving towards the HD-A distance analysis for SAP5, interesting findings were depicted. Up to five hydrogen bond pairs were depicted as stable (∼1.9–∼3 Å) across the MD simulation time frame from 0 to 40 ns. The S1 residue Thr24 showed transient hydrogen bonding (7%) with the ligand's terminal sugar part during MD simulation (Fig. 6B). On the other hand, the hydrogen bond analysis implied important role of His41 sidechain NE2/H42 (21%), Asn142 mainchain O/H41 (15%), His163 sidechain NH/O21 (20%), His164 mainchain O/H38 (13%), and Glu166 mainchain O/H19 (23%) and mainchain/O18 (17%) within the stability of SAP5-protein complex. For almost all HD-A distances, an apparent increase was depicted beyond the 40 ns simulation window reaching an average distance from ∼6 to ∼10 Å. Steadiest trajectories were assigned for His41 sidechain NE2/H42, His163 sidechain NH/O21, and Glu166 mainchain O/H19 HD-A pairs within the first 20 ns. Only Glu166 sidechain OE/H36 hydrogen bond pair (48%) was considered optimum through interrupted MD simulation intervals beyond 40 ns. Exhibiting the highest occurrence and longer time existence (beyond 40 ns), the HD-A distance analysis for Glu166 main and sidechain infer the residue's crucial for stabilizing SAP5 at M^pro^ pocket. Notably, Asn142 mainchain O/H41 showed the least increase highlighting the residue's significant role for SAP5 affinity towards the protein site. Beyond the 40 ns time frame, the HD-A trajectories showed minimal fluctuations across an average constant. This behavior is consistent with a little shift of the SAP5 within the protein site rather than being strayed far towards the solvent side.

Concerning the last ligand system, SAP8-protein complex, transit HD-A distances with significant residues was seen at the first 3 ns of the MD simulation run which soon lost as the ligand maintained an average HD-A distance of ∼30 Å within the time interval between 24-to-44 ns as well as 35 Å from 65 ns till the end of MD simulation at 100 ns (Fig. 6C). Occupancies of the ligand/residue hydrogen bond pair were of minimal percentages; Leu141 (0.8%), Gly143 (2%), Ser144 (2%), His163 (0.9%), and Glu166 (4%). The latter dynamic behavior implying poor affinity towards the protein site and minimal impact of the hydrogen bond interaction at the initial docking structure to confine SAP8 within the target active site.

#### 3.3.5 Conformational analysis across selected trajectories

**Fig. 7:**
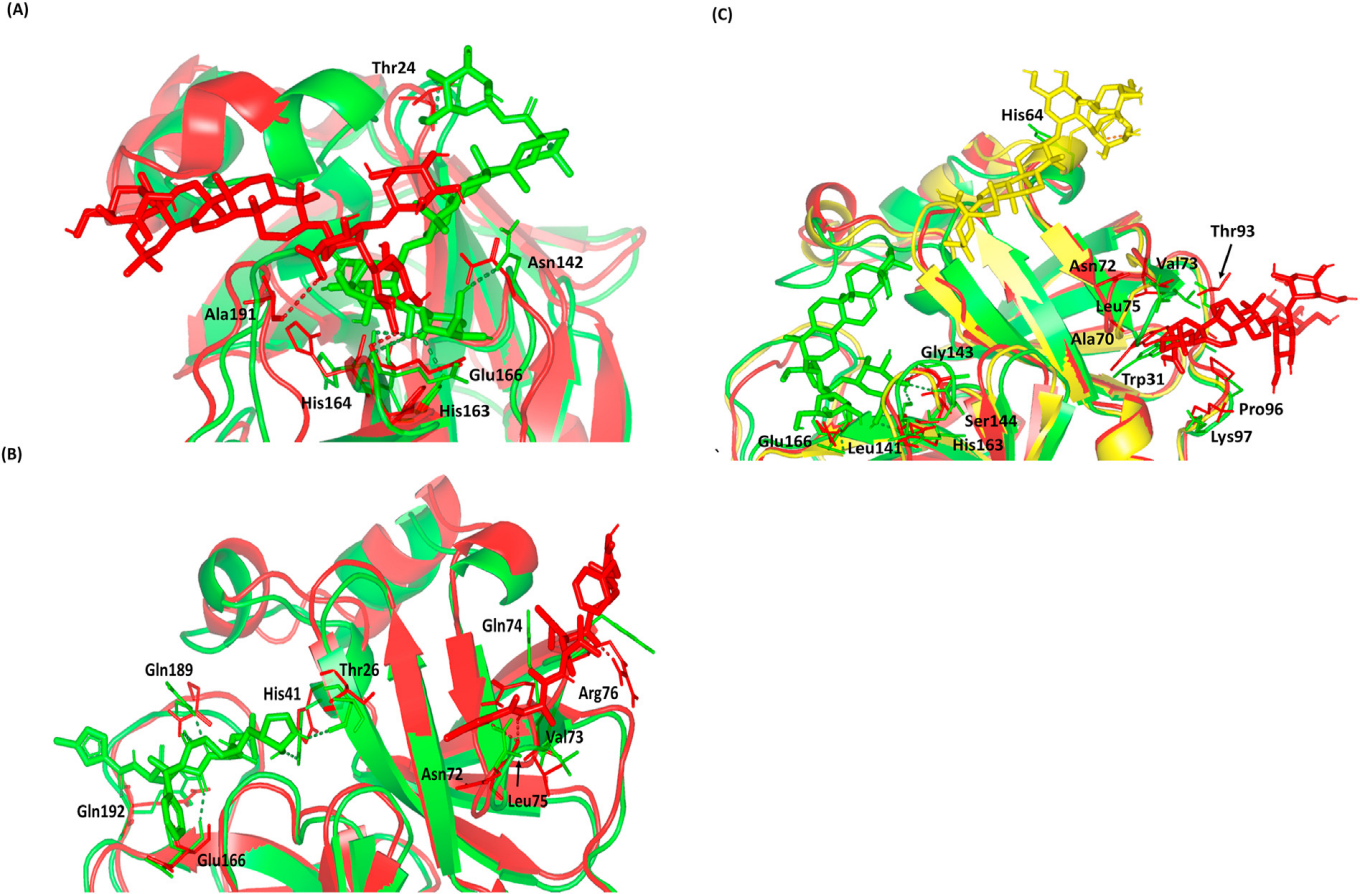
Conformation analysis of ligand-protein complex within the binding site of SARS-CoV-2 M^pro^ at selected frames. (A) SAP5; (B) N3 potent inhibitor control; (C) SAP8. Protein is represented in cartoon 3D representation and colored in green, yellow, or red as corresponding to the extracted frames at 0 ns, 30 ns, and 100 ns, respectively. Ligands (sticks), key binding residues (lines), and hydrogen bonding (dashed lines) are represented in colors corresponding to their respective time frame.

For gaining more insights regarding the newly adopted ligand-protein conformations by each ligand within the late MD simulation runs, the selected frames of each system were extracted and minimized to a gradient of 0.001 kcal/mol/A^2^ using MOE software. Fig. 7A illustrates the comparative conformations for the SAP5-protein complex at 0 and 100 ns. The initial hydrogen bond between Thr24 and the 17*β*-glucosyl moiety was lost following stabilization causing a shift of triterpene skeleton from the initial S1′ subsite towards the pocket marginal side. The higher protein RMSD-Cα before the 40 ns MD simulation window might have deeply impacted the SAP5 orientation. The greatest protein deviation was for domain-I (residues 8–101) secondary structure comprising one of the substrate's binding clefts. This domain-I dramatic conformational change deeply impacted the Thr24:OG1/H27 HD-A pair causing its loss. The latter explanation can be reasoned since almost all initial hydrogen bonding, except with Thr24, was less impacted beyond the 40 ns MD simulation frame. On the other hand, hydrogen binding between the 3*α*-glycosyl II moiety and both Glu166 and Asn142 were maintained at the end of MD simulation (100 ns) confirming the reported importance of Asn142 and Glu166 for ligand-M^pro^ protease binding.^30^^,^^61^ This picture was also confirmed through the previous binding interaction analysis (Fig. 6B). It worth mentioning that the minimal fluctuations of protein RMSD-Cα beyond 40 ns and till the MD simulation end suggested further complex stabilization with newly formed hydrogen bonding. Two hydrogen bonds were newly formed, till the end of MD simulation, for 3*α*-glucuronyl I moiety with both His164 sidechain and Ala191 mainchain. Despite the loss of the initial His164 mainchain-O/H38 hydrogen bond pair, the His164 residue depicts favored rotation of its sidechain towards the ligand 3*α*-glucuronyl I moiety allowing significant interaction.

Concerning the N3-protein complex, great conformational deviations were depicted for the secondary structures of the domain-I binding site (Fig. 7B). These deviations could be correlated to the great complex RMSD-Cα fluctuations (Fig. 1C) and the high maximum value of complex Rg (24.23 Å) compared to the SAP5-protein complex system. Therefore, domain-I instability could deeply impact the different hydrogen bond pairs between the ligand and protein, particularly for the residues within the 0–101 range. Interestingly, there is a great comparative deviation for Thr26 and His41 residues between the 0 and 100 ns frames, where the respective sidechain underwent almost a 2.3 Å and 5.5 Å shift relative to its initial residue coordination. The His41 deviation was massive as compared to other key protein residues involved in N3-protein binding interactions. The Glu166 and Gln192 conformations at the MD simulation end were of the least alterations in relative to its initial coordinates at zero frame. This was also evident across the 0-to-60 ns MD simulation window where the Gln192:NH/O2 hydrogen bond pair showed the tightest and stabilized interactions across the HD-A distance trajectories as compared to those for Thr26. Thus, great flexibility of the domain-I loop residues might allow the loss of important N3-protein interaction. At the end of MD simulation, the drifted N3 settled on the surface of domain-1 at the opposite side of the substrate-binding site. Being solvent-exposed, several polar interactions with surface hydrophilic residues, Asn72 and Arg76, permitted the stabilization of the N3 at its new site.

Moving towards the other investigated triterpene, SAP8 was bounded at proximity to the binding site depicted by N3 at the end of the MD simulation run (Fig. 7C). Nevertheless, the transition of SAP8, from the initial docked orientation to its final state, was proceeded more smoothly being depicted by the N3 ligand. SAP8 illustrated a transitional orientation at the surface cleft near the S3 pocket within the MD simulation time frame between 24-to-44 ns. Stabilization of the SAP8-protein complex within this depicted transitional orientation was mediated by a hydrogen-bond pair between the ligand's glucosyl OH group and His64 sidechain. Notably, great rotation of the His64 sidechain was observed permitting proximity towards the ligand's sugar moiety for assisted SAP8 anchoring. Final convergence into the last orientation of the ligand-protein complex depicted ligand binding at the surface cleft near that of N3. Anchoring at this last pose was guided through several hydrophobic residues including, Trp31, Ala70, Val73, Leu75, and Pro96. Nevertheless, examining several penultimate frames showed the minimal contribution of polar interactions for the SAP8 stability within its final orientation.

Comparative binding interaction analysis between the SAP5, SAP8, and N3 systems across MD simulation trajectories highlights the advantage of the triple sugar glycosidic scaffold within the SAP5 structure. The presence of a high number of hydrogen bond donor and acceptor pharmacophoric features within the sugar parts permitted the stabilized anchoring of SAP5 at the substrate-binding site despite the loss of polar contacts with domain-I residues. However, the longer stability of N3 within the binding pocket (up to 60 ns) as compared to SAP5 (∼40 ns) might be driven by several hydrophobic interactions of the ligand's aromatic/heterocyclic arms with important pocket residues. For explaining the SAP8 drifting far away from the substrate-binding site as compared to SAP5, both the 17*β*-glucosyl moiety and carboxylic 3*α*-galactosyl I moiety in SAP5 can provide some explanation. The absence of 17*β*-glucosyl moiety, within SAP8 structure, might have deprived SAP8 of hinging to the S1′ subsite at the domain I binding site. Additionally, lacking the carboxylic functional group within the 3*α*-galactosyl I moiety of SAP8 structure might compromise its stabilized anchoring through losing valuable interactions with S2 and S3 subsite residues, His164 and Ala191.

The stability of the two investigated triterpenes as well as N3 at the end of the 100 ns MD time was further investigated through extended simulation. The last system frame at 100 ns was extracted, minimized till 1 × 10^−3^ kcal/Å gradient, and then proceeded through an extra 50 ns all-atom MD simulation using the same parameters adopted within the initial 100 ns MD simulation run. Notably, both SAP5 and N3 showed significant stability across the additional trajectories since the RMSD tones for the two ligand-M^pro^ complexes were maintained at low values (2.495 ± 0.16 Å and 2.484 ± 0.15 Å, respectively) following convergence from 20 to 50 ns (Supplementary data; Figure SI5). Showing minimal fluctuations and achieving equilibrated behavior across the extended trajectories confirms the stability of SAP5 and N3 at the end of the 100 ns simulation being still bounded with the protein binding site. On the other hand, SAP8 trajectories exhibited great fluctuations across the additional 50 ns MD simulation the thing that coincides with the inferior stability behavior of SAP8-protein complex throughout the initial 100 ns MD simulation.

#### 3.3.6 Binding-free energy calculations

**Fig. 8:**
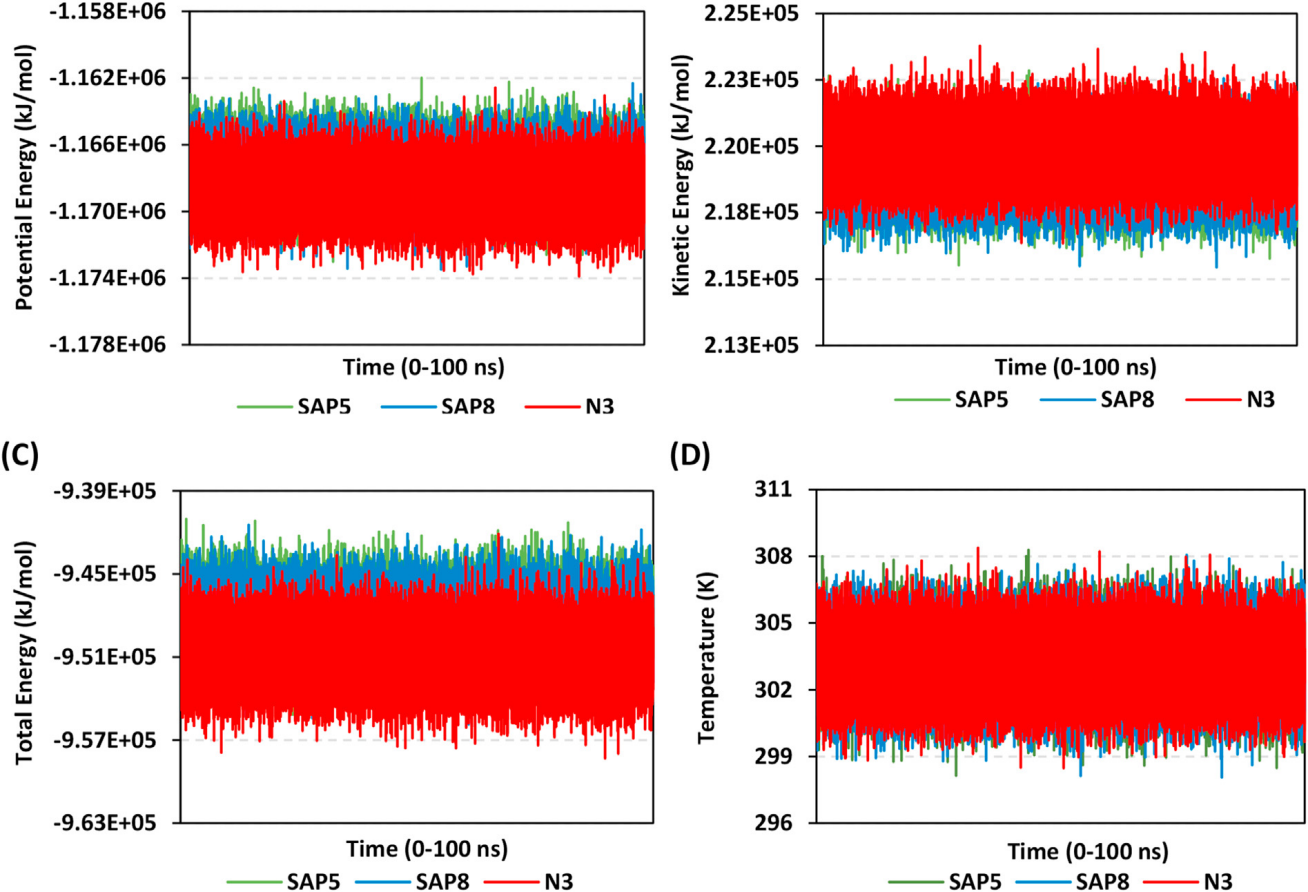
Time-evolution energy and temperature trajectories of the three investigated systems over 100 ns all-atom MD simulation. (A) potential energy; (B) kinetic energy; (C) total energy; and (D) temperature, as a function of the MD simulation time (ns). Trajectories for SAP5, SAP8, and N3-bound systems are represented in green, blue, and red, respectively.

The binding-free energy calculation was performed to understand the nature of ligand-protein interaction as well as obtain more details regarding individual ligand contribution.^75^ Thus, the MD-directed MM/PBSA approach was performed for binding-free energy calculations. Since free energy calculation is an equilibrium thermodynamic metric, examining the plots of energy (total, potential and kinetic) and temperature versus time for all systems were also beneficial to further ensure system stability across the designated 100-ns time frames. The time evolution for system energies and temperature was steady across the MD simulation times within −1.18 × 10^6^ to −1.16 × 10^6^ kJ/mol, 2.17 × 10^5^ to 2.24 × 10^5^ kJ/mol, −9.60 × 10^5^ to −9.34 × 10^5^ kJ/mol, and 298–307 K for potential, kinetic and total energies as well as temperature (Fig. 8). System stability was significant for all three systems, with slightly lower negative values for N3 total energy term and comparable values for both SAP5 and SAP8 systems.

**Fig. 9:**
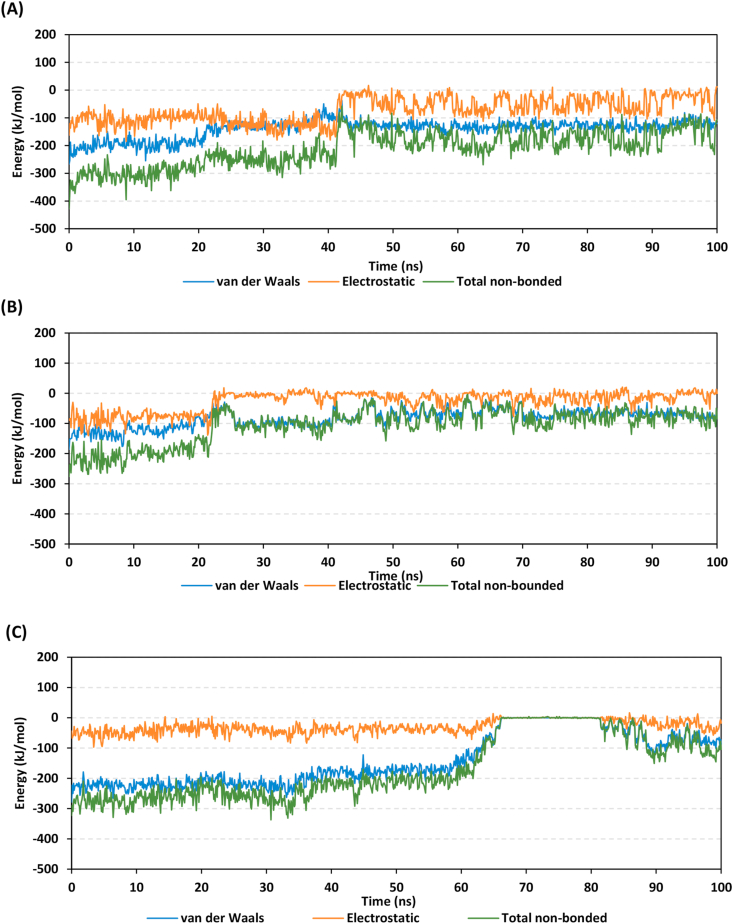
Time-evolution MD trajectories of the non-bonded potential energy and its components for the three investigated ligand-protein complexes over 100 ns all-atom MD simulation. (A) Lennard-Jones potential; (B) Coulomb's electrostatic potential; (C) total non-bonded energy, as a function of the MD simulation time (ns). Trajectories for SAP5/protein, SAP8/protein, and N3/protein complexes are represented in green, blue, and red, respectively.

In regard to MM/PBSA calculations, binding-free energy must be performed within the MD simulation window showing stabilized structure of the ligand-protein systems. Calculating the binding-free energy calculations for SAP5, N3, SAP8 systems were performed across the equilibrium intervals being guided by the complex RMSD knowledge provided earlier concerning ligand/protein stability (Fig. 1C) and conformational analysis (Section 3.3.5). The time frame intervals between 10-20 ns and 30–60 ns were considered relevant for N3. Previous analysis of the SAP5-protein complex revealed a second stabilized conformation at the end of the MD simulation window, thus binding-free energy calculation was performed on 45–100 ns as well as the initial 10–40 ns intervals. Finally, SAP8 was challenging since four equilibrium intervals were identified at 10–20 ns, 30–40 ns, 50–60 ns, and 70–100 ns. Choosing these designated intervals was also validated through monitoring the total non-bonded energies and their components (Coulomb's electrostatic and Lennard-Jones van der Waals interactions) for the three investigated ligand/protein complexes across the whole simulation times. Both the non-bonded Lennard Jones and Coulombic electrostatic potentials were calculated providing insight into the kind of ligand-protein interactions. The Lennard-Jones potential describes the potential energy of interaction between two non-bonding atoms or molecules based on their distance of separation. This is useful accounting for the Pauli repulsion and hydrophobic/van der Waals attractions.^76^ On the other hand, the Coulombic potential can describe the electrostatic interactions between atomic (partial) charges.^77^ Energy trajectories within Fig. 9 illustrated two significant energy plateaux for both SAP5- and N3-M^pro^ complexes at 10–40/50-100 ns and 10–30/35-60 ns, respectively. Additionally, potential equilibrated energy tones were depicted for the SAP8-M^pro^ complex across the 100 ns time frame including 10–20 ns, 25–40 ns, and 70–100 ns. All above equilibrium time intervals came in good agreements with those obtained from ligand/protein conformational stability states and RMSD trajectories.

Within the initial equilibration simulation windows, the free-binding energy (Δ*E*_binding_) of N3 complex was depicted one fourth-fold higher than SAP5, while being at much greater extent superior over that of SAP8 complex (−91.70 ± 19.98, −72.14 ± 38.78, and −6.35 ± 46.55 kJ/mol, respectively) (Table 5). Notably, trivial binding free energies have been depicted for the SAP8/M^pro^ system across the following equilibrium intervals where positive values have been assigned for each MD simulation window. The latter came in good agreement with the SAP8 respective complex RMSD and hydrogen bond interaction analyses, where beyond the first MD simulation frames the ligand/protein complex significantly lost its stability and relevant interactions. Concerning both N3 and SAP5 systems, highly comparable binding free energies were depicted on the subsequent equilibrium intervals (−49.77 ± 31.82 kJ/mol at 30–60 ns and −44.10 ± 61.80 kJ/mol at 45–100 ns for N3 and SAP5, respectively). It worth noting that the lower Δ*E*_binding_ at 45–100 ns highlights the significant role of SAP5 17*β*-glucosyl moiety since the loss of its respective hydrogen bonding with Thr24 significantly decreases the ligand binding free energy. Over the extended 50 ns MD time frame, both SAP5 and N3 exhibited significant free-binding energy with similar energy profiles and contributions to earlier calculations near the end of the first runs (Supplementary data; Table SI1). The latter further confirms the earlier equilibrium of the ligand-M^pro^ complexes. Considering the reported significant inhibition activity of the N3 ligand against SARS-CoV-2 M^pro30^, the obtained data within the presented study highlights the potential activity of SAP5 against M^pro^.

**Table 5:** Binding-free energies and each contributing energy term (±standard deviation; SD) for the investigated ligand-M^pro^ systems.

Energy terms (kJ/mol ± SD)	N3		SAP5		SAP8			
	10–20 ns	30–60 ns	10–40 ns	45–100 ns	10–20 ns	30–40 ns	50–60 ns	70–100 ns
Δ*E*_Van der Waal_	−223.49 ± 16.13	−191.55 ± 25.47	−191.92 ± 38.46	−126.29 ± 16.69	−131.91 ± 17.06	−82.94 ± 24.22	−69.16 ± 12.22	−79.99 ± 30.70
Δ*E*_Electrostatic_	−53.69 ± 16.88	−40.34 ± 14.23	−111.20 ± 27.54	−44.23 ± 28.72	−88.82 ± 26.48	−9.01 ± 15.22	−22.97 ± 17.83	−18.17 ± 23.08
Δ*E*_Solvation_; Polar	210.79 ± 25.22	205.22 ± 17.19	251.23 ± 44.24	143.33 ± 34.22	234.53 ± 50.14	217.95 ± 52.00	239.46 ± 56.74	233.36 ± 47.37
Δ*E*_Solvation_; SASA	−25.31 ± 2.11	−23.10 ± 2.59	−20.25 ± 4.26	−16.91 ± 2.04	−20.15 ± 2.11	−11.37 ± 3.62	−11.54 ± 2.11	−10.51 ± 4.16
Δ*E*_Binding_	−91.70 ± 19.98	−49.77 ± 31.82	−72.14 ± 38.78	−44.10 ± 61.80	−6.35 ± 46.55	114.64 ± 63.03	135.79 ± 49.15	124.69 ± 73.97

Dissecting the obtained binding-free energy calculations for the three complexes illustrated that van der Waal interactions highly contributing to the binding energy calculation as compared to the electrostatic term. This can rationalize the previous assumption that the longer stability of N3 within the binding pocket (up to 60 ns) as compared to SAP5 (∼40 ns) might be driven by several hydrophobic interactions of the ligand's aromatic/heterocyclic arms with important pocket residues. On the other hand, both triterpene ligands depicted higher Δ*E*_electrostatic_ contribution, with higher values for SAP5, than that depicted by N3 being significantly obvious at the initial equilibrium intervals for each respective ligand/protein system. The latter preferential Δ*E*_electrostatic_ contributions confirm the significant role of the polar sugar moieties for ligand anchoring within M^pro^ binding site. Lack of the 17*β*-glucosyl and carboxylic 3*α*-galactosyl I moieties within SAP8 may correlate with the lower Δ*E*_electrostatic_ for SAP8 as compared with SAP5 (−88.82 ± 26.48 versus −111.20 ± 27.54 kJ/mol, respectively) at respective first equilibration intervals. It worth mentioning that presence of triterpene sugar moiety could serve as a double-bladed effect on ligand-protein binding since higher Δ*E*_solvation_ was depicted with SAP5 > SAP8 > N3. Comparing the Δ*E*_binding_ between the depicted poses of SAP5 at 10–40 ns and 45–100 ns MD simulation, showed the great contribution of *E*_solvation_ which may be due to the increased solvation of 17*β*-glucosyl moiety following the loss of SAP5/Thr24 hydrogen bonding.

**Fig. 10:**
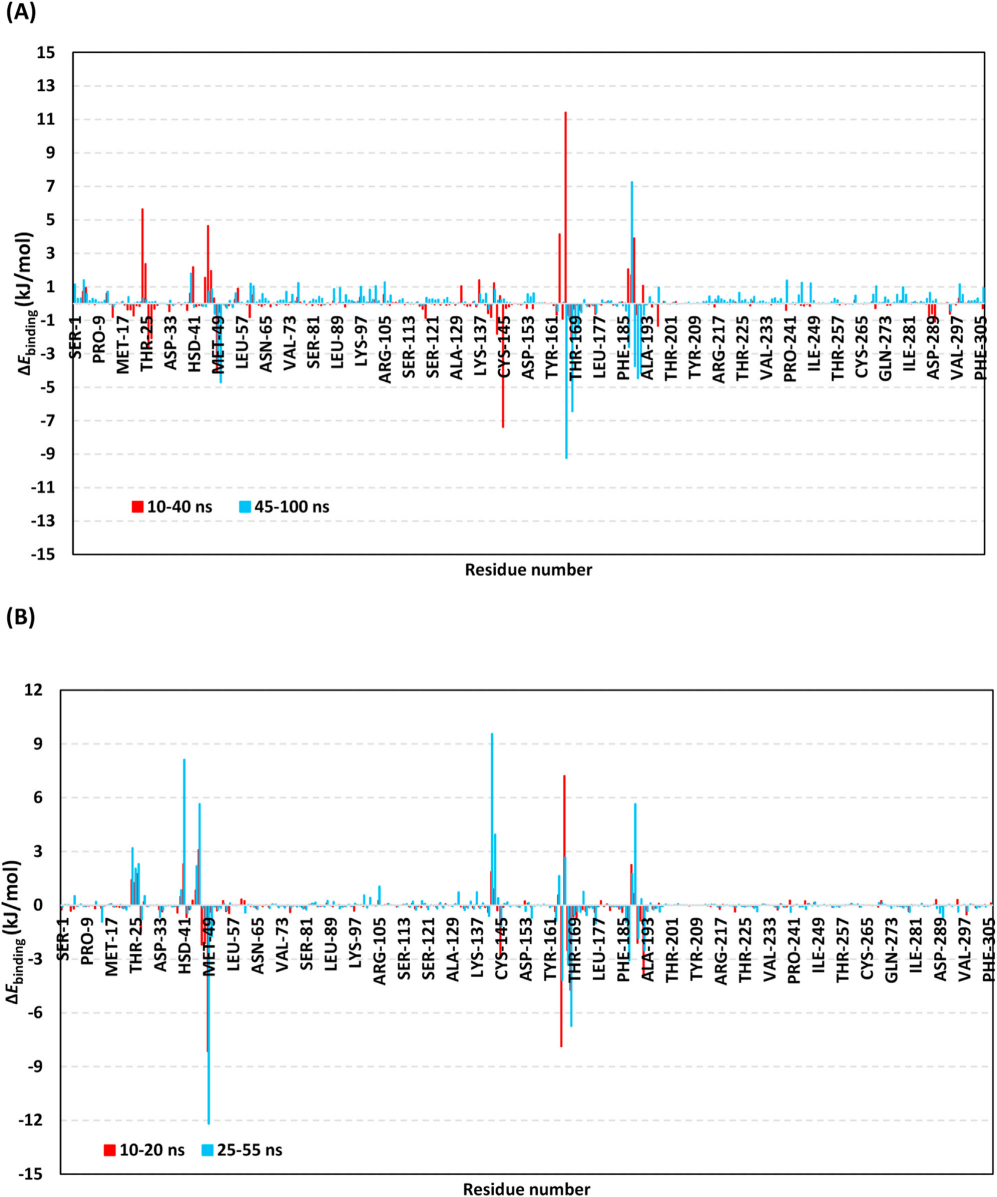
Binding-free energy/residue decomposition illustrating the protein residue contribution at ligand-protein complex Δ*E*_binding_ calculation. (A) SAP5/M^pro^ residues; (B) N3/M^pro^ residues.

For further evaluation of the comparative ligand-residues interactions at M^pro^ active binding site for SAP5 and N3, the binding-energy decomposition was used to identify key residues involved within obtained Δ*E*_binding_ (Fig. 10).^51^ At 10–40 ns time frame, several residues, which have participated within the initially docked complexes, showed significant contributions in the calculated binding-free energy. For the SAP5-protein complex, the highest residue-binding energy contribution was for S1 Glu166 confirming the key role of this residue in small molecule binding to the SARS-CoV-2 M^pro^ target (Fig. 10A). The catalytic Cys145 residue came next to Glu166 for stabilizing the ligand at the protein pocket inferring promising activity of SAP5 to interfere with the protein catalytic machinery. Other significant binding contributions were also depicted with the Met49, Gly143, His164, Met165, Asp189, Gln189, and Gln192 inferring the preferential anchoring of SAP5 at S1’, S1, and S3 binding sub-pockets. Interestingly, several vicinal residues of the M^pro^ active site including Thr24, Ser46, and Pro168 showed significant residue-wise energy contribution. The latter highlights the crucial role of Thr24-mediated hydrogen bonding for stabilizing the 17*β*-glucosyl moiety of SAP5 near the target protein active site. Moving towards the second equilibration interval (45–100 ns), several key pocket residues either maintained or further contributed within the ligand/protein binding free energy. The S1 Glu166, S1 Met49, S2 Asp187, and S3 Gln189 key residues have maintained their initial high energy contributions. On the other hand, Gly143, Cys145, and His164 showed significantly decreased energy contribution at the 45–100 ns MD simulation window. This was also similar for some pocket vicinal residues, including Thr24 the thing that came in great agreement with the previous conformational analysis. On the contrarily, vicinal residue like Leu50, Pro168, and Ala191 showed increased energy contribution at 45–100 ns as compared to their initial contributions at 10–40 ns. The latter confers their significant role in stabilizing SAP5 at this late MD simulation interval.

Moving towards the N3 complex at 10–20 ns, Met49 confined within the S2 sub-pocket showed the greatest contribution within the Δ*E*_binding_ for the N3-protein complex (Fig. 10B). Second in order, both Glu166, at S1 sub-pocket, and Met165 (S3 sub-pocket) depicted significant contribution and illustrate their significant role in N3-protein binding. For the catalytic residues, both His41 and Cys145 showed comparable contribution within N3 affinity towards the M^pro^ binding site. Several other binding site residues showed lower significance for N3 anchoring within M^pro^, including S1 Asn142, S3 Leu167, Asp187, Arg188, Thr190, and Gln192. Comparing the above 10–20 ns residue-wise contributions with that at 30–60 ns, showed that several pocket residues exhibited compromised energy contributions. These residues include the S1’ catalytic Cys145, S2 Met165, S1 Glu166, and S2 Arg188. Compromised Glu166 energy contribution in case of N3, however, is maintained with SAP5 system, highlights the superior stability of SAP5 since several studies report Glu166 being indispensable for M^pro^ binding. Additionally, the reduced Glu166 energy contribution further confirms the shift of N3 from M^pro^ main pocket for significant time frames within 30–60 ns equilibration interval. On the other hand, other protein residues showed increased energy contributions including His41, Met49, Asn142, Gly143, Pro168, and Gln189. The latter behavior implies the important role of the latter residues within N3/protein stability at 30–60 ns simulation interval.

### 3.4 Molecular properties, Lipinski rule, and ADME studies

Analyzing the pharmacokinetic properties of the tested compounds according to the BOILED-Egg revealed that all of them are not penetrating the BBB and cannot be taken orally as well. Moreover, especially compounds (10 and 11) having better oral bioavailability compared to others and are non-substrates of P-gp. On the other hand, compounds (1, 7, and 12) were predicted to be actively effluxed by P-gp. Furthermore, all other pharmacodynamic and pharmacokinetic properties of the tested compounds were depicted in the supplementary data (Figures SI6 and SI7).

### 3.5 Structure-activity relationship study

**Fig. 11:**
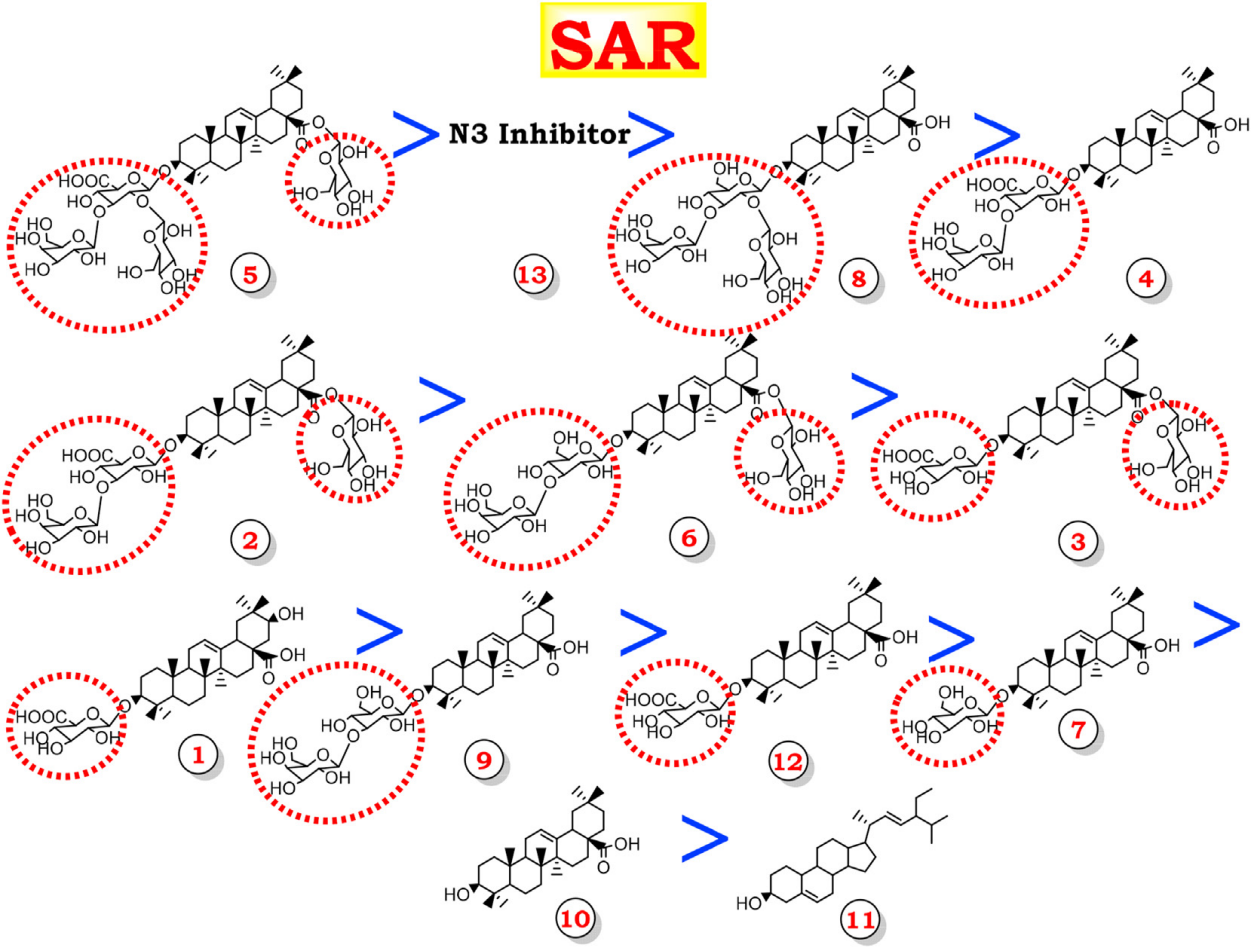
Structure-activity relationships of the tested triterpenes isolated from *C. officinalis* aerial parts.

Studying the SAR of the isolated compounds towards the SARS-CoV-2 main protease resulted in the following promising findings (Fig. 11):

At first, the presence of sugar and sugar-acid units at positions 3 of the different tested triterpenes affects greatly their binding affinities towards the SARS-CoV-2 main protease. However, the presence of an extra glucose unit at the C-28 carboxylic group of the compound (5) together with the one glucose unit at C-2′ and one galactose unit at C-3′ of glucuronic acid at position 3 gives it the superior binding affinity against the COVID-19 main protease compared to the other remaining tested eleven metabolites and surprisingly even to the co-crystallized redocked N3 (13) as well. Moreover, the presence of glucose and galactose units at C-2′ and C-3′, respectively, of the glucose unit at position 3 of the compound (8) makes it slightly lower in the main protease affinity compared to the co-crystallized N3 (13). On the other hand, compound (4) with both galactose and glucuronic acid moieties at position 3 was very close in its affinity to compound (2) with an additional glucose unit at the C-28 carboxylic acid group. Furthermore, compound (6) with glucose and galactose units at position 3 showed a very slight lower affinity against the SARS-CoV-2 main protease compared to the previously mentioned compound (2) indicating the slight activity of glucuronic acid unit compared to glucose unit at position 3. While the presence of only one glucuronic acid moiety at position 3 of compound (3) decreased its affinity towards the main protease compared to both (2) and (6) compounds. Also, the loss of glucose unit from the C-28 carboxylic acid group of compounds (1, 9, 12, and 7) decreased their protease affinity indicating the important role of it. Compound (1) with an extra OH group at position 21 and one glucuronic acid moiety at position 3 showed a better protease affinity compared to compound (9) with the glucose and galactose units at position 3. Again, the presence of glucuronic acid unit at position 3 of compound (12) gave it a slight superior affinity towards the main protease compared to compound (7) with a glucose sugar unit instead. Finally, the absence of sugar units from compounds (10 and 11) resulted in their lowest affinity towards the main protease among the tested triterpenes. Compound (10) with OH group at position 3 and the C-28 carboxylic acid group showed a slightly better affinity towards the COVID-19 main protease compared to compound (11) which lacks the carboxylic acid group.

Collectively, from the aforementioned SAR results, we can conclude the great efficacy of the triterpenoid nucleus towards the SARS-CoV-2 main protease which increases gradually by the introduction of glucuronic acid and glucose units at position 3. Moreover, the presence of glucuronic acid unit is better for the main protease affinity than glucose one at position 3. Also, the introduction of a glucose moiety at the C-28 carboxylic acid group enhances the main protease binding affinity as well.

## 4 Conclusion

Collectively, our recent detailed study confirmed the potential affinities of the isolated triterpenes and proposed their promising intrinsic activities against the SARS-CoV-2 main protease (M^pro^) as well. Moreover, both molecular docking and dynamics simulation confirmed the superior affinity of Calendulaglycoside A (SAP5) towards the SARS-CoV-2 M^pro^ compared to its co-crystallized N3 inhibitor. Accordingly, we recommend further fast preclinical and clinical studies at least for the aforementioned promising compound. Furthermore, the SAR study of the tested triterpenes may help greatly in understanding the essential requirements for the antiviral activity against the SARS-CoV-2 M^pro^ which may assist the design and synthesis of new drug candidates targeting it as well.

## Declaration of competing interest

The authors declare that there is no conflict of interest.
