# Supplementary material for: Calendulaglycoside A showing potential activity against SARS-CoV-2 main protease: Molecular docking, molecular dynamics, and SAR studies
Source: J Tradit Complement Med. 2021 May 17;12(1):16–34. doi: 10.1016/j.jtcme.2021.05.001 (PMC8126476; doi:10.1016/j.jtcme.2021.05.001)
Supplement: Multimedia component 1 [file mmc1.zip › jtcme_446_4 Supplementary Information- R2_mmc1.DOCX]

**Calendulaglycoside A Showing Potential Activity Against SARS-CoV-2 Main Protease: Molecular Docking, Molecular Dynamics, and SAR Studies**

Ahmed A. Zaki^a,b^, Ahmed Ashour^a,b^, Sameh S. Elhady^c^, Khaled M. Darwish^d^, and Ahmed A. Al‐Karmalawy^e*^

^a^ Department of Pharmacognosy, Faculty of Pharmacy, Mansoura University, Mansoura 35516, Egypt. ahmed.awad@fulbrightmail.org

^b^ Department of Pharmacognosy, Faculty of Pharmacy, Horus University-Egypt, New Damietta 34518, Egypt. [ahmedadelashour@yahoo.com](mailto:ahmedadelashour@yahoo.com)

^c^ Department of Natural Products, Faculty of Pharmacy, King Abdulaziz University, Jeddah 21589, Saudi Arabia. ssahmed@kau.edu.sa

^d^ Department of Medicinal Chemistry, Faculty of Pharmacy, Suez Canal University, Ismailia 41522, Egypt. khaled_darwish@pharm.suez.edu.eg

^e^ Department of Pharmaceutical Medicinal Chemistry, Faculty of Pharmacy, Horus University-Egypt, New Damietta 34518, Egypt. [akarmalawy@horus.edu.eg](mailto:akarmalawy@horus.edu.eg)

***Corresponding author:**

**Ahmed A. Al-Karmalawy**

Department of Pharmaceutical Medicinal Chemistry, Faculty of Pharmacy, Horus University-Egypt, New Damietta 34518, Egypt. Email: [akarmalawy@horus.edu.eg](mailto:akarmalawy@horus.edu.eg)

**Table SI1:** Binding-free energies and each contributing energy term (± standard deviation; SD) for promising investigated ligand-M^pro^ systems at the extended 50 ns all-atom MD simulation run.

| **Energy terms**  (kJ/mol ± SD) | **N3** |  | **SAP5** |
| --- | --- | --- | --- |
|  |  |  |  |
| **Δ*E*_Van der Waal_** | -213.52 ± 10.75 |  | -111.35 ± 15.87 |
| **Δ*E*_Electrostatic_** | -79.64 ± 12.21 |  | -35.64 ± 40.35 |
| **Δ*E*_Solvation_; Polar** | 240.33 ± 9.14 |  | 108.36 ± 71.38 |
| **Δ*E*_Solvation_; SASA** | -15.92 ± 0.75 |  | -13.41 ± 2.91 |
| **Δ*E*_Binding_** | -68.75 ± 14.72 |  | -52.04 ± 61.80 |

**Fig. SI1:** Chemical structures of isolated tested compounds from *C. officinalis* aerial parts.

**Fig. SI2:** 2 D diagram (a), 3 D representation (b), and protein positioning (c and d) of the superimposition of the co-crystallized (red-brown) and the docked pose (green), respectively, of N3 inhibitor inside the COVID-19 main protease binding site with RMSD of 1.23 Å.

| **(a)** | **(b)** |
| --- | --- |
| 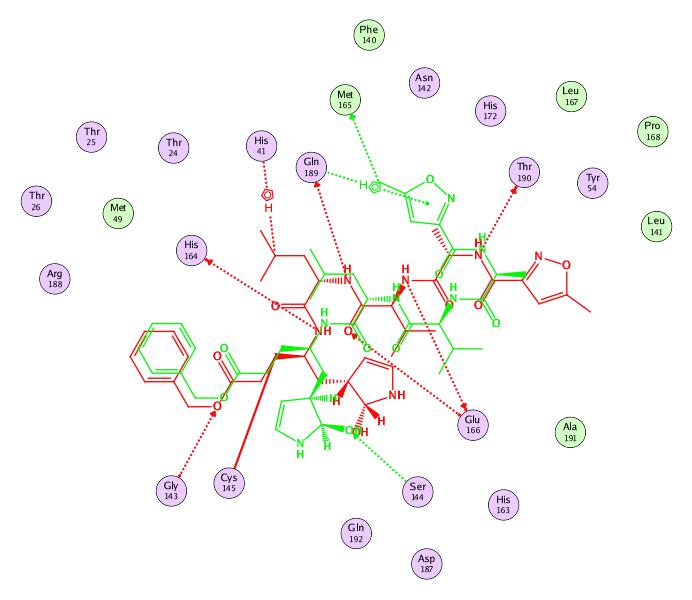 | 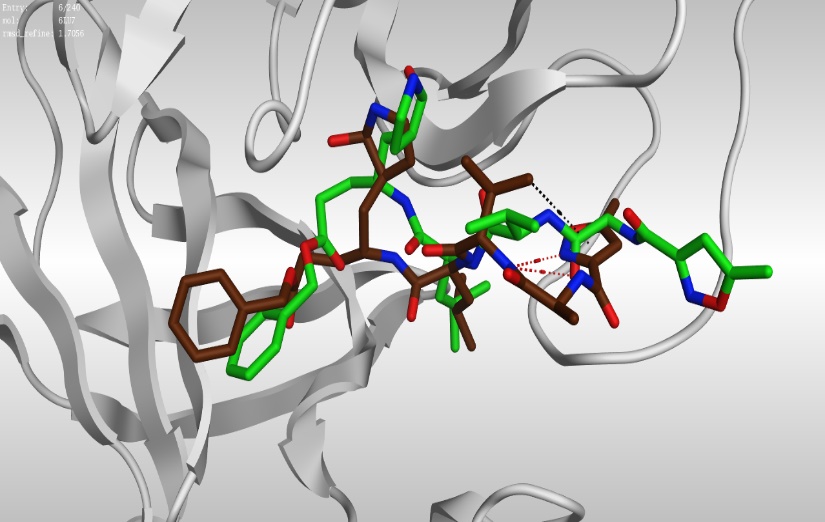 |
| **(c)** | **(d)** |
| 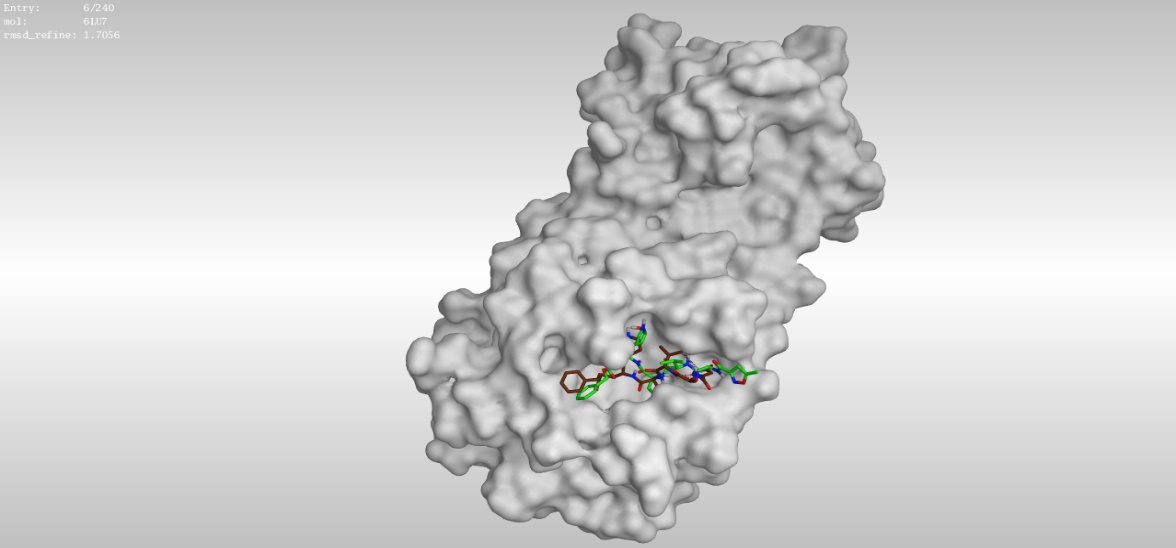 | 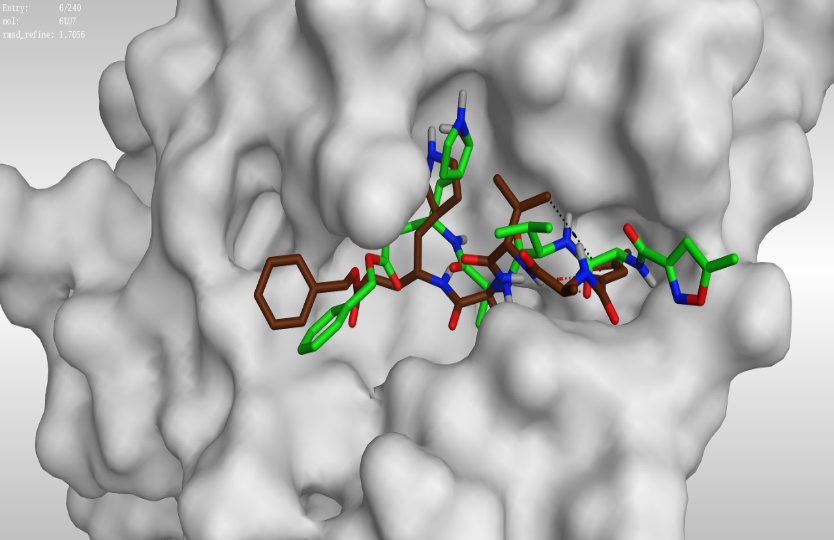 |

**Fig. SI3:**2D and 3D docking representations of the isolated tested triterpenes and N3 inhibitor against the binding site of the COVID-19 main protease.

| **No.** | **Tested compound** | **2 D** | **3 D** |
| --- | --- | --- | --- |
|  |  | 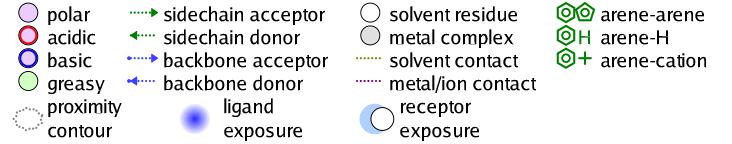 | Red dashed lines refer to hydrogen bonds, while the black ones denote hydrophobic interactions. |
| **1** |  | 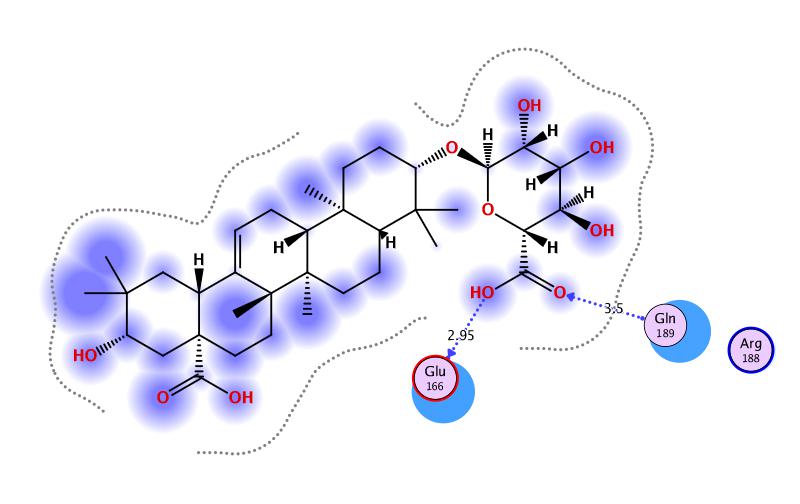 | 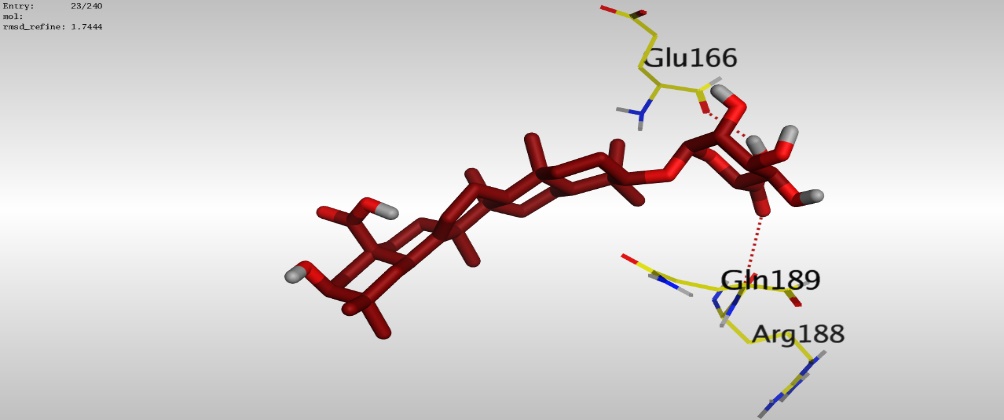 |
| **2** |  | 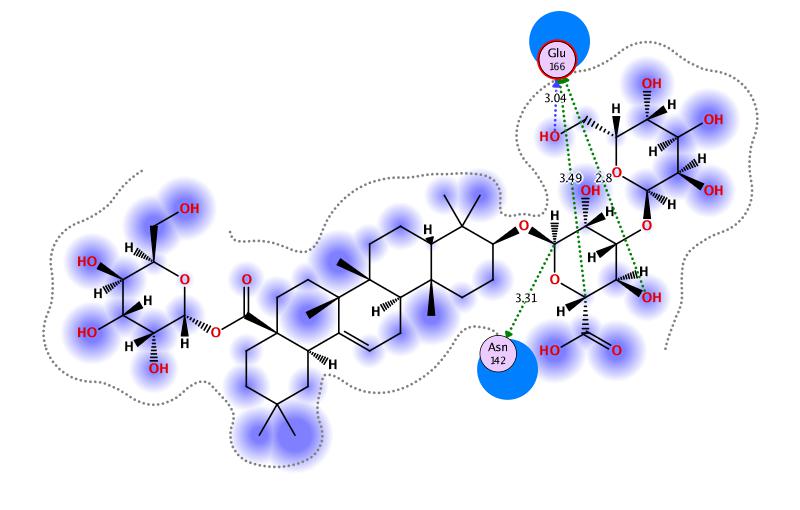 | 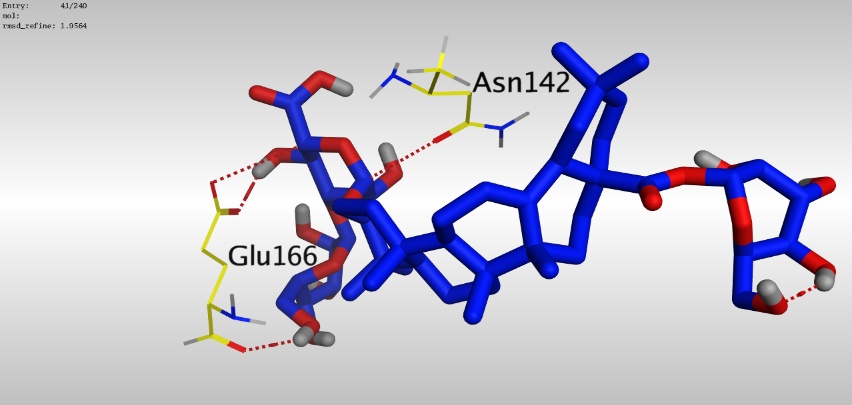 |
| **3** |  | 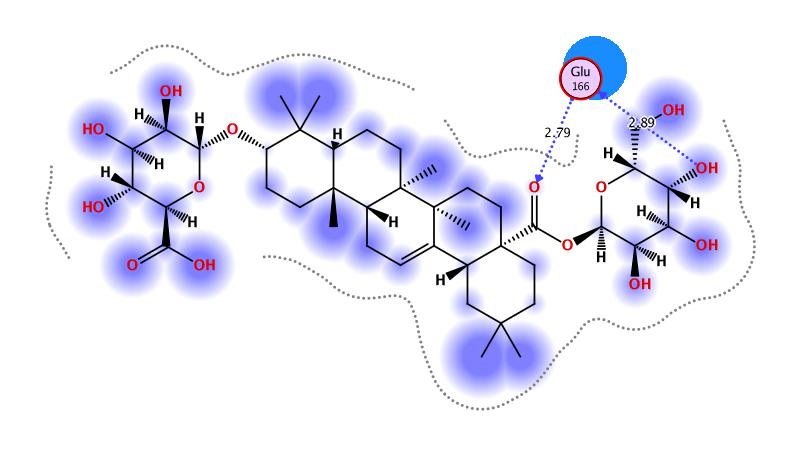 | 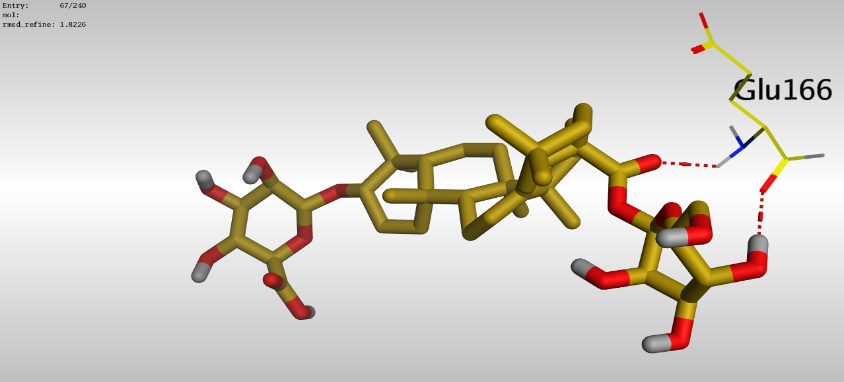 |
| **4** |  | 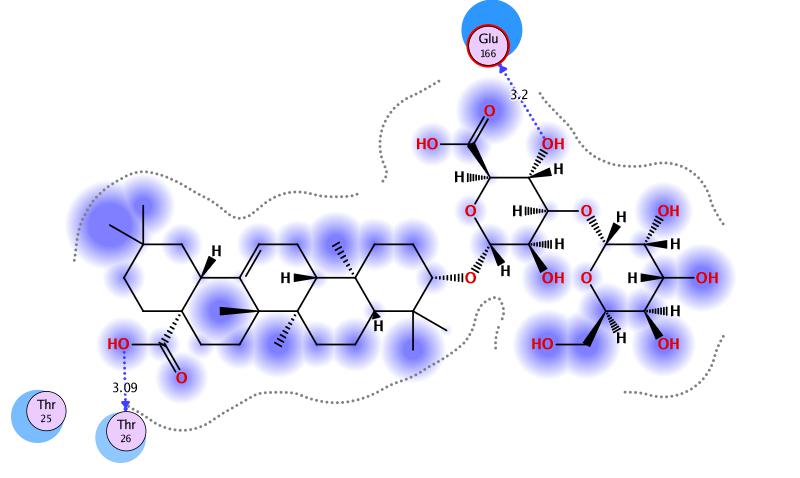 | 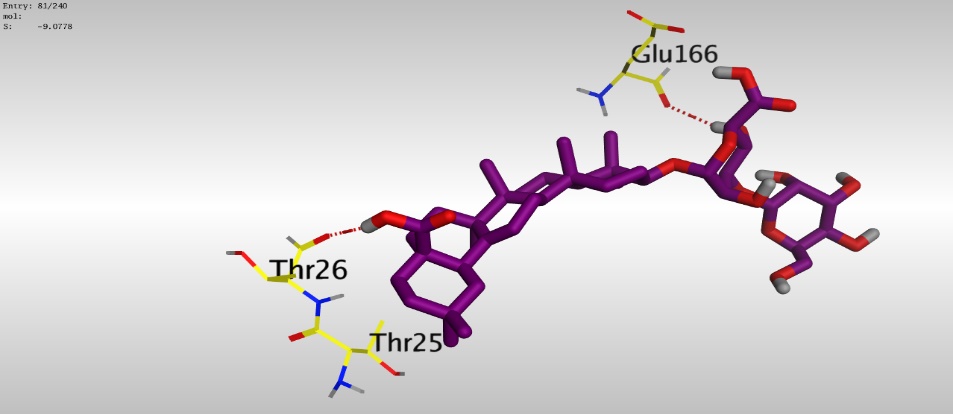 |
| **5** |  | 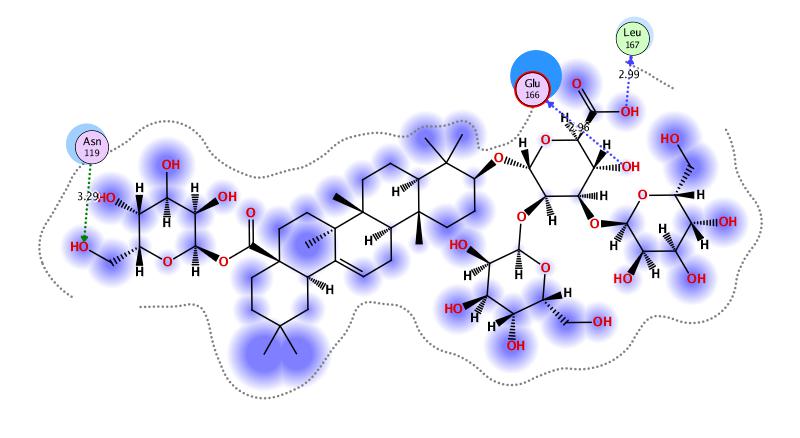 | 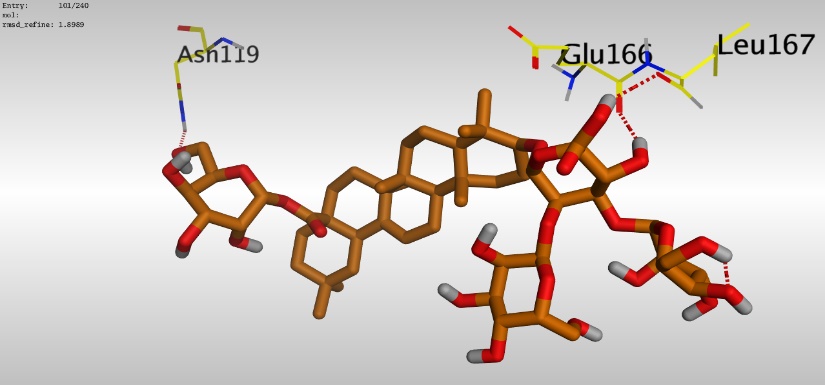 |
| **6** |  | 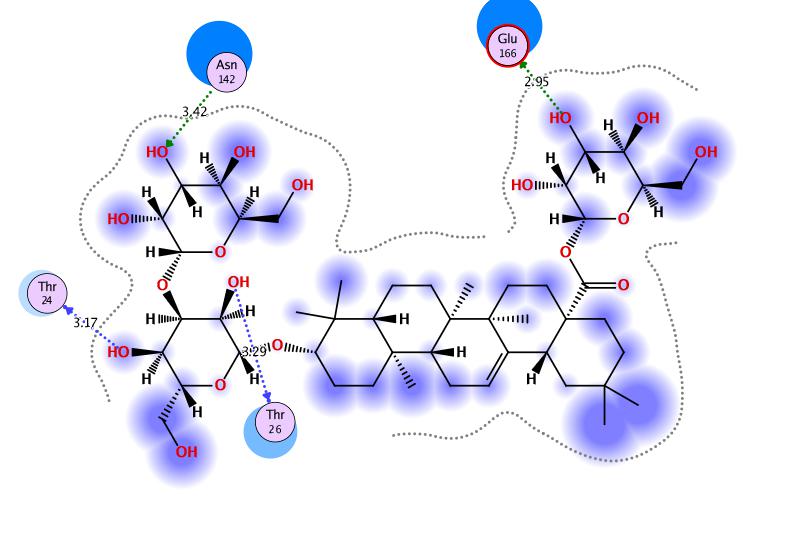 | 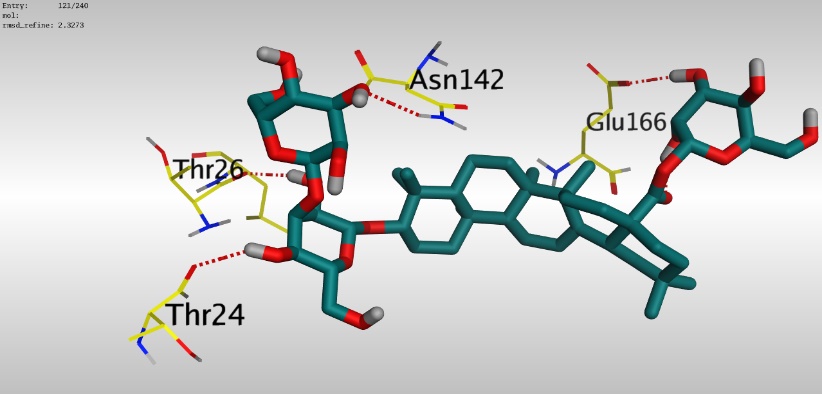 |
| **7** |  | 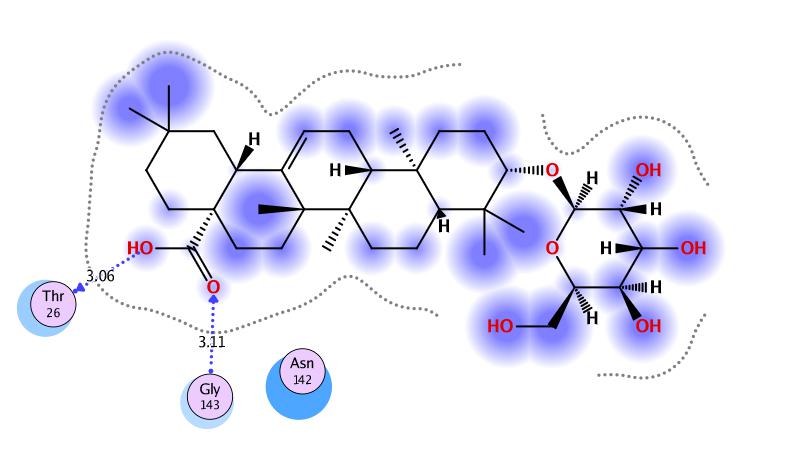 | 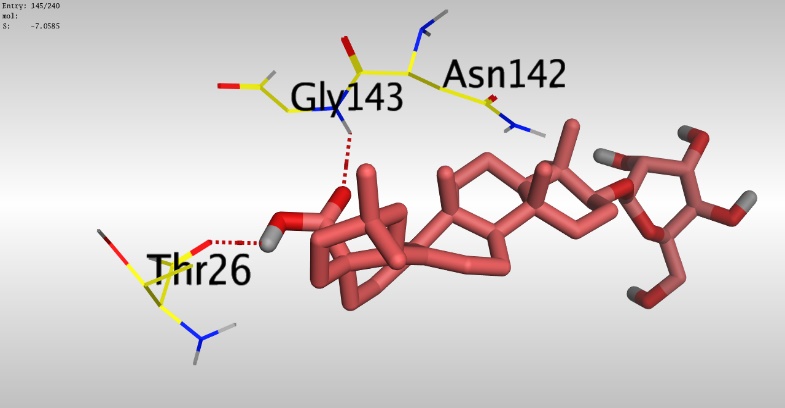 |
| **8** |  | 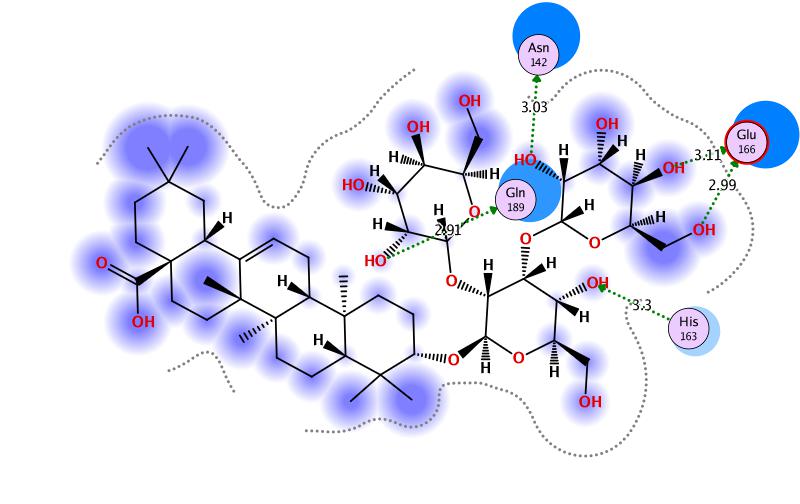 | 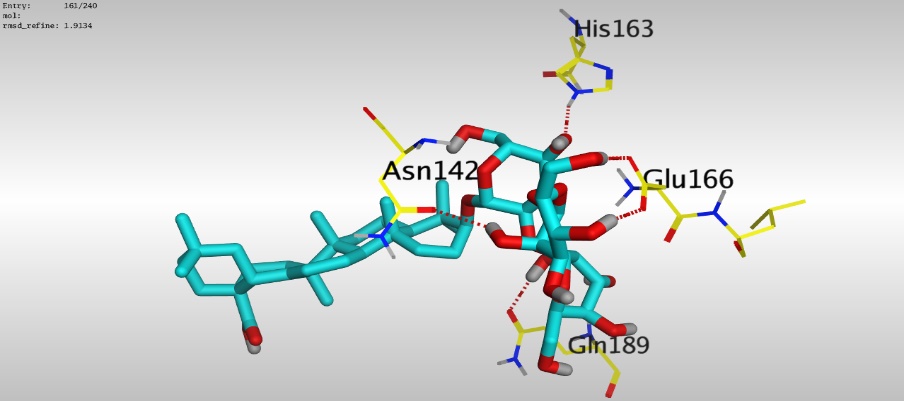 |
| **9** |  | 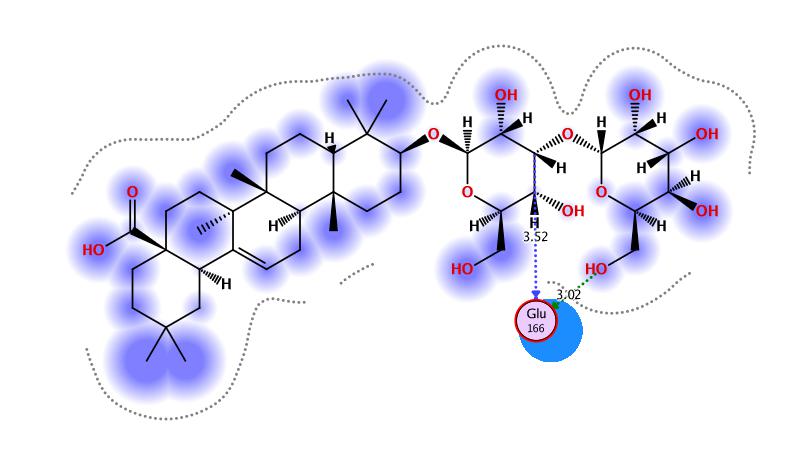 | 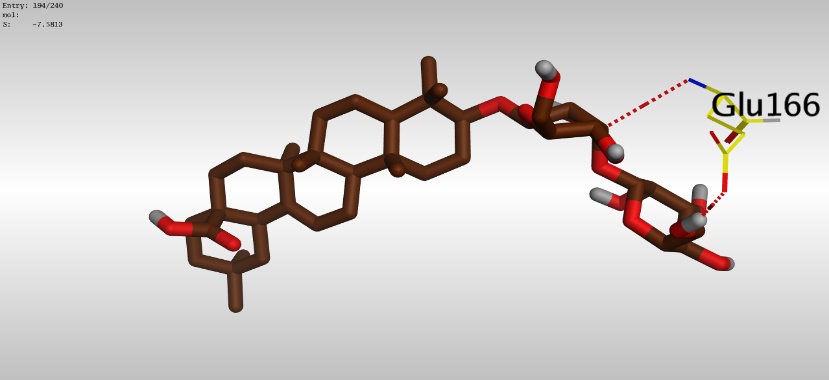 |
| **10** |  | 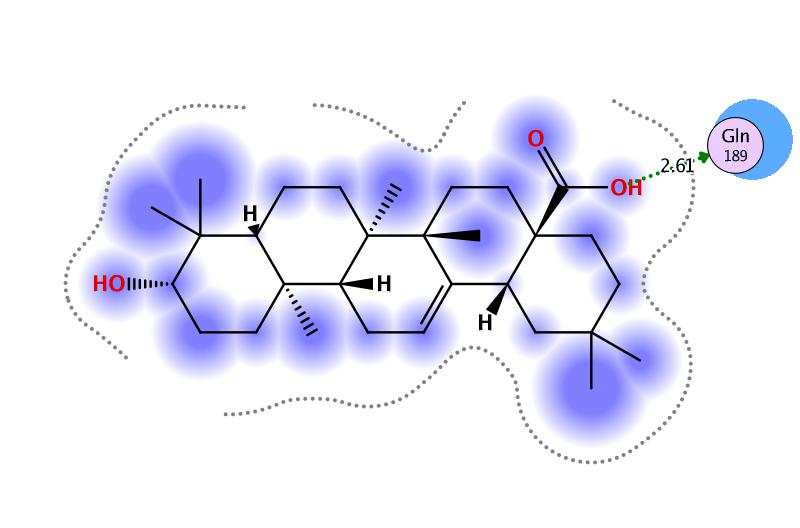 | 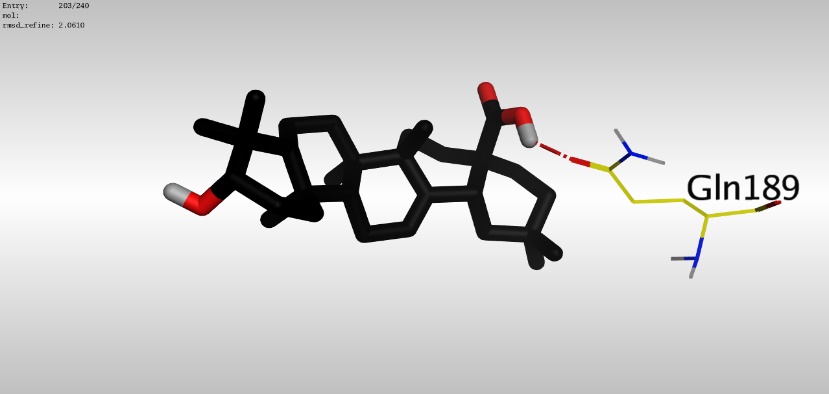 |
| **11** |  | 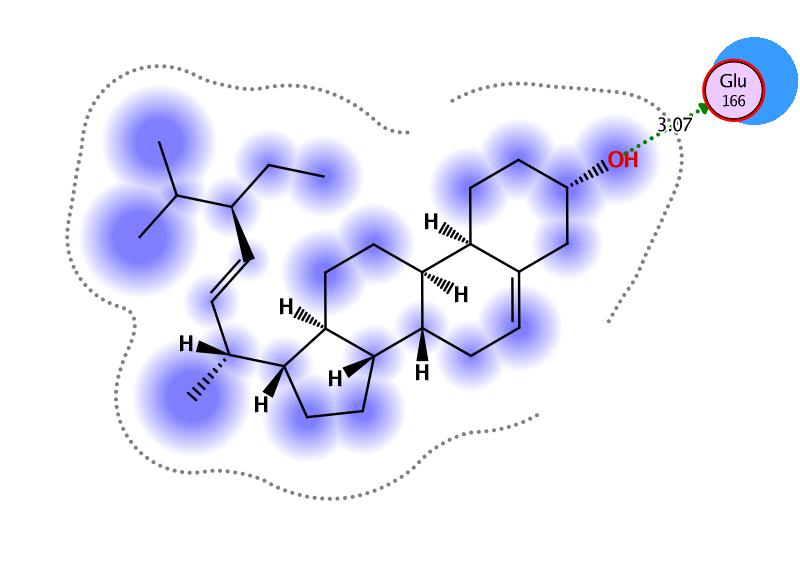 | 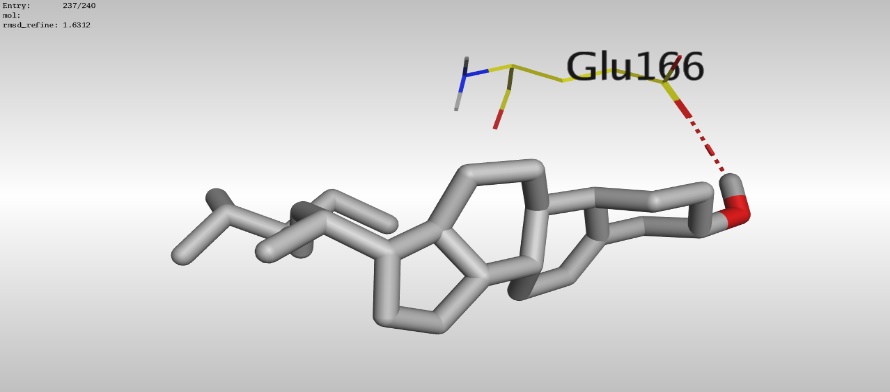 |
| **12** |  | 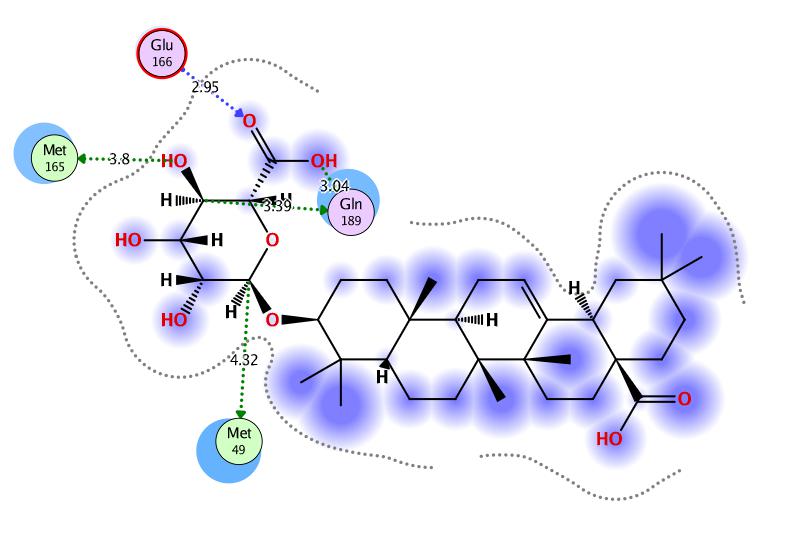 | 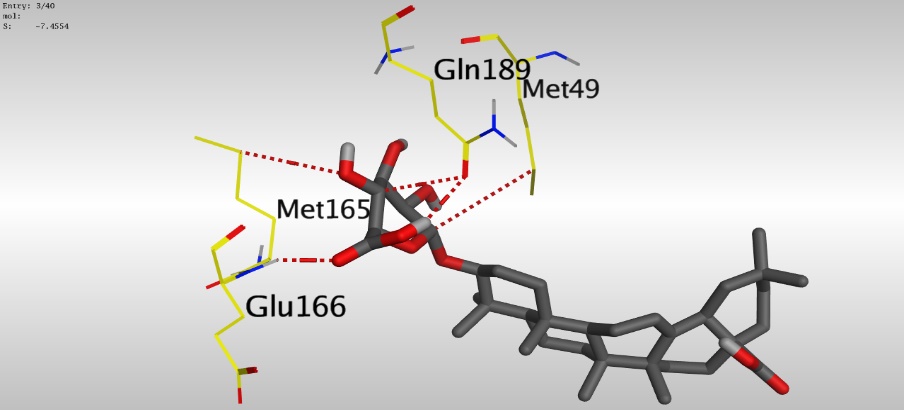 |
| **13** | N3 | 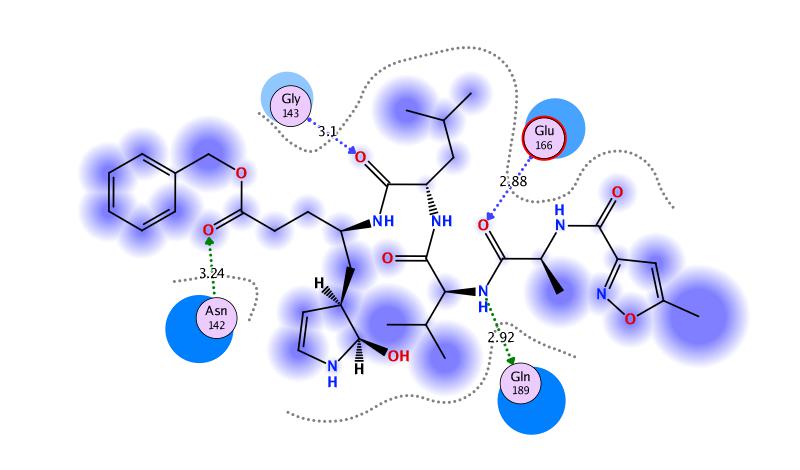 | 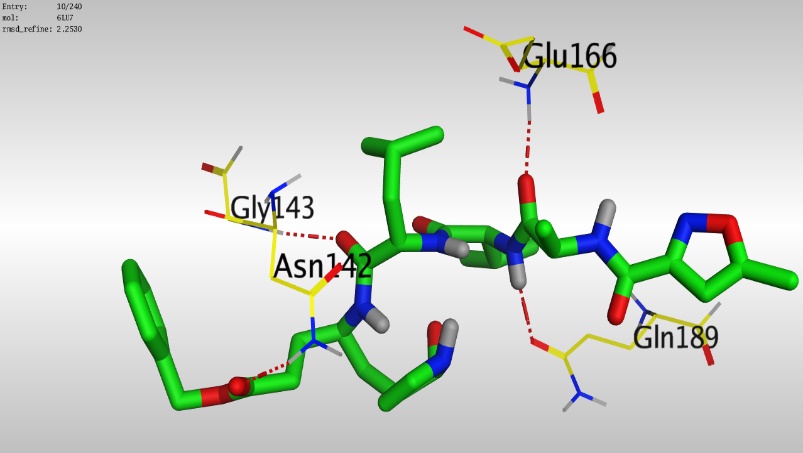 |

**Fig. SI4:  A)** Surface of M^pro^ showing the positioning and fitting of the tested compounds, and **B**) surface and maps of the tested compounds compared to N3 inhibitor

| **No.** | **Tested comp.** | **A** | **B** |
| --- | --- | --- | --- |
| **1** |  | 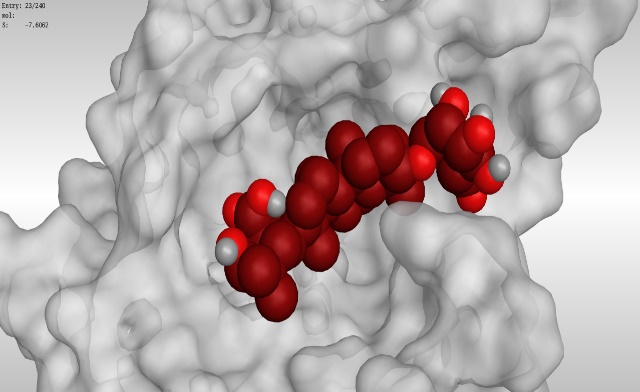 | 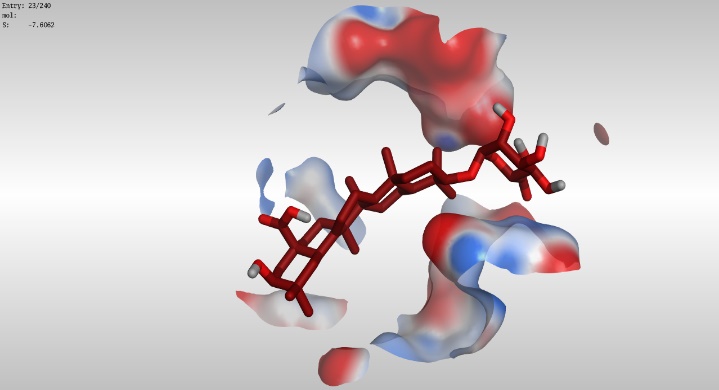 |
| **2** |  | 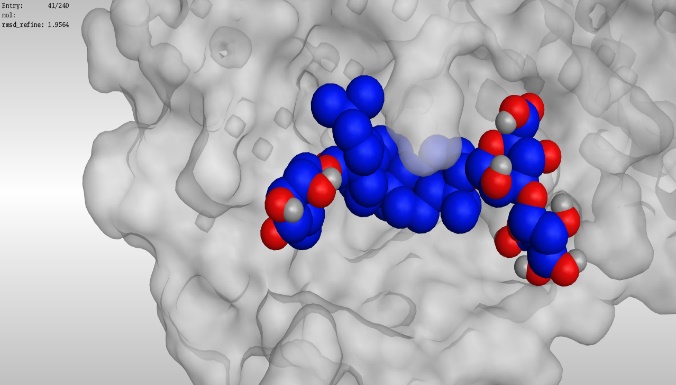 | 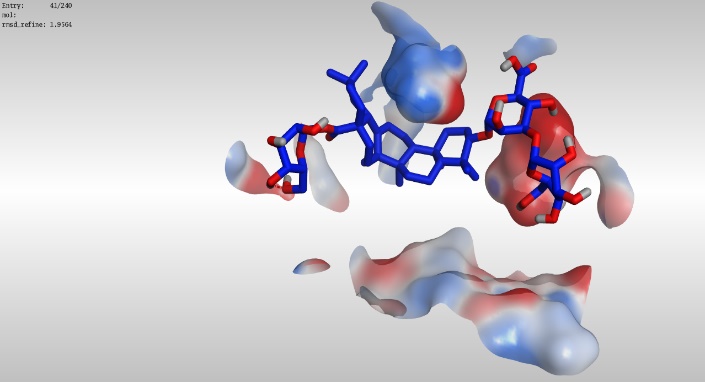 |
| **3** |  | 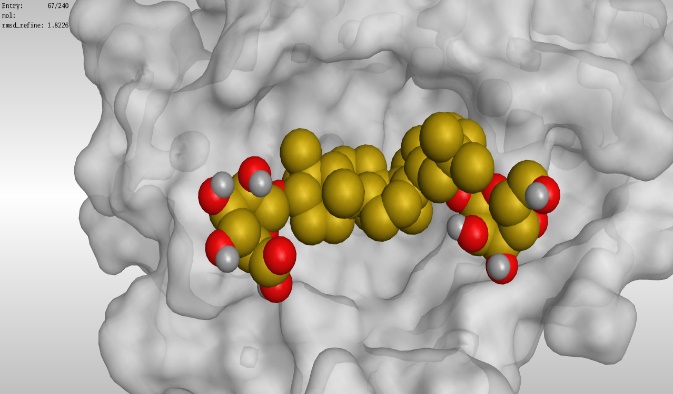 | 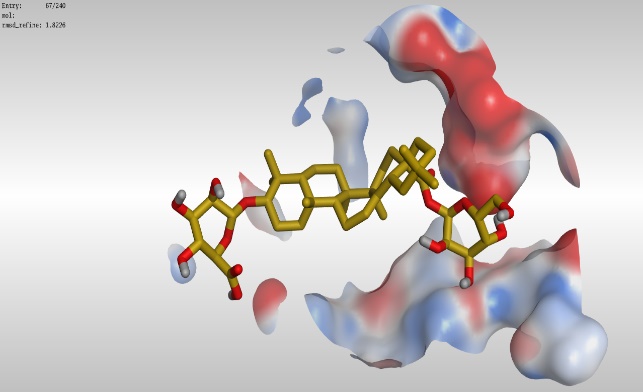 |
| **4** |  | 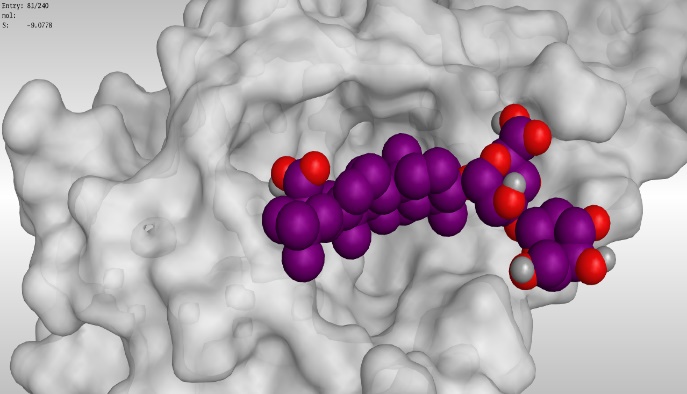 | 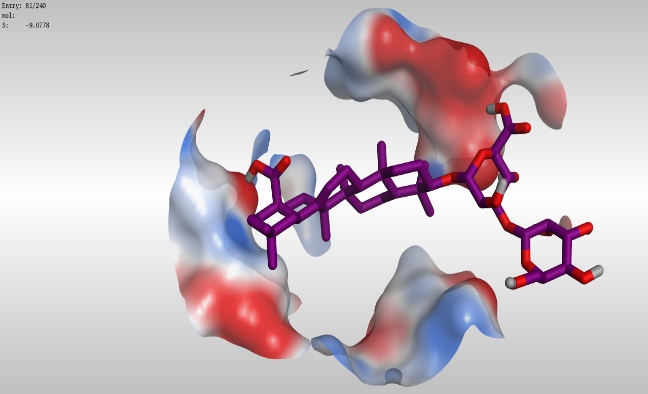 |
| **5** |  | 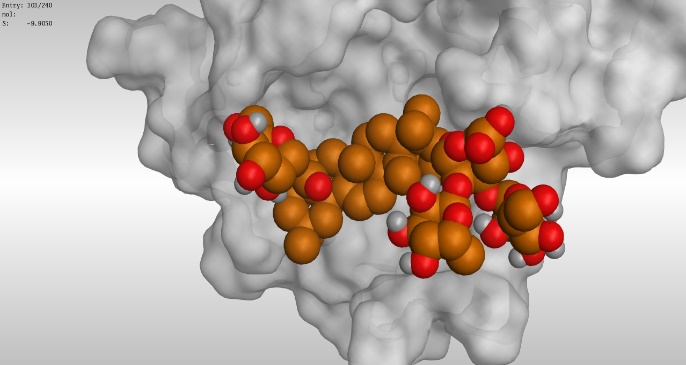 | 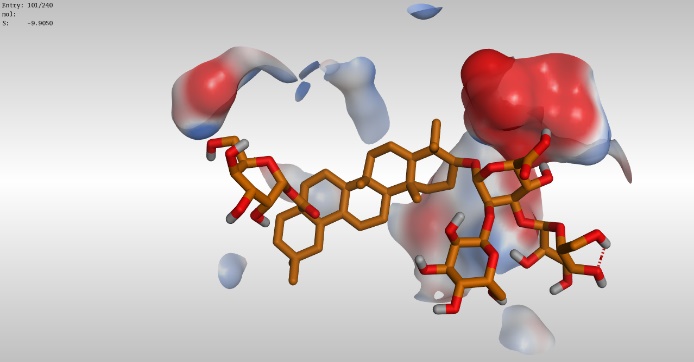 |
| **6** |  | 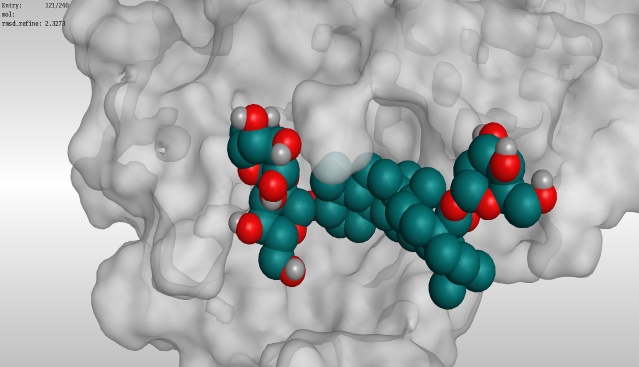 | 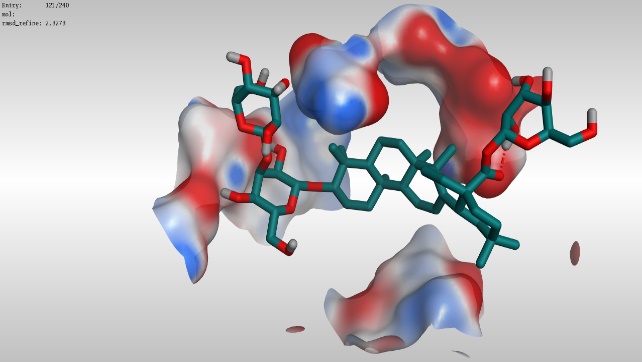 |
| **7** |  | 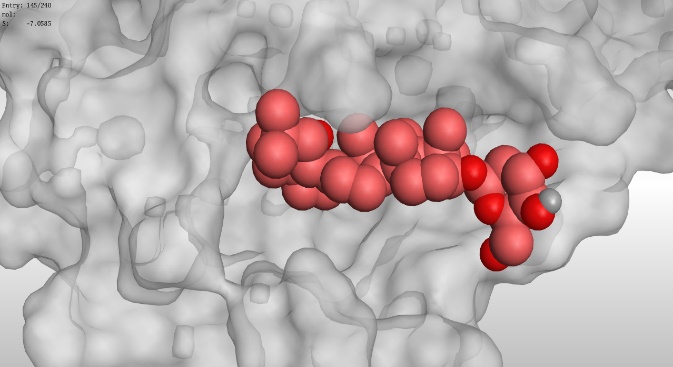 | 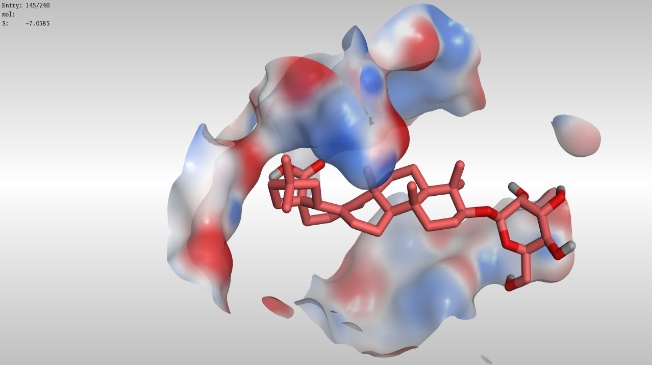 |
| **8** |  | 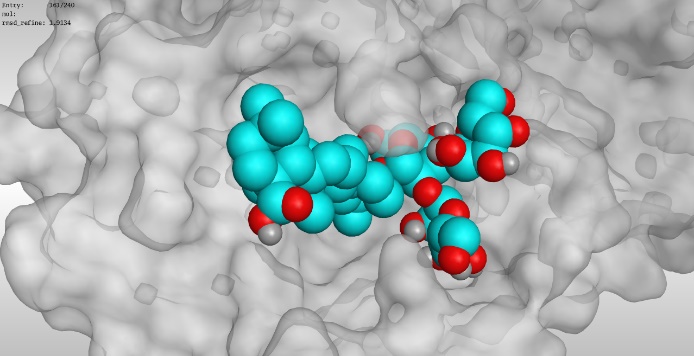 | 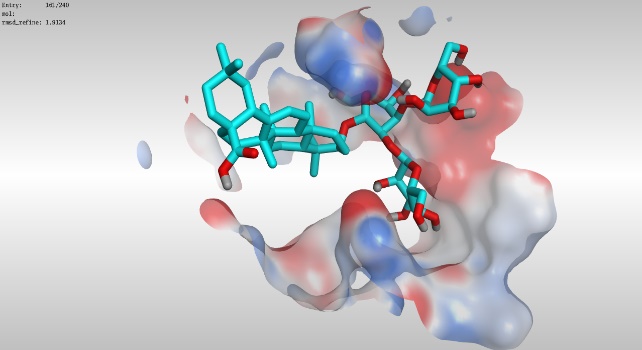 |
| **9** |  | 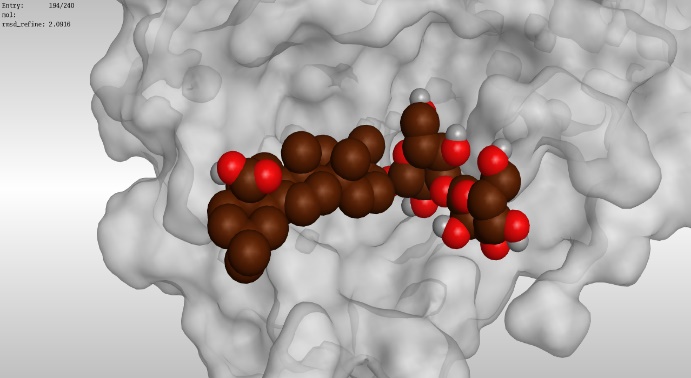 | 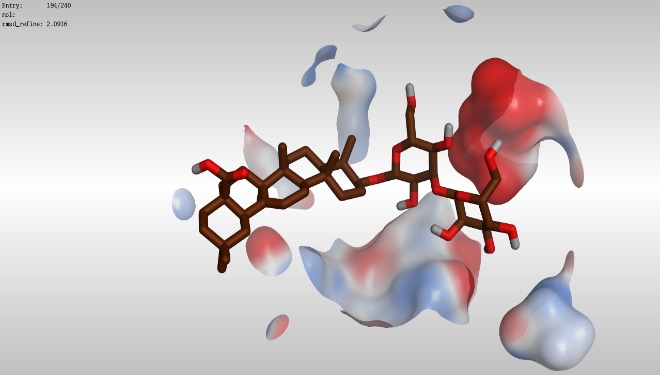 |
| **10** |  | 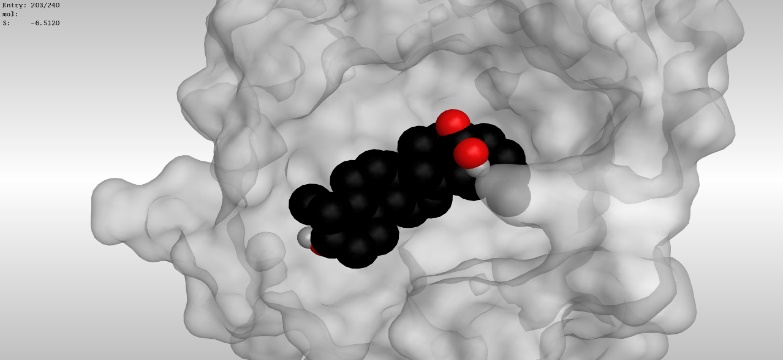 | 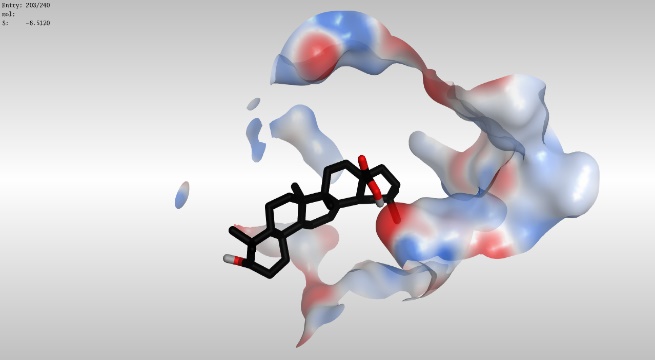 |
| **11** |  | 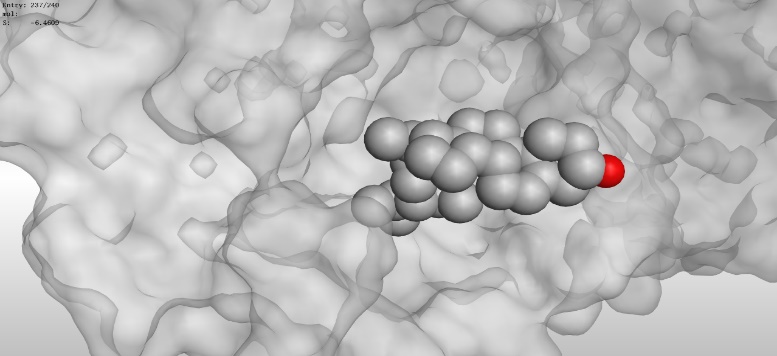 | 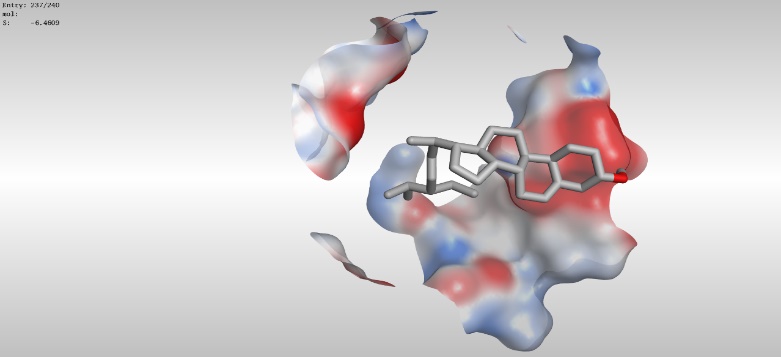 |
| **12** |  | 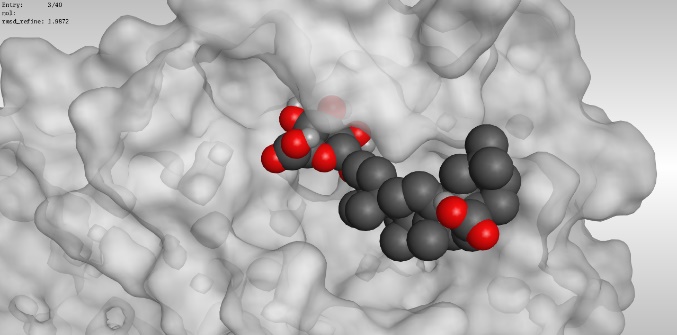 | 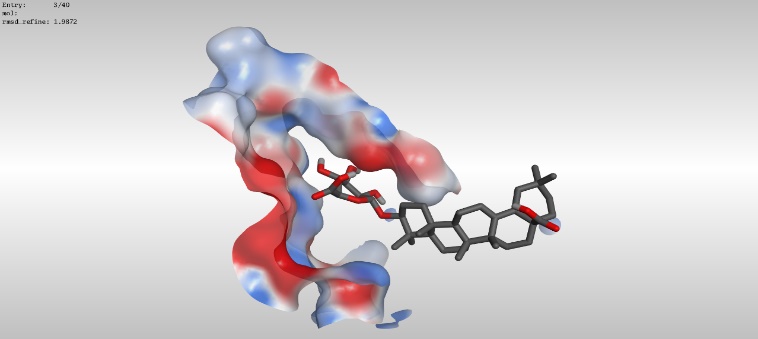 |
| **13** | N3 | 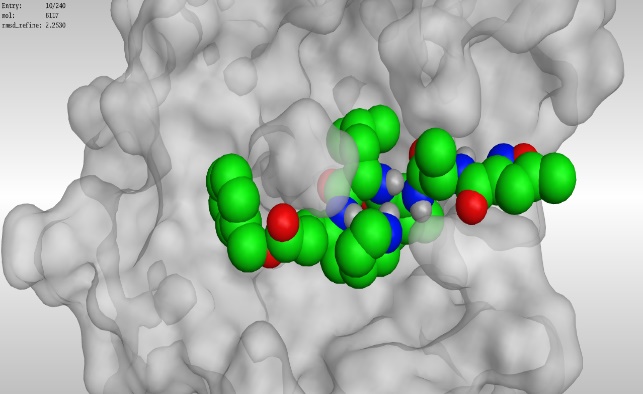 | 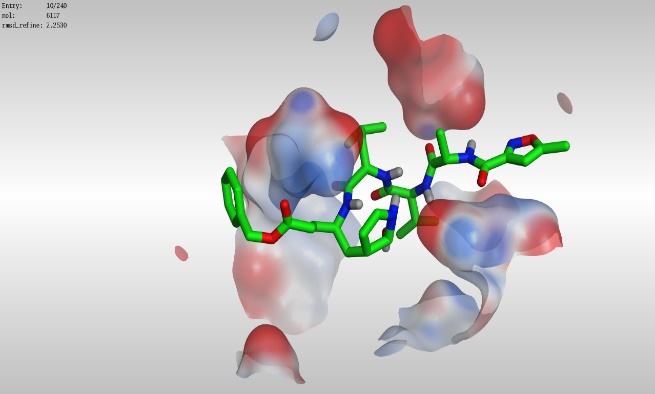 |

**Fig. SI5: Time-evolution RMSD trajectories of the three investigated ligand-protein complexes over an extended 50 ns all-atom MD simulation.** Protein-ligand complex backbone RMSD as a function of the MD simulation time (ns). Trajectories for SAP5/protein, SAP8/protein, and N3/protein complexes are represented in green, blue, and red, respectively.

**Fig. SI6:** Molecular properties, Lipinski rule and ADME studies of the isolated tested triterpenes:

**Molecule 1**


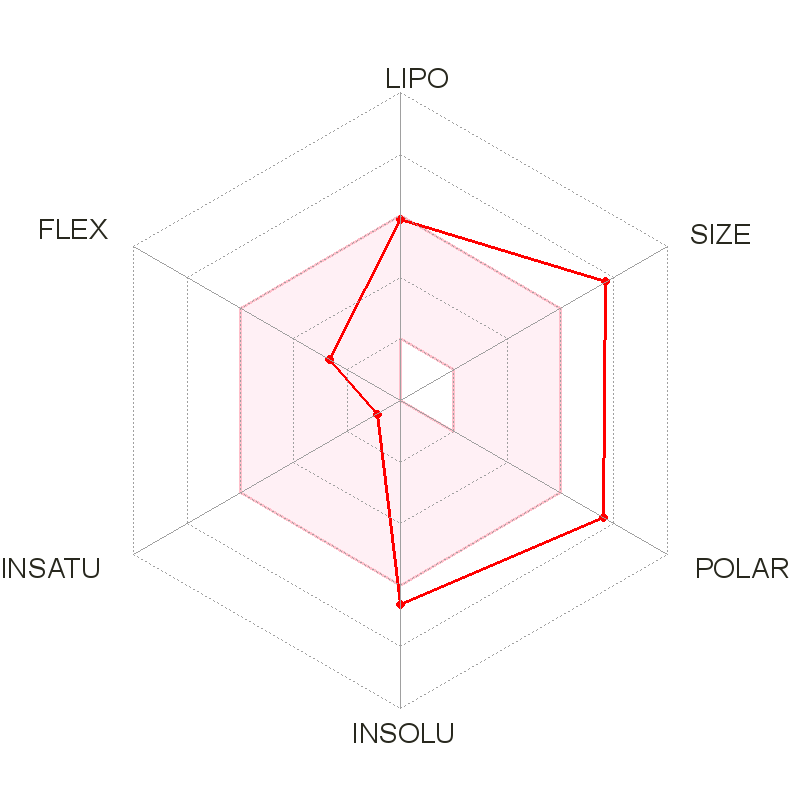


| SMILES | OC1C(O)C(OC(C1O)C(=O)O)O[C@H]1CC[C@]2(C(C1(C)C)CC[C@@]1(C2CC=C2[C@@]1(C)CC[C@@]1(C2CC(C)(C)[C@H](C1)O)C(=O)O)C)C |
| --- | --- |

| Physicochemical Properties | |
| --- | --- |
| Formula | C36H56O10 |
| Molecular weight | 648.82 g/mol |
| Num. heavy atoms | 46 |
| Num. arom. heavy atoms | 0 |
| Fraction Csp3 | 0.89 |
| Num. rotatable bonds | 4 |
| Num. H-bond acceptors | 10 |
| Num. H-bond donors | 6 |
| Molar Refractivity | 170.81 |
| TPSA [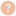](http://swissadme.ch/index.php) | 173.98 Å² |
| Lipophilicity | |
| Log *P*_o/w_ (iLOGP) [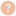](http://swissadme.ch/index.php) | 3.11 |
| Log *P*_o/w_ (XLOGP3) [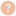](http://swissadme.ch/index.php) | 4.80 |
| Log *P*_o/w_ (WLOGP) [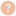](http://swissadme.ch/index.php) | 4.12 |
| Log *P*_o/w_ (MLOGP) [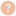](http://swissadme.ch/index.php) | 2.42 |
| Log *P*_o/w_ (SILICOS-IT) [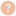](http://swissadme.ch/index.php) | 2.44 |
| Consensus Log *P*_o/w_ [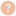](http://swissadme.ch/index.php) | 3.38 |
| Water Solubility | |
| Log *S* (ESOL) [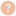](http://swissadme.ch/index.php) | -6.62 |
| Solubility | 1.55e-04 mg/ml ; 2.38e-07 mol/l |
| Class [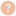](http://swissadme.ch/index.php) | Poorly soluble |
| Log *S* (Ali) [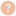](http://swissadme.ch/index.php) | -8.19 |
| Solubility | 4.23e-06 mg/ml ; 6.52e-09 mol/l |
| Class [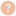](http://swissadme.ch/index.php) | Poorly soluble |
| Log *S* (SILICOS-IT) [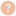](http://swissadme.ch/index.php) | -3.02 |
| Solubility | 6.21e-01 mg/ml ; 9.57e-04 mol/l |
| Class [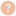](http://swissadme.ch/index.php) | Soluble |
| Pharmacokinetics | |
| GI absorption [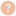](http://swissadme.ch/index.php) | Low |
| BBB permeant [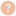](http://swissadme.ch/index.php) | No |
| P-gp substrate [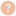](http://swissadme.ch/index.php) | Yes |
| CYP1A2 inhibitor [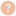](http://swissadme.ch/index.php) | No |
| CYP2C19 inhibitor [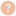](http://swissadme.ch/index.php) | No |
| CYP2C9 inhibitor [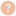](http://swissadme.ch/index.php) | No |
| CYP2D6 inhibitor [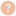](http://swissadme.ch/index.php) | No |
| CYP3A4 inhibitor [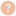](http://swissadme.ch/index.php) | No |
| Log *K*_p_ (skin permeation) [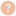](http://swissadme.ch/index.php) | -6.85 cm/s |
| Druglikeness | |
| Lipinski [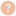](http://swissadme.ch/index.php) | No; 2 violations: MW>500, NHorOH>5 |
| Ghose [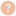](http://swissadme.ch/index.php) | No; 3 violations: MW>480, MR>130, #atoms>70 |
| Veber [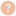](http://swissadme.ch/index.php) | No; 1 violation: TPSA>140 |
| Egan [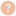](http://swissadme.ch/index.php) | No; 1 violation: TPSA>131.6 |
| Muegge [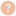](http://swissadme.ch/index.php) | No; 3 violations: MW>600, TPSA>150, H-don>5 |
| Bioavailability Score [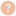](http://swissadme.ch/index.php) | 0.11 |
| Medicinal Chemistry | |
|  |  |
| PAINS [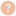](http://swissadme.ch/index.php) | 0 alert |
| Brenk [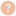](http://swissadme.ch/index.php) | 2 alerts: isolated_alkene, saponine_derivative [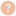](http://swissadme.ch/index.php) |
| Leadlikeness [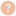](http://swissadme.ch/index.php) | No; 2 violations: MW>350, XLOGP3>3.5 |
| Synthetic accessibility [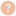](http://swissadme.ch/index.php) | 7.89 |

**Molecule 2**


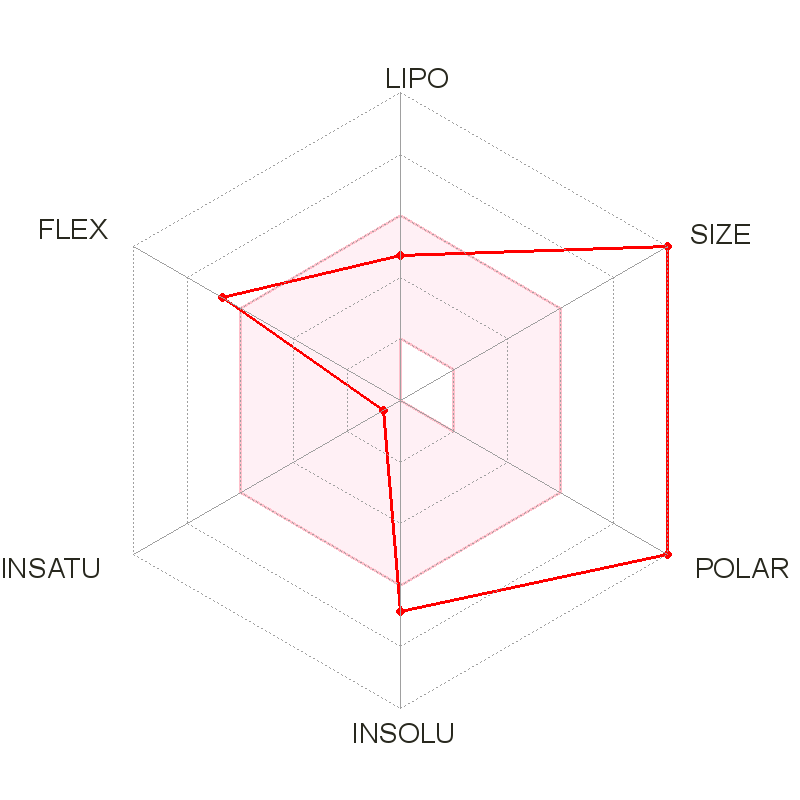


| SMILES | OCC1OC(OC2C(O)C(OC(C2O)C(=O)O)O[C@H]2CC[C@]3(C(C2(C)C)CC[C@@]2(C3CC=C3[C@@]2(C)CC[C@@]2(C3CC(C)(C)CC2)C(=O)OC2OC(CO)C(C(C2O)O)O)C)C)C(C(C1O)O)O |
| --- | --- |

| Physicochemical Properties | |
| --- | --- |
| Formula | C48H76O19 |
| Molecular weight | 957.11 g/mol |
| Num. heavy atoms | 67 |
| Num. arom. heavy atoms | 0 |
| Fraction Csp3 | 0.92 |
| Num. rotatable bonds | 10 |
| Num. H-bond acceptors | 19 |
| Num. H-bond donors | 11 |
| Molar Refractivity | 234.00 |
| TPSA [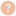](http://swissadme.ch/index.php) | 312.05 Å² |
| Lipophilicity | |
| Log *P*_o/w_ (iLOGP) [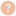](http://swissadme.ch/index.php) | 2.53 |
| Log *P*_o/w_ (XLOGP3) [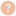](http://swissadme.ch/index.php) | 2.74 |
| Log *P*_o/w_ (WLOGP) [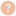](http://swissadme.ch/index.php) | 0.23 |
| Log *P*_o/w_ (MLOGP) [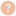](http://swissadme.ch/index.php) | -1.41 |
| Log *P*_o/w_ (SILICOS-IT) [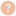](http://swissadme.ch/index.php) | -0.81 |
| Consensus Log *P*_o/w_ [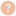](http://swissadme.ch/index.php) | 0.66 |
| Water Solubility | |
| Log *S* (ESOL) [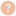](http://swissadme.ch/index.php) | -6.84 |
| Solubility | 1.38e-04 mg/ml ; 1.44e-07 mol/l |
| Class | Poorly soluble |
| Log *S* (Ali) | -8.95 |
| Solubility | 1.08e-06 mg/ml ; 1.13e-09 mol/l |
| Class | Poorly soluble |
| Log *S* (SILICOS-IT) | -0.12 |
| Solubility | 7.24e+02 mg/ml ; 7.57e-01 mol/l |
| Class | Soluble |
| Pharmacokinetics | |
| GI absorption | Low |
| BBB permeant | No |
| P-gp substrate | Yes |
| CYP1A2 inhibitor | No |
| CYP2C19 inhibitor | No |
| CYP2C9 inhibitor | No |
| CYP2D6 inhibitor | No |
| CYP3A4 inhibitor | No |
| Log *K*_p_ (skin permeation) | -10.19 cm/s |
| Druglikeness | |
| Lipinski | No; 3 violations: MW>500, NorO>10, NHorOH>5 |
| Ghose | No; 3 violations: MW>480, MR>130, #atoms>70 |
| Veber | No; 1 violation: TPSA>140 |
| Egan | No; 1 violation: TPSA>131.6 |
| Muegge | No; 5 violations: MW>600, TPSA>150, #rings>7, H-acc>10, H-don>5 |
| Bioavailability Score | 0.11 |
| Medicinal Chemistry | |
|  |  |
| PAINS | 0 alert |
| Brenk | 2 alerts: isolated_alkene, saponine_derivative |
| Leadlikeness | No; 2 violations: MW>350, Rotors>7 |
| Synthetic accessibility | 9.84 |

**Molecule 3**

| SMILES | OCC1OC(OC(=O)[C@]23CC[C@@]4(C(=CCC5[C@@]4(C)CCC4[C@]5(C)CC[C@@H](C4(C)C)OC4OC(C(=O)O)C(C(C4O)O)O)C3CC(CC2)(C)C)C)C(C(C1O)O)O |
| --- | --- |

| Physicochemical Properties | |
| --- | --- |
| Formula | C42H66O14 |
| Molecular weight | 794.97 g/mol |
| Num. heavy atoms | 56 |
| Num. arom. heavy atoms | 0 |
| Fraction Csp3 | 0.90 |
| Num. rotatable bonds | 7 |
| Num. H-bond acceptors | 14 |
| Num. H-bond donors | 8 |
| Molar Refractivity | 201.62 |
| TPSA | 232.90 Å² |
| Lipophilicity | |
| Log *P*_o/w_ (iLOGP) | 1.91 |
| Log *P*_o/w_ (XLOGP3) | 4.34 |
| Log *P*_o/w_ (WLOGP) | 2.41 |
| Log *P*_o/w_ (MLOGP) | 0.85 |
| Log *P*_o/w_ (SILICOS-IT) | 1.26 |
| Consensus Log *P*_o/w_ | 2.15 |
| Water Solubility | |
| Log *S* (ESOL) | -7.04 |
| Solubility | 7.23e-05 mg/ml ; 9.10e-08 mol/l |
| Class | Poorly soluble |
| Log *S* (Ali) | -8.95 |
| Solubility | 9.01e-07 mg/ml ; 1.13e-09 mol/l |
| Class | Poorly soluble |
| Log *S* (SILICOS-IT) | -2.00 |
| Solubility | 7.97e+00 mg/ml ; 1.00e-02 mol/l |
| Class | Soluble |
| Pharmacokinetics | |
| GI absorption | Low |
| BBB permeant | No |
| P-gp substrate | Yes |
| CYP1A2 inhibitor | No |
| CYP2C19 inhibitor | No |
| CYP2C9 inhibitor | No |
| CYP2D6 inhibitor | No |
| CYP3A4 inhibitor | No |
| Log *K*_p_ (skin permeation) | -8.07 cm/s |
| Druglikeness | |
| Lipinski | No; 3 violations: MW>500, NorO>10, NHorOH>5 |
| Ghose | No; 3 violations: MW>480, MR>130, #atoms>70 |
| Veber | No; 1 violation: TPSA>140 |
| Egan | No; 1 violation: TPSA>131.6 |
| Muegge | No; 4 violations: MW>600, TPSA>150, H-acc>10, H-don>5 |
| Bioavailability Score | 0.11 |
| Medicinal Chemistry | |
|  |  |
| PAINS | 0 alert |
| Brenk | 2 alerts: isolated_alkene, saponine_derivative |
| Leadlikeness | No; 2 violations: MW>350, XLOGP3>3.5 |
| Synthetic accessibility | 8.77 |

**Molecule 4**

| SMILES | OCC1OC(OC2C(O)C(OC(C2O)C(=O)O)O[C@H]2CC[C@]3(C(C2(C)C)CC[C@@]2(C3CC=C3[C@@]2(C)CC[C@@]2(C3CC(C)(C)CC2)C(=O)O)C)C)C(C(C1O)O)O |
| --- | --- |

| Physicochemical Properties | |
| --- | --- |
| Formula | C42H66O14 |
| Molecular weight | 794.97 g/mol |
| Num. heavy atoms | 56 |
| Num. arom. heavy atoms | 0 |
| Fraction Csp3 | 0.90 |
| Num. rotatable bonds | 7 |
| Num. H-bond acceptors | 14 |
| Num. H-bond donors | 8 |
| Molar Refractivity | 202.03 |
| TPSA | 232.90 Å² |
| Lipophilicity | |
| Log *P*_o/w_ (iLOGP) | 3.89 |
| Log *P*_o/w_ (XLOGP3) | 4.55 |
| Log *P*_o/w_ (WLOGP) | 2.97 |
| Log *P*_o/w_ (MLOGP) | 0.85 |
| Log *P*_o/w_ (SILICOS-IT) | 1.26 |
| Consensus Log *P*_o/w_ | 2.71 |
| Water Solubility | |
| Log *S* (ESOL) | -7.17 |
| Solubility | 5.33e-05 mg/ml ; 6.71e-08 mol/l |
| Class | Poorly soluble |
| Log *S* (Ali) | -9.16 |
| Solubility | 5.45e-07 mg/ml ; 6.86e-10 mol/l |
| Class | Poorly soluble |
| Log *S* (SILICOS-IT) | -2.00 |
| Solubility | 7.97e+00 mg/ml ; 1.00e-02 mol/l |
| Class | Soluble |
| Pharmacokinetics | |
| GI absorption | Low |
| BBB permeant | No |
| P-gp substrate | Yes |
| CYP1A2 inhibitor | No |
| CYP2C19 inhibitor | No |
| CYP2C9 inhibitor | No |
| CYP2D6 inhibitor | No |
| CYP3A4 inhibitor | No |
| Log *K*_p_ (skin permeation) | -7.92 cm/s |
| Druglikeness | |
| Lipinski | No; 3 violations: MW>500, NorO>10, NHorOH>5 |
| Ghose | No; 3 violations: MW>480, MR>130, #atoms>70 |
| Veber | No; 1 violation: TPSA>140 |
| Egan | No; 1 violation: TPSA>131.6 |
| Muegge | No; 4 violations: MW>600, TPSA>150, H-acc>10, H-don>5 |
| Bioavailability Score | 0.11 |
| Medicinal Chemistry | |
|  |  |
| PAINS | 0 alert |
| Brenk | 2 alerts: isolated_alkene, saponine_derivative |
| Leadlikeness | No; 2 violations: MW>350, XLOGP3>3.5 |
| Synthetic accessibility | 8.86 |

**Molecule 5**

| SMILES | OCC1OC(OC2C(OC(C(C2OC2OC(CO)C(C(C2O)O)O)O)C(=O)O)O[C@H]2CC[C@]3(C(C2(C)C)CC[C@@]2(C3CC=C3[C@@]2(C)CC[C@@]2(C3CC(C)(C)CC2)C(=O)OC2OC(CO)C(C(C2O)O)O)C)C)C(C(C1O)O)O |
| --- | --- |

| Physicochemical Properties | |
| --- | --- |
| Formula | C54H86O24 |
| Molecular weight | 1119.25 g/mol |
| Num. heavy atoms | 78 |
| Num. arom. heavy atoms | 0 |
| Fraction Csp3 | 0.93 |
| Num. rotatable bonds | 13 |
| Num. H-bond acceptors | 24 |
| Num. H-bond donors | 14 |
| Molar Refractivity | 266.39 |
| TPSA | 391.20 Å² |
| Lipophilicity | |
| Log *P*_o/w_ (iLOGP) | 3.05 |
| Log *P*_o/w_ (XLOGP3) | 1.15 |
| Log *P*_o/w_ (WLOGP) | -1.94 |
| Log *P*_o/w_ (MLOGP) | -3.62 |
| Log *P*_o/w_ (SILICOS-IT) | -2.89 |
| Consensus Log *P*_o/w_ | -0.85 |
| Water Solubility | |
| Log *S* (ESOL) | -6.65 |
| Solubility | 2.53e-04 mg/ml ; 2.26e-07 mol/l |
| Class | Poorly soluble |
| Log *S* (Ali) | -8.96 |
| Solubility | 1.23e-06 mg/ml ; 1.10e-09 mol/l |
| Class | Poorly soluble |
| Log *S* (SILICOS-IT) | 1.78 |
| Solubility | 6.76e+04 mg/ml ; 6.04e+01 mol/l |
| Class | Soluble |
| Pharmacokinetics | |
| GI absorption | Low |
| BBB permeant | No |
| P-gp substrate | Yes |
| CYP1A2 inhibitor | No |
| CYP2C19 inhibitor | No |
| CYP2C9 inhibitor | No |
| CYP2D6 inhibitor | No |
| CYP3A4 inhibitor | No |
| Log *K*_p_ (skin permeation) | -12.31 cm/s |
| Druglikeness | |
| Lipinski | No; 3 violations: MW>500, NorO>10, NHorOH>5 |
| Ghose | No; 4 violations: MW>480, WLOGP<-0.4, MR>130, #atoms>70 |
| Veber | No; 2 violations: Rotors>10, TPSA>140 |
| Egan | No; 1 violation: TPSA>131.6 |
| Muegge | No; 5 violations: MW>600, TPSA>150, #rings>7, H-acc>10, H-don>5 |
| Bioavailability Score | 0.11 |
| Medicinal Chemistry | |
|  |  |
| PAINS | 0 alert |
| Brenk | 2 alerts: isolated_alkene, saponine_derivative |
| Leadlikeness | No; 2 violations: MW>350, Rotors>7 |
| Synthetic accessibility | 10.00 |

**Molecule 6**

| SMILES | OCC1OC(O[C@H]2CC[C@]3(C(C2(C)C)CC[C@@]2(C3CC=C3[C@@]2(C)CC[C@@]2(C3CC(C)(C)CC2)C(=O)OC2OC(CO)C(C(C2O)O)O)C)C)C(C(C1O)OC1OC(CO)C(C(C1O)O)O)O |
| --- | --- |

| Physicochemical Properties | |
| --- | --- |
| Formula | C48H78O18 |
| Molecular weight | 943.12 g/mol |
| Num. heavy atoms | 66 |
| Num. arom. heavy atoms | 0 |
| Fraction Csp3 | 0.94 |
| Num. rotatable bonds | 10 |
| Num. H-bond acceptors | 18 |
| Num. H-bond donors | 11 |
| Molar Refractivity | 233.39 |
| TPSA | 294.98 Å² |
| Lipophilicity | |
| Log *P*_o/w_ (iLOGP) | 4.25 |
| Log *P*_o/w_ (XLOGP3) | 2.49 |
| Log *P*_o/w_ (WLOGP) | 0.14 |
| Log *P*_o/w_ (MLOGP) | -1.40 |
| Log *P*_o/w_ (SILICOS-IT) | -0.37 |
| Consensus Log *P*_o/w_ | 1.02 |
| Water Solubility | |
| Log *S* (ESOL) | -6.60 |
| Solubility | 2.39e-04 mg/ml ; 2.53e-07 mol/l |
| Class | Poorly soluble |
| Log *S* (Ali) | -8.33 |
| Solubility | 4.41e-06 mg/ml ; 4.68e-09 mol/l |
| Class | Poorly soluble |
| Log *S* (SILICOS-IT) | -0.60 |
| Solubility | 2.36e+02 mg/ml ; 2.51e-01 mol/l |
| Class | Soluble |
| Pharmacokinetics | |
| GI absorption | Low |
| BBB permeant | No |
| P-gp substrate | Yes |
| CYP1A2 inhibitor | No |
| CYP2C19 inhibitor | No |
| CYP2C9 inhibitor | No |
| CYP2D6 inhibitor | No |
| CYP3A4 inhibitor | No |
| Log *K*_p_ (skin permeation) | -10.29 cm/s |
| Druglikeness | |
| Lipinski | No; 3 violations: MW>500, NorO>10, NHorOH>5 |
| Ghose | No; 3 violations: MW>480, MR>130, #atoms>70 |
| Veber | No; 1 violation: TPSA>140 |
| Egan | No; 1 violation: TPSA>131.6 |
| Muegge | No; 5 violations: MW>600, TPSA>150, #rings>7, H-acc>10, H-don>5 |
| Bioavailability Score | 0.17 |
| Medicinal Chemistry | |
|  |  |
| PAINS | 0 alert |
| Brenk | 2 alerts: isolated_alkene, saponine_derivative |
| Leadlikeness | No; 2 violations: MW>350, Rotors>7 |
| Synthetic accessibility | 9.84 |

**Molecule 7**

| SMILES | OCC1OC(O[C@H]2CC[C@]3(C(C2(C)C)CC[C@@]2(C3CC=C3[C@@]2(C)CC[C@@]2(C3CC(C)(C)CC2)C(=O)O)C)C)C(C(C1O)O)O |
| --- | --- |

| Physicochemical Properties | |
| --- | --- |
| Formula | C36H58O8 |
| Molecular weight | 618.84 g/mol |
| Num. heavy atoms | 44 |
| Num. arom. heavy atoms | 0 |
| Fraction Csp3 | 0.92 |
| Num. rotatable bonds | 4 |
| Num. H-bond acceptors | 8 |
| Num. H-bond donors | 5 |
| Molar Refractivity | 169.04 |
| TPSA | 136.68 Å² |
| Lipophilicity | |
| Log *P*_o/w_ (iLOGP) | 4.14 |
| Log *P*_o/w_ (XLOGP3) | 5.89 |
| Log *P*_o/w_ (WLOGP) | 5.06 |
| Log *P*_o/w_ (MLOGP) | 3.25 |
| Log *P*_o/w_ (SILICOS-IT) | 3.77 |
| Consensus Log *P*_o/w_ | 4.42 |
| Water Solubility | |
| Log *S* (ESOL) | -7.12 |
| Solubility | 4.66e-05 mg/ml ; 7.52e-08 mol/l |
| Class | Poorly soluble |
| Log *S* (Ali) | -8.53 |
| Solubility | 1.81e-06 mg/ml ; 2.93e-09 mol/l |
| Class | Poorly soluble |
| Log *S* (SILICOS-IT) | -4.32 |
| Solubility | 2.95e-02 mg/ml ; 4.76e-05 mol/l |
| Class | Moderately soluble |
| Pharmacokinetics | |
| GI absorption | Low |
| BBB permeant | No |
| P-gp substrate | Yes |
| CYP1A2 inhibitor | No |
| CYP2C19 inhibitor | No |
| CYP2C9 inhibitor | No |
| CYP2D6 inhibitor | No |
| CYP3A4 inhibitor | No |
| Log *K*_p_ (skin permeation) | -5.89 cm/s |
| Druglikeness | |
| Lipinski | Yes; 1 violation: MW>500 |
| Ghose | No; 3 violations: MW>480, MR>130, #atoms>70 |
| Veber | Yes |
| Egan | No; 1 violation: TPSA>131.6 |
| Muegge | No; 2 violations: MW>600, XLOGP3>5 |
| Bioavailability Score | 0.56 |
| Medicinal Chemistry | |
|  |  |
| PAINS | 0 alert |
| Brenk | 2 alerts: isolated_alkene, saponine_derivative |
| Leadlikeness | No; 2 violations: MW>350, XLOGP3>3.5 |
| Synthetic accessibility | 7.78 |

**Molecule 8**

| SMILES | OCC1OC(O[C@H]2CC[C@]3(C(C2(C)C)CC[C@@]2(C3CC=C3[C@@]2(C)CC[C@@]2(C3CC(C)(C)CC2)C(=O)O)C)C)C(C(C1O)OC1OC(CO)C(C(C1O)O)O)OC1OC(CO)C(C(C1O)O)O |
| --- | --- |

| Physicochemical Properties | |
| --- | --- |
| Formula | C48H78O18 |
| Molecular weight | 943.12 g/mol |
| Num. heavy atoms | 66 |
| Num. arom. heavy atoms | 0 |
| Fraction Csp3 | 0.94 |
| Num. rotatable bonds | 10 |
| Num. H-bond acceptors | 18 |
| Num. H-bond donors | 11 |
| Molar Refractivity | 233.80 |
| TPSA | 294.98 Å² |
| Lipophilicity | |
| Log *P*_o/w_ (iLOGP) | 3.47 |
| Log *P*_o/w_ (XLOGP3) | 2.70 |
| Log *P*_o/w_ (WLOGP) | 0.71 |
| Log *P*_o/w_ (MLOGP) | -1.40 |
| Log *P*_o/w_ (SILICOS-IT) | -0.37 |
| Consensus Log *P*_o/w_ | 1.02 |
| Water Solubility | |
| Log *S* (ESOL) | -6.73 |
| Solubility | 1.76e-04 mg/ml ; 1.87e-07 mol/l |
| Class | Poorly soluble |
| Log *S* (Ali) | -8.55 |
| Solubility | 2.67e-06 mg/ml ; 2.83e-09 mol/l |
| Class | Poorly soluble |
| Log *S* (SILICOS-IT) | -0.60 |
| Solubility | 2.36e+02 mg/ml ; 2.51e-01 mol/l |
| Class | Soluble |
| Pharmacokinetics | |
| GI absorption | Low |
| BBB permeant | No |
| P-gp substrate | Yes |
| CYP1A2 inhibitor | No |
| CYP2C19 inhibitor | No |
| CYP2C9 inhibitor | No |
| CYP2D6 inhibitor | No |
| CYP3A4 inhibitor | No |
| Log *K*_p_ (skin permeation) | -10.14 cm/s |
| Druglikeness | |
| Lipinski | No; 3 violations: MW>500, NorO>10, NHorOH>5 |
| Ghose | No; 3 violations: MW>480, MR>130, #atoms>70 |
| Veber | No; 1 violation: TPSA>140 |
| Egan | No; 1 violation: TPSA>131.6 |
| Muegge | No; 5 violations: MW>600, TPSA>150, #rings>7, H-acc>10, H-don>5 |
| Bioavailability Score | 0.11 |
| Medicinal Chemistry | |
|  |  |
| PAINS | 0 alert |
| Brenk | 2 alerts: isolated_alkene, saponine_derivative |
| Leadlikeness | No; 2 violations: MW>350, Rotors>7 |
| Synthetic accessibility | 9.99 |

**Molecule 9**

| SMILES | OCC1OC(O[C@H]2CC[C@]3(C(C2(C)C)CC[C@@]2(C3CC=C3[C@@]2(C)CC[C@@]2(C3CC(C)(C)CC2)C(=O)O)C)C)C(C(C1O)OC1OC(CO)C(C(C1O)O)O)O |
| --- | --- |

| Physicochemical Properties | |
| --- | --- |
| Formula | C42H68O13 |
| Molecular weight | 780.98 g/mol |
| Num. heavy atoms | 55 |
| Num. arom. heavy atoms | 0 |
| Fraction Csp3 | 0.93 |
| Num. rotatable bonds | 7 |
| Num. H-bond acceptors | 13 |
| Num. H-bond donors | 8 |
| Molar Refractivity | 201.42 |
| TPSA | 215.83 Å² |
| Lipophilicity | |
| Log *P*_o/w_ (iLOGP) | 4.11 |
| Log *P*_o/w_ (XLOGP3) | 4.30 |
| Log *P*_o/w_ (WLOGP) | 2.88 |
| Log *P*_o/w_ (MLOGP) | 0.88 |
| Log *P*_o/w_ (SILICOS-IT) | 1.70 |
| Consensus Log *P*_o/w_ | 2.78 |
| Water Solubility | |
| Log *S* (ESOL) | -6.93 |
| Solubility | 9.20e-05 mg/ml ; 1.18e-07 mol/l |
| Class | Poorly soluble |
| Log *S* (Ali) | -8.55 |
| Solubility | 2.22e-06 mg/ml ; 2.85e-09 mol/l |
| Class | Poorly soluble |
| Log *S* (SILICOS-IT) | -2.48 |
| Solubility | 2.60e+00 mg/ml ; 3.33e-03 mol/l |
| Class | Soluble |
| Pharmacokinetics | |
| GI absorption | Low |
| BBB permeant | No |
| P-gp substrate | Yes |
| CYP1A2 inhibitor | No |
| CYP2C19 inhibitor | No |
| CYP2C9 inhibitor | No |
| CYP2D6 inhibitor | No |
| CYP3A4 inhibitor | No |
| Log *K*_p_ (skin permeation) | -8.01 cm/s |
| Druglikeness | |
| Lipinski | No; 3 violations: MW>500, NorO>10, NHorOH>5 |
| Ghose | No; 3 violations: MW>480, MR>130, #atoms>70 |
| Veber | No; 1 violation: TPSA>140 |
| Egan | No; 1 violation: TPSA>131.6 |
| Muegge | No; 4 violations: MW>600, TPSA>150, H-acc>10, H-don>5 |
| Bioavailability Score | 0.11 |
| Medicinal Chemistry | |
|  |  |
| PAINS | 0 alert |
| Brenk | 2 alerts: isolated_alkene, saponine_derivative |
| Leadlikeness | No; 2 violations: MW>350, XLOGP3>3.5 |
| Synthetic accessibility | 8.86 |

**Molecule 10**

| SMILES | O[C@H]1CC[C@]2(C(C1(C)C)CC[C@@]1(C2CC=C2[C@@]1(C)CC[C@@]1(C2CC(C)(C)CC1)C(=O)O)C)C |
| --- | --- |

| Physicochemical Properties | |
| --- | --- |
| Formula | C30H48O3 |
| Molecular weight | 456.70 g/mol |
| Num. heavy atoms | 33 |
| Num. arom. heavy atoms | 0 |
| Fraction Csp3 | 0.90 |
| Num. rotatable bonds | 1 |
| Num. H-bond acceptors | 3 |
| Num. H-bond donors | 2 |
| Molar Refractivity | 136.65 |
| TPSA | 57.53 Å² |
| Lipophilicity | |
| Log *P*_o/w_ (iLOGP) | 3.94 |
| Log *P*_o/w_ (XLOGP3) | 7.49 |
| Log *P*_o/w_ (WLOGP) | 7.23 |
| Log *P*_o/w_ (MLOGP) | 5.82 |
| Log *P*_o/w_ (SILICOS-IT) | 5.85 |
| Consensus Log *P*_o/w_ | 6.07 |
| Water Solubility | |
| Log *S* (ESOL) | -7.32 |
| Solubility | 2.16e-05 mg/ml ; 4.74e-08 mol/l |
| Class | Poorly soluble |
| Log *S* (Ali) | -8.53 |
| Solubility | 1.34e-06 mg/ml ; 2.94e-09 mol/l |
| Class | Poorly soluble |
| Log *S* (SILICOS-IT) | -6.12 |
| Solubility | 3.45e-04 mg/ml ; 7.55e-07 mol/l |
| Class | Poorly soluble |
| Pharmacokinetics | |
| GI absorption | Low |
| BBB permeant | No |
| P-gp substrate | No |
| CYP1A2 inhibitor | No |
| CYP2C19 inhibitor | No |
| CYP2C9 inhibitor | No |
| CYP2D6 inhibitor | No |
| CYP3A4 inhibitor | No |
| Log *K*_p_ (skin permeation) | -3.77 cm/s |
| Druglikeness | |
| Lipinski | Yes; 1 violation: MLOGP>4.15 |
| Ghose | No; 3 violations: WLOGP>5.6, MR>130, #atoms>70 |
| Veber | Yes |
| Egan | No; 1 violation: WLOGP>5.88 |
| Muegge | No; 1 violation: XLOGP3>5 |
| Bioavailability Score | 0.85 |
| Medicinal Chemistry | |
|  |  |
| PAINS | 0 alert |
| Brenk | 1 alert: isolated_alkene |
| Leadlikeness | No; 2 violations: MW>350, XLOGP3>3.5 |
| Synthetic accessibility | 6.08 |

**Molecule 11**

| SMILES | CCC(C(C)C)/C=C/[C@H](C1CCC2C1CCC1C2CC=C2C1CC[C@@H](C2)O)C |
| --- | --- |

| Physicochemical Properties | |
| --- | --- |
| Formula | C27H44O |
| Molecular weight | 384.64 g/mol |
| Num. heavy atoms | 28 |
| Num. arom. heavy atoms | 0 |
| Fraction Csp3 | 0.85 |
| Num. rotatable bonds | 5 |
| Num. H-bond acceptors | 1 |
| Num. H-bond donors | 1 |
| Molar Refractivity | 123.66 |
| TPSA | 20.23 Å² |
| Lipophilicity | |
| Log *P*_o/w_ (iLOGP) | 4.64 |
| Log *P*_o/w_ (XLOGP3) | 7.62 |
| Log *P*_o/w_ (WLOGP) | 7.02 |
| Log *P*_o/w_ (MLOGP) | 6.23 |
| Log *P*_o/w_ (SILICOS-IT) | 5.83 |
| Consensus Log *P*_o/w_ | 6.27 |
| Water Solubility | |
| Log *S* (ESOL) | -6.70 |
| Solubility | 7.76e-05 mg/ml ; 2.02e-07 mol/l |
| Class | Poorly soluble |
| Log *S* (Ali) | -7.88 |
| Solubility | 5.03e-06 mg/ml ; 1.31e-08 mol/l |
| Class | Poorly soluble |
| Log *S* (SILICOS-IT) | -4.28 |
| Solubility | 2.01e-02 mg/ml ; 5.24e-05 mol/l |
| Class | Moderately soluble |
| Pharmacokinetics | |
| GI absorption | Low |
| BBB permeant | No |
| P-gp substrate | No |
| CYP1A2 inhibitor | No |
| CYP2C19 inhibitor | No |
| CYP2C9 inhibitor | Yes |
| CYP2D6 inhibitor | No |
| CYP3A4 inhibitor | No |
| Log *K*_p_ (skin permeation) | -3.24 cm/s |
| Druglikeness | |
| Lipinski | Yes; 1 violation: MLOGP>4.15 |
| Ghose | No; 2 violations: WLOGP>5.6, #atoms>70 |
| Veber | Yes |
| Egan | No; 1 violation: WLOGP>5.88 |
| Muegge | No; 2 violations: XLOGP3>5, Heteroatoms<2 |
| Bioavailability Score | 0.55 |
| Medicinal Chemistry | |
|  |  |
| PAINS | 0 alert |
| Brenk | 1 alert: isolated_alkene |
| Leadlikeness | No; 2 violations: MW>350, XLOGP3>3.5 |
| Synthetic accessibility | 5.97 |

**Molecule 12**

| SMILES | OC1C(O)C(OC(C1O)C(=O)O)O[C@H]1CC[C@]2(C(C1(C)C)CC[C@@]1(C2CC=C2[C@@]1(C)CC[C@@]1(C2CC(C)(C)CC1)C(=O)O)C)C |
| --- | --- |

| Physicochemical Properties | |
| --- | --- |
| Formula | C36H56O9 |
| Molecular weight | 632.82 g/mol |
| Num. heavy atoms | 45 |
| Num. arom. heavy atoms | 0 |
| Fraction Csp3 | 0.89 |
| Num. rotatable bonds | 4 |
| Num. H-bond acceptors | 9 |
| Num. H-bond donors | 5 |
| Molar Refractivity | 169.65 |
| TPSA | 153.75 Å² |
| Lipophilicity | |
| Log *P*_o/w_ (iLOGP) | 3.77 |
| Log *P*_o/w_ (XLOGP3) | 6.14 |
| Log *P*_o/w_ (WLOGP) | 5.15 |
| Log *P*_o/w_ (MLOGP) | 3.19 |
| Log *P*_o/w_ (SILICOS-IT) | 3.33 |
| Consensus Log *P*_o/w_ | 4.32 |
| Water Solubility | |
| Log *S* (ESOL) | -7.37 |
| Solubility | 2.71e-05 mg/ml ; 4.29e-08 mol/l |
| Class | Poorly soluble |
| Log *S* (Ali) | -9.15 |
| Solubility | 4.47e-07 mg/ml ; 7.06e-10 mol/l |
| Class | Poorly soluble |
| Log *S* (SILICOS-IT) | -3.85 |
| Solubility | 9.00e-02 mg/ml ; 1.42e-04 mol/l |
| Class | Soluble |
| Pharmacokinetics | |
| GI absorption | Low |
| BBB permeant | No |
| P-gp substrate | Yes |
| CYP1A2 inhibitor | No |
| CYP2C19 inhibitor | No |
| CYP2C9 inhibitor | No |
| CYP2D6 inhibitor | No |
| CYP3A4 inhibitor | No |
| Log *K*_p_ (skin permeation) | -5.80 cm/s |
| Druglikeness | |
| Lipinski | Yes; 1 violation: MW>500 |
| Ghose | No; 3 violations: MW>480, MR>130, #atoms>70 |
| Veber | No; 1 violation: TPSA>140 |
| Egan | No; 1 violation: TPSA>131.6 |
| Muegge | No; 3 violations: MW>600, XLOGP3>5, TPSA>150 |
| Bioavailability Score | 0.11 |
| Medicinal Chemistry | |
|  |  |
| PAINS | 0 alert |
| Brenk | 2 alerts: isolated_alkene, saponine_derivative |
| Leadlikeness | No; 2 violations: MW>350, XLOGP3>3.5 |
| Synthetic accessibility | 7.78 |

**Fig. SI7:** The BOILED-Egg representation of the isolated tested triterpenes:
